# H2AFZ Is a Prognostic Biomarker Correlated to TP53 Mutation and Immune Infiltration in Hepatocellular Carcinoma

**DOI:** 10.3389/fonc.2021.701736

**Published:** 2021-10-25

**Authors:** Mingwei Dong, Jing Chen, Yiran Deng, Danying Zhang, Ling Dong, Dalong Sun

**Affiliations:** ^1^ Department of Gastroenterology and Hepatology, Xiamen Branch, Zhongshan Hospital, Fudan University, Xiamen, China; ^2^ National Health Commission (NHC) Key Laboratory of Glycoconjugates Research, Department of Biochemistry and Molecular Biology, School of Basic Medical Sciences, Fudan University, Shanghai, China; ^3^ Department of Neurology, Zhongshan Hospital, Fudan University, Shanghai, China; ^4^ Department of Gastroenterology and Hepatology, Zhongshan Hospital, Fudan University, Shanghai, China; ^5^ Shanghai Institute of Liver Disease, Shanghai, China

**Keywords:** H2A family member Z, prognosis, TP53, immune infiltration, hepatocellular carcinoma

## Abstract

H2A family member Z (H2AFZ) is a highly conserved gene encoding H2A.Z.1, an isoform of histone variant H2A.Z, and is implicated in cancer. In this study, we report that overexpression of H2AFZ is associated with tumor malignancy and poor prognosis in HCC patients. Functional network analysis suggested that H2AFZ mainly regulates cell cycle signaling and DNA replication *via* pathways involving several cancer-related kinases and transcription factor E2F1. Further studies revealed that H2AFZ overexpression is regulated by TP53 mutation and led to an attenuation of rapid proliferation phenotype and aggressive behavior in HCC cells. Moreover, we found that H2AFZ was related to immune infiltrations and was co-expressed with immune checkpoint genes, including CD274 (PD-L1), CTLA-4, HAVCR2 (TIM3), LAG3, PDCD1 (PD-1), and TIGIT (VSIG9) in HCC, indicating that H2AFZ-overexpressed HCC patients may be sensitive to immune checkpoint blockades (ICBs). Integrated analysis suggested that H2AFZ^high^/TP53^mut^ patients had the shortest OS and PFS time, but most likely to respond to ICBs. These findings indicate that the H2AFZ possesses potential value as a novel prognostic indicator for HCC patients and is correlated with immune infiltration in HCC, laying a foundation for future study of HCC investigation and intervention.

## Introduction

Hepatocellular carcinoma (HCC) is the most common form of liver cancer (1), with an incidence of approximately 850,000 new cases per year and represents the second leading cause of cancer-related deaths globally (2). The high recurrence and metastasis rate results in the poor 5-year survival rate for advanced liver cancer. However, due to the combination of factors spanning a series of different clinical and biological behaviors and the development of resistance to anti-HCC drugs, existing targeted drugs show unsatisfactory efficacy (3). The molecular mechanisms of tumor formation and progression remain to be revealed, which further complicates the effective treatment of HCC (4). In addition, the lack of specific markers for tumor types or disease stages represents a key gap in the current understanding and treatment of HCC.

Histones and histone variants, as basic components of nucleosome, are essential to chromatin structure and function in eukaryotes (5). H2A.Z.1 and H2A.Z.2 are two isotypes of histone variant H2A.Z respectively encoded by non-allelic genes H2A family member Z (H2AFZ) and H2A family member V (H2AFV) (6, 7). Overexpression of H2AFZ was subsequently detected in various malignant tumors, including prostate cancer (8, 9), bladder cancer (10), non-small cellular lung cancer (11), and breast cancer (12). In previous studies, overexpression of H2AFZ was indicated to promote tumor progression by regulating cell cycle transition (13, 14), reducing cell apoptosis (14), and promoting epithelial–mesenchymal transition (EMT) (15). However, the biological role of H2AFZ in HCC remains unclear. Thus, we performed multiple-dimensional bioinformatic analysis on data accessed from public databases to give insights into H2AFZ biological role in HCC and its value in HCC prognosis and treatment. Our results suggested that H2AFZ expression may be regulated by TP53 mutation, a frequently observed event in HCC, and is correlated to immune infiltrations in HCC.

In the current study, we performed multiple dimensional bioinformatic analysis to identify the role of H2AFZ in HCC and carried out a series of experiments demonstrating that H2AFZ overexpression is regulated by TP53 mutation and promotes the proliferation, migration, and invasion capabilities of HCC cells *in vitro*. H2AFZ is frequently overexpressed in HCC and associates with poor prognosis in patients with HCC. Analysis on differential expression genes (DEG) of H2AFZ^high^ and H2AFZ^low^ group suggested that H2AFZ mainly promotes cell proliferation and associates with resistance to platinum drugs. H2AFZ overexpression in HCC is mainly constituted by transcriptional factor E2F1 and is associated with a network of kinases including PLK1, CDK1, CDK2, AURKA, AURKB, and CHEK1. Further studies revealed that H2AFZ overexpression is related to TP53 mutation and immune infiltrations in HCC. Expression level of immune-checkpoint-relevant transcripts CD274 (PD-L1), CTLA-4, HAVCR2 (TIM3), LAG3, PDCD1 (PD-1), and TIGIT (VSIG9) were significantly higher in H2AFZ^high^ HCC patients, indicating that the H2AFZ^high^ HCC patients may be sensitive to immune checkpoint blockades (ICBs). Combined analysis of H2AFZ expression and TP53 status suggested that H2AFZ^high^/TP53^mut^ HCC patients had the worst prognosis and the greatest risk of tumor progression, while they were most likely to be sensitive to ICBs. Our results further revealed the biological role and potential clinical value of H2AFZ in HCC, laying a foundation for future study of HCC investigation and intervention.

## Results

### H2AFZ Is Overexpressed and Relates to Pathological Features in HCC

**Figure 1:**
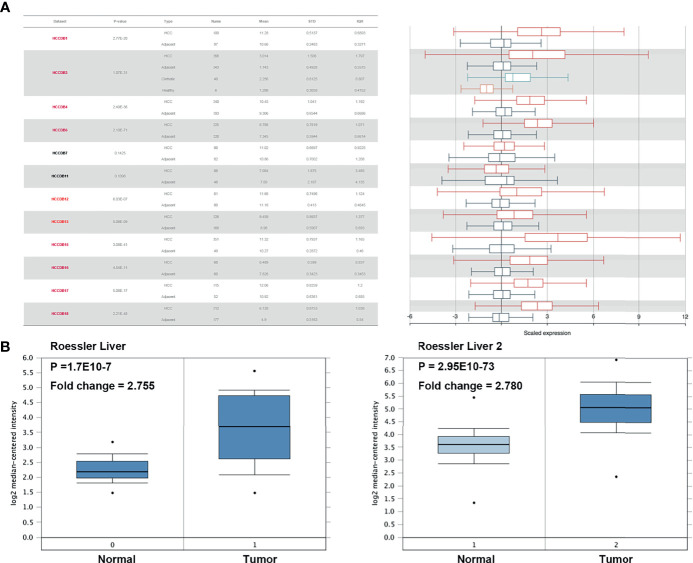
H2AFZ transcription level in HCC. **(A)** Chart and plot showing the expression of H2AFZ in tumor tissues and adjacent normal tissues in HCCDB. **(B)** Box plot showing H2AFZ mRNA levels in the Roessler Liver and Roessler Liver 2, respectively.

We initially evaluated H2AFZ expression in multiple HCC studies from TCGA and GEO. Analysis of 10 cohorts in the HCCDB database revealed that H2AFZ mRNA level was significantly higher in HCC tissues than in normal tissues (**Figure 1A**). H2AFZ mRNA levels in Roessler Liver and Roessler Liver2 reconfirmed overexpression of H2AFZ in HCC (**Figure 1B**).

**Figure 2:**
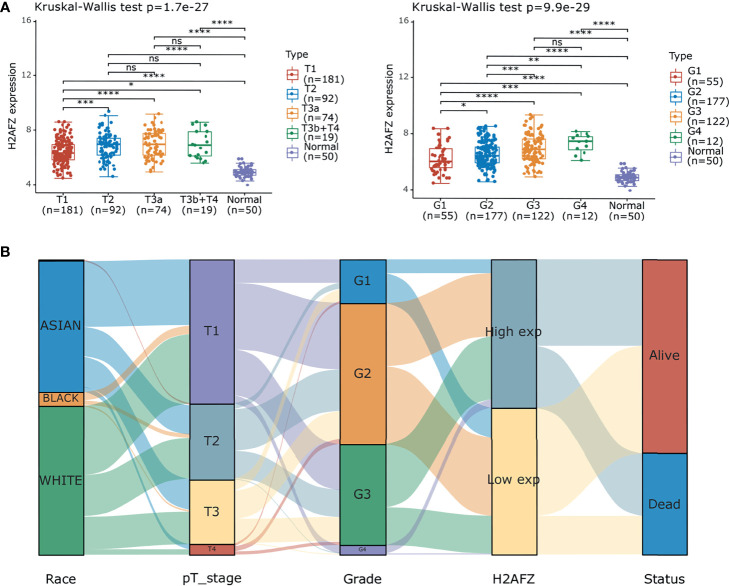
Correlation of H2AFZ and clinical-pathological features of HCC. **(A)** Box-dot plots showing the expression of H2AFZ in subgroups of TCGA-LIHC samples. **(B)** Sankey diagram presenting the distribution trend of H2AFZ on clinical–pathological features and survival outcomes of TCGA-LIHC patients. Mann-Whitney tests, *p < 0.05; **p < 0.001; ***p < 0.0001; ****p < 0.00001; ns, not significant.

Further subgroup analysis of multiple clinical–pathological features of TCGA-LIHC samples constantly showed elevated transcription level of H2AFZ, which is associated to pathological T stage and tumor grades of HCC (**Figure 2A**). Sankey diagram in **Figure 2B** represents the effect of H2AFZ expression on distribution trends of clinical features and survival outcomes.

### H2AFZ Overexpression Is Associated With Poor Prognosis in HCC

**Figure 3:**
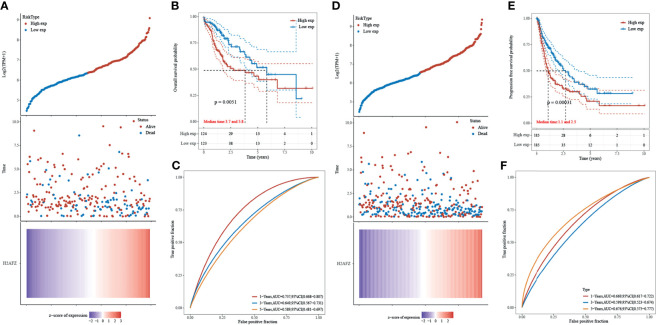
Prognostic analysis of H2AFZ in the TCGA-LIHC cohort. **(A)** The curve of risk score; the dotted line represented the median risk score and divided the patients into low-risk and high-risk group. Overall survival (OS) status of the patients; more dead patients corresponding to the higher risk score. Heatmap of the expression profiles of H2AFZ in the low- and high-risk group. **(B)** Kaplan–Meier overall survival analysis of H2AFZ in the TCGA-LIHC cohort. **(C)** Time-dependent ROC curves of OS of H2AFZ in the TCGA-LIHC cohort. **(D)** The curve of risk score. Progression-free survival (PFS) status of the patients. Heatmap of the expression profiles of H2AFZ in the low- and high-risk group. **(E)** Kaplan–Meier progression-free survival analysis of H2AFZ in the TCGA-LIHC cohort. **(F)** Time-dependent ROC curves of PFS of H2AFZ in the TCGA-LIHC cohort.

Kaplan–Meier curves were plotted to assess the association between H2AFZ expression and the survival outcomes of HCC. A total of 371 TCGA-LIHC patients were separated into two groups according to the median value of H2AFZ expression. H2AFZ expression level and survival outcomes, including overall survival (OS) and progression-free survival (PFS) time, are shown in **Figures 3A, D**. The high H2AFZ expression group had significantly shorter OS (log-rank test, *p* < 0.05, **Figure 3B**) and PFS (log-rank test, *p* < 0.05, **Figure 3E**) time, compared to the low expression group. Time-dependent ROC analysis represented prognostic capacity of H2AFZ expression in HCC (**Figures 3C, F**).

**Figure 4:**
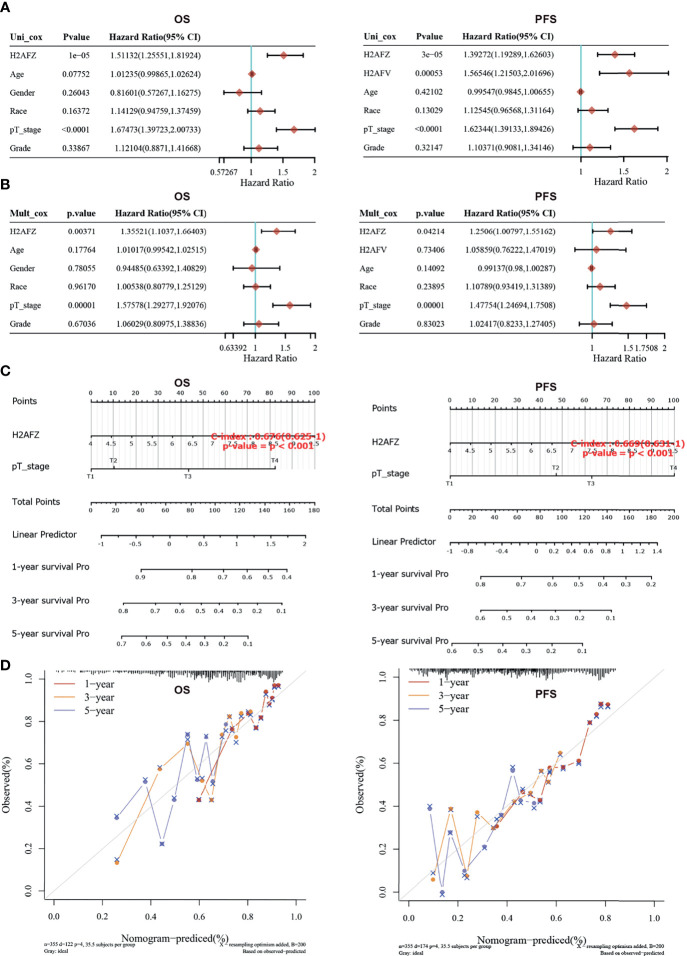
Construction and validation of the nomogram model. **(A)** Hazard ratios and *p*-values of constituents involved in univariate Cox regression. **(B)** Hazard ratios and *p*-values of constituents involved in multivariate Cox regression. **(C)** Nomograms to predict the 1-year, 2-year, and 3-year overall survival and progression-free survival of HCC patients. **(D)** Calibration curves for the overall survival nomogram model and the progression-free survival model.

Univariate and multivariate Cox regression analysis was performed to identify the proper terms to build the nomogram (**Figures 4A, B**). Nomogram involving H2AFZ expression level and pathological T stage predicts the 1-year, 2-year, and 3-year OS (C-index = 0.676, *p* < 0.001) and PFS of HCC patients (C-index = 0.669, *p* < 0.001, **Figure 4C**). Calibration curves of the nomogram models is shown in **Figure 4D**. These results indicate that H2AFZ is a potential prognostic biomarker in HCC.

### Identification of H2AFZ Biological Function in HCC

**Figure 5:**
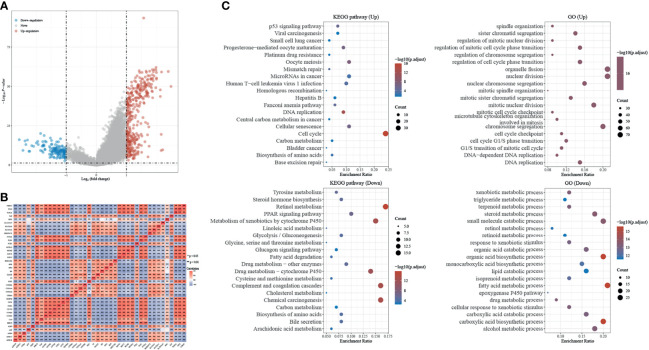
Identification and GSEA analysis of DEGs in H2AFZ^high^ and H2AFZ^low^ group. **(A)** Volcano plot showing DEGs in H2AFZ^high^ and H2AFZ^low^ group (|log2FC| > 2, *p* < 0.05) in TCGA-LIHC cohort. *p*-values were adjusted. **(B)** Spearman correlation analysis of top 20 upregulated genes and top 20 downregulated genes. **(C)** Significantly enriched GO annotations and KEGG pathways of DEGs.

Differential expression genes (DEGs) of H2AFZ^high^ and H2AFZ^low^ group were identified by “limma” (fold change > 2, FDR < 0.05). Volcano plot showed the 371 significantly upregulated and 135 significantly downregulated genes in the high H2AFZ expression group (**Figure 5A**). We represented correlated expression of top 20 upregulated and top 20 downregulated genes in a heat map (**Figure 5B**). A total description of DEGs is detailed to **Supplementary Table 1**.

Significant Gene Ontology (GO) term annotation and Kyoto Encyclopedia of Genes and Genomes (KEGG) pathway analysis by gene set enrichment analyze (GSEA) were performed on these genes. GO term annotation showed that genes involved in cell division and chromosome segregation were significantly upregulated, and KEGG pathway analysis showed that the cell cycle pathway was significantly activated, indicating that H2AFZ plays a critical role in tumor proliferation in HCC (**Figure 5C**). Moreover, significant enrichment was found in the platinum drug resistance pathway and base excision repair pathway (**Figure 5C**). DNA damage repair is the major process that mediates resistance to chemotherapy and radiotherapy, and correlated expression of H2AFZ and DNA-damage-repair-related transcripts is shown in **Supplementary Figure 2**. Further studies need to be performed to further confirm whether H2AFZ plays a key role in resistance to drugs in HCC.

**Figure 6:**
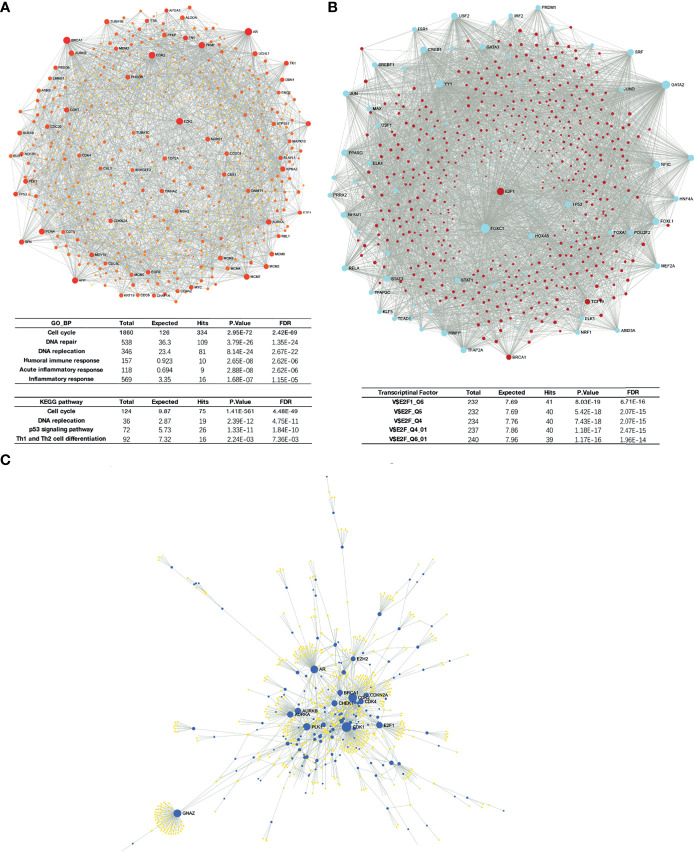
Network analysis of DEGs. **(A)** Protein–protein interaction (PPI) network of DEGs. **(B)** TF-gene network of DEGs. **(C)** Signaling network of DEGs.

Liver-specific protein–protein interaction (PPI) network of these differential expressed genes were built using NetworkAnalyst (**Figure 6A**). Significant enrichment in cell cycle and DNA replication process was found, as predicted. Moreover, GSEA results also suggested that H2AFZ may relate to immune response in HCC, and may serve as a regulator of Th1/Th2 cell differentiation (**Figure 6A** and **Supplementary Tables 3**, **4**). We will discuss the relationship between H2AFZ and immune infiltrations in HCC in the following context.

### Regulators of H2AFZ Expression in HCC

We initially investigated mutations and copy number alterations of H2AFZ in the TCGA-LIHC cohort using c-Bioportal and no alteration of H2AFZ copy number was found (**Supplementary Figure 1**), indicating that increased H2AFZ mRNA in HCC was not associated with the alteration of H2AFZ copy number. To further explore the regulators of H2AFZ in HCC, we analyzed the TF-gene network and signaling network of these genes. TF-gene analysis suggested that transcription factor E2F1, BRCA1, and TCF19 were associated with the network, and were overexpressed in the H2AFZ^high^ group. The enrichment of transcription factors was related mainly to the E2F transcription factor family (**Figure 6B** and **Supplementary Table 5**). Signaling network suggested that the polo like kinase 1 (PLK1), cyclin-dependent kinase 1 (CDK1), cyclin-dependent kinase 2 (CDK2), Aurora kinase B (AURKB), and checkpoint kinase 1 (CHEK1) were related to H2AZF overexpression (**Figure 6C**; **Table 1** and **Supplementary Table 4**). CHEK1, as downstream effector of ATR serine kinase, responds to DNA damage and mediates resistance to chemotherapy across cancer types (16). These kinases are all involved in the p53 signaling pathway as upstream or downstream molecules of p53 protein and regulates survival outcomes of HCC (**Supplementary Figure 3**). All these results indicated that TP53 may play an important role in H2AFZ-related networks in HCC, while no significant differential expression of TP53 was found in the H2AFZ^high^ group and H2AFZ^low^ group. Thus, we hypothesized that H2AFZ expression may associate with TP53 mutation, a frequently observed event in cancers. To demonstrate our hypothesis, we explored the correlation of H2AFZ expression and multiple mutations in HCC.

**Table 1:** Relative expression of H2AFZ in mutants and wild types of TP53, TTN, CTNNB1, MUC16, ALB, PCLO, RYR2, MUC4, ABCA13, and APOB.

Gene	Mutant		Wild type		*p*-value of Mann–Whitney test
	*n*	Mean of H2AFZ level (log10TPM)	*n*	Mean of H2AFZ level (log10TPM)	
TP53	105	7.051	254	6.435	****
TTN	124	6.611	235	6.617	ns
CTNNB1	88	6.501	271	6.652	ns
MUC16	79	6.743	280	6.579	ns
ALB	40	6.502	319	6.629	ns
PCLO	50	6.544	309	6.627	ns
RYR2	46	6.803	313	6.588	ns
MUC4	48	6.618	311	6.615	ns
ABCA13	42	6.691	317	6.605	ns
APOB	38	6.621	321	6.615	ns

****p < 0.00001; ns, not significant.

### TP53 Mutation Relates to H2AFZ Expression in HCC

**Figure 7:**
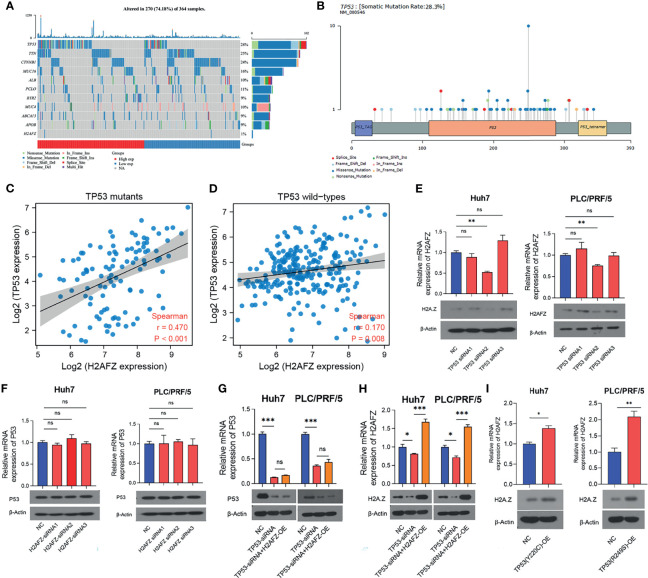
H2AFZ expression was regulated by TP53 mutation in HCC. **(A)** Lollipop plot displaying mutation distribution and protein domains for TP53 in HCC with the labeled recurrent hotspots. Somatic mutation rate and transcript names were indicated by plot title and subtitle, respectively. **(B)** Oncoplot displaying the somatic landscape of 270 patients in the TCGA-LIHC cohort. Genes were ordered by their mutation frequency, and samples were ordered according to their H2AFZ expression. Side bar plot showed log10 transformed *Q*-values estimated by MutSigCV. Mutation information of each gene in each sample was shown in the waterfall plot, where different colors with specific annotations at the bottom meant the various mutation types. **(C, D)** Co-expression of H2AFZ and TP53 in TP53 wild types **(C)** and TP53 mutants **(D)** in the TCGA-LIHC cohort. **(E, F)** Relative mRNA and protein expression level of H2AFZ in non-targeting control (NC)/TP53-siRNA1/TP53-siRNA2/TP53-siRNA3 Huh7 cells **(E)** and PLC/PRF/5 cells **(F)**. **(G, H)** Relative mRNA and protein expression level of TP53 in non-targeting control (NC)/H2AFZ-siRNA1/H2AFZ-siRNA2/H2AFZ-siRNA3 Huh7 cells **(G)** and PLC/PRF/5 cells **(H)**. **(I)** Relative mRNA and protein expression level of H2AFZ in NC/TP53-OE Huh7 and PLC/PRF/5 cells. *T*-tests (*n* = 3), *p < 0.05, **p < 0.001, ***p < 0.0001; ns, not significant.

Tumor suppressor protein p53 responds to diverse cellular stresses to regulate expression of target genes, thereby inducing cell cycle arrest, apoptosis, senescence, DNA repair, or changes in metabolism (17). TP53 mutations are universal across cancer types, which leads to loss of p53 function (18). Here, we showed somatic landscape of single-nucleotide variants (SNVs) in the TCGA-LIHC cohort (**Figures 7A, B**), and the samples were sorted by their H2AFZ expression in **Figure 7A**. H2AFZ expression level in mutants and wild types of these genes were compared by Mann–Whitney tests. As shown in **Table 1**, H2AFZ expression was significantly higher in TP53 mutants, compared to TP53 wild types, while showing no significant difference in variants compared to wild types of other common mutant genes. Moreover, we found that TP53 showed a moderate co-expression with H2AFZ (*r* = 0.47, *p* < 0.001, Spearman test) in TP53 mutants (*n* = 105, **Figure 7C**), while showing a weak co-expression with H2AFZ (*r* = 0.17, *p* = 0.008, Spearman test) in TP53 wild types (*n* = 254, **Figure 7D**). These results indicate that TP53 mutation may act as an upstream regulator of H2AFZ expression.

Huh7 and PLC/PRF/5 are TP53-mutated HCC cell lines with Y220C-mutant p53 and R249S-mutant p53, respectively. To further investigate the regulatory role of TP53 mutation in H2AFZ expression, we initially built 3 TP53-siRNA and 3 H2AFZ-siRNA, and tested their interference efficiency in Huh7 and PLC/PRF/5 cell lines, respectively. TP53-siRNA2 and H2AFZ-siRNA2 showed most significant reduction in mRNA and protein expression levels of TP53 and H2AFZ (**Supplementary Figures 4A**, **4B**). As the expression level of TP53 was lowered, the expression level of H2AFZ in Huh7 and PLC/PRF/5 (**Figure 7E**) also decreased significantly. The expression level of TP53 showed no significant alteration as the expression level of H2AFZ was reduced (**Figure 7F**). TP53-siRNA2 was then transitioned into NC/H2AFZ-OE Huh7 and PLC/PRF/5 cell lines, respectively. Both expression levels of TP53 (**Figure 7G**) and H2AFZ (**Figure 7H**) can be significantly decreased by TP53-siRNA2, and overexpressing H2AFZ could not significantly alter the expression level of TP53 (**Figure 7G**). Moreover, TP53 (Y220C) and TP53 (R249S) overexpression plasmids were constructed, transfected into Huh7 and PLC/PRF/5 cells *via* lentiviral, respectively. Both expression levels of TP53 and H2AFZ were significantly elevated after the transfection (**Figure 7I**).

### H2AFZ Overexpression Promotes HCC Cell Proliferation, Migration, and Invasion *In Vitro*

**Figure 8:**
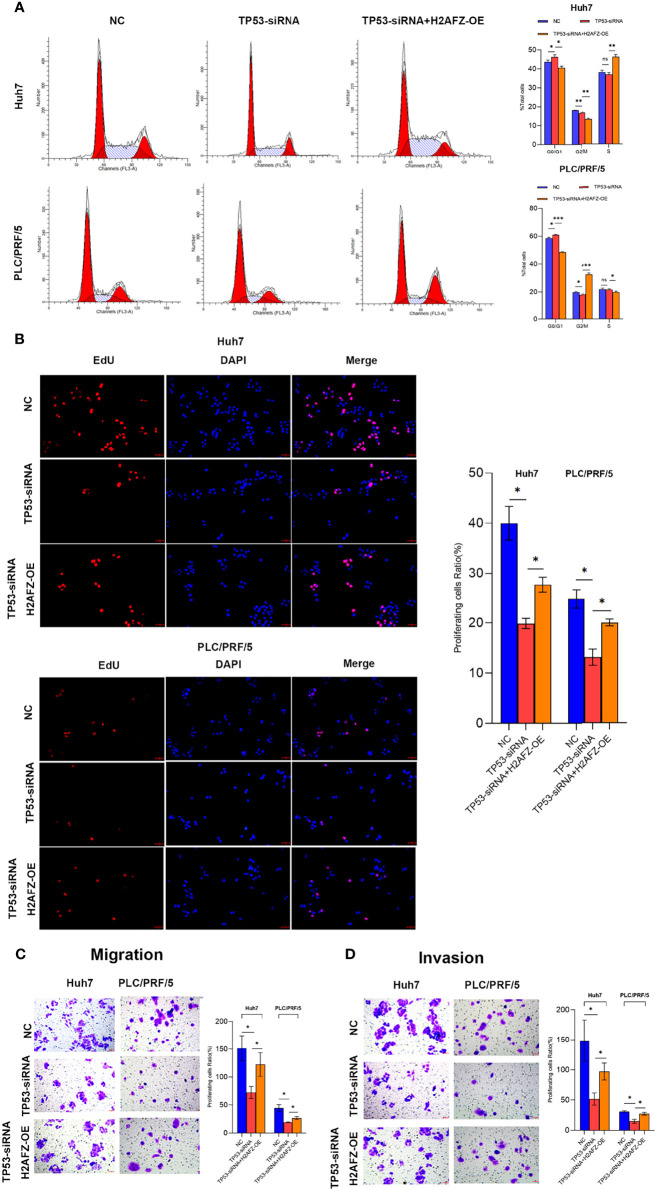
H2AFZ overexpression enhances proliferation, migration and invasion capability of HCC cells *in vitro*. (A–D) Flow cytometry (FCM) assay **(A)**, 5-Ethynyl-2’-deoxyuridine (EdU) proliferation assay (bar = 50 μm, ×20) **(B)**, transwell migration assay **(C)**, and Matrigel-transwell invasion assay **(D)** on NC/TP53-siRNA/TP53-siRNA+H2AFZ-OE Huh7 and PLC/PRF/5 cells. *T*-tests (*n* = 3), *p < 0.05, **p < 0.001, ***p < 0.0001 ns, not significant.

To explore the effect of H2AFZ overexpression on the biological behavior of HCC cells, we performed flow cytometry (FCM), 5-Ethynyl-2’-deoxyuridine (EdU) proliferation assay, transwell migration assay, and Matrigel-transwell invasion assay on NC/TP53-siRNA/TP53-siRNA+H2AFZ-OE Huh7 and PLC/PRF/5 cells, respectively. Compared with NC cells, lower expression of TP53 led to increased percentage of cells in the G0/G1 phase and decreased percentage of cells in the G2/M phase; supplementing H2AFZ led to decreased percentage of cells in the G0/G1 phase and increased percentage of cells in the G2/M or S phase in both Huh7 and PLC/PRF/5 cell lines (**Figure 8A**). EdU proliferation assay showed that lower TP53 expression reduced cell proliferation capability, which could significantly rebound after supplementing H2AFZ in both Huh7 and PLC/PRF/5 cell lines (**Figure 8B**). Transwell migration assay and invasion assay showed that lowering TP53 expression decreased cell migration and invasion capability, while H2AFZ overexpression led to an attenuation of aggressive behavior (**Figures 8C, D**). All these results indicated that TP53 mutation and H2AFZ overexpression could enhance proliferation, migration, and invasion capability of HCC cells *in vitro*.

### H2AFZ Overexpression Regulates Immune Infiltration in HCC

**Figure 9:**
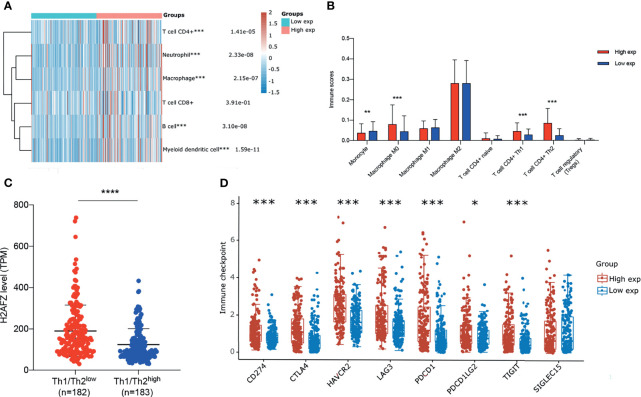
Correlation of H2AFZ and immune infiltrations in HCC. **(A)** The score distribution of CD4+ T cells, neutrophils, macrophages, CD8+ T cells, B cells, and myeloid dendritic cells in the H2AFZ^high^ group and H2AFZ^low^ group, estimated by TIMER. **(B)** Scores of monocytes, macrophage M0, macrophage M1, macrophage M2, naive CD4+ T cells, Th1 cells, Th2 cells, and Tregs in the H2AFZ^high^ group and the H2AFZ^low^ group, estimated by CIBERSORT and xCell (Mann–Whitney tests, *p < 0.05, **p < 0.001, ***p < 0.0001). **(C)** Differential H2AFZ expression level in Th1/Th2^high^ patients and Th1/Th2^low^ patients (Mann–Whitney test, *p* < 0.00001). **(D)** Expression levels of immune-checkpoint-relevant transcripts in the H2AFZ^high^ and H2AFZ^low^ group, respectively (Mann–Whitney tests, *p < 0.05, **p < 0.001, ***p < 0.0001).

We estimated immune infiltration status of TGCA-LIHC patients *via* RNAseq data using multiple algorithms, including CIBERSORT, TIMER, and xCell. Each algorithm has unique advantages in particular analysis. To identify the differential immune infiltration in the H2AFZ^high^ group and H2AFZ^low^ group, we initially compared immune cell infiltration scores estimated *via* TIMER of the two groups. As shown in **Figure 9A**, infiltration levels of CD4+ T cells, neutrophils, macrophages, B cells, and myeloid dendritic cells were significantly higher in the H2AFZ high expression group, compared to the H2AFZ low expression group. Thus, we further estimated infiltration scores of macrophage subtypes including M0, M1, and M2 using the CIBERSORT method and infiltration scores of CD4+ T cell subtypes including naive, Th1, and Th2 using the xCell method. As shown in **Figure 9B**, infiltration levels of macrophage M0, T cell CD4+ Th1, and T cell CD4+ Th2 were significantly upregulated in the H2AFZ^high^ group, indicating that the H2AFZ^high^ group may have a lower Th1/Th2 ratio, which is an event related to tumor immune evasion and poor prognosis of HCC. We divided 365 TCGA-LIHC patients into the Th1/Th2^low^ group and Th1/Th2^high^ group, according to the median value of Th1/Th2. Significantly higher H2AFZ expression level was found in the Th1/Th2^low^ group, compared to the Th1/Th2^high^ group (Mann–Whitney test, *p* < 0.00001, **Figure 9C**). Patients in the Th1/Th2^low^ group had significantly shorter OS and PFS time, compared to the Th1/Th2^high^ group (**Supplementary Figure 4**), reconfirming lower Th1/Th2 ratio associates with poor survival outcomes of HCC. Moreover, liver-specific PPI network analysis suggested that Th1 and Th2 differentiation pathway was significantly activated along with H2AFZ overexpression, as mentioned in the above. All these results suggested that H2AFZ may play an important role in T-cell differentiation regulating.

Immune checkpoints are a series of molecules that mediate immune evasion, which can be blocked by ICBs (19, 20). CD274, CTLA4, HAVCR2, LAG3, PDCD1, PDCD1LG2, TIGIT, and SIGLEC15 were selected to be immune-checkpoint-relevant transcripts and the expression values of these eight genes were extracted. Significantly higher CD274, CTLA4, HAVCR2, LAG3, PDCD1, PDCD1LG2, and TIGIT were found in the H2AFZ^high^ group, compared to the H2AFZ^low^ group (**Figure 9D**). However, because immunotherapy has not been widely applied in HCC clinical treatment, more evidence is required to demonstrate H2AFZ predicting capacity on sensitiveness to ICBs of HCC patients.

### Combined Analysis of H2AFZ Expression and TP53 Status Improves Prognostic Value of HCC Patient Outcome

**Figure 10:**
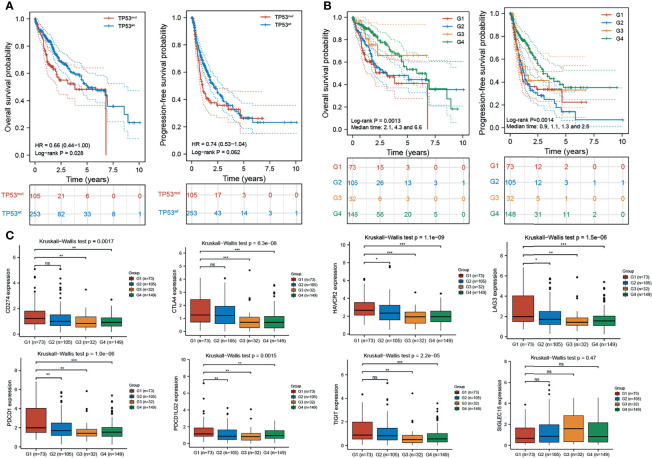
Combined analysis of H2AFZ expression and TP53 status improves prognostic value of HCC patient outcome. **(A)** Overall survival and progression-free survival of TP53^mut^ and TP53^wt^ patients. **(B)** Overall survival and progression-free survival of the four subgroups (G1: H2AFZ^high^/TP53^mut^; G2: H2AFZ^high^/TP53^wt^; G3: H2AFZ^low^/TP53^mut^; G4: H2AFZ^low^/TP53^wt^). **(C)** Expression levels of immune-checkpoint-relevant transcripts in the four subgroups, respectively *p < 0.05; **p < 0.001; ***p < 0.0001; ns, not significant.

Integrated analysis of H2AFZ expression and TP53 status provided a more powerful prediction for HCC patient outcomes. As shown in **Figure 10A**, TP53^mut^ patients had shorter OS (log-rank test, *p* < 0.05) and PFS (log-rank test, *p* = 0.062) time compared to TP53^wt^ patients. Patients were classified into four subgroups based on H2AFZ expression and TP53 status (G1: H2AFZ^high^/TP53^mut^, G2: H2AFZ^high^/TP53^wt^, G3: H2AFZ^low^/TP53^mut^, and G4: H2AFZ^low^/TP53^wt^). Subgroup comparisons showed that patients in the H2AFZ^high^/TP53^mut^ group had the worst prognosis and the greatest risk of tumor progression. Conversely, HCC patients in the H2AFZ^low^/TP53^wt^ group had the best prognosis (**Figure 10B**). The median OS of Groups 1, 2, and 4 were 2.1, 4.3, and 6.6 years (*p* < 0.001), respectively. While the median PFS of Groups 1, 2, 3, and 4 were 0.9, 1.1, 1.3, and 2.5 years (*p* = 0.002), respectively. Taken together, these results evidently indicated that integrated analysis of H2AFZ expression and TP53 status could serve as a more powerful predictor of prognosis in HCC patients. Expression levels of the immune-checkpoint-relevant transcripts of the four subgroups are shown in **Figure 10C**. Expression levels of HAVCR2, LAG3, and PDCD1LG2 were significantly higher in the H2AFZ^high^/TP53^mut^ group, compared to the other three groups (Mann–Whitney tests, *p < 0.05, **p < 0.001, ***p < 0.0001)), indicating that H2AFZ^high^/TP53^mut^ patients may be the most sensitive to ICBs among the four subgroups. Further clinical evidence is needed to demonstrate our hypothesis.

## Discussion

The lack of specific markers for tumor types or disease stages represents a key gap in the current understanding and treatment of HCC. In the current study, we reported a histone variant, H2AFZ, whose overexpression is associated with poor prognosis of HCC. Also, H2AFZ overexpression relates to multiple clinical–pathological features including pathological T stage and tumor grade of HCC. To gain more detailed insights into the potential functions of H2AFZ in HCC, we performed multiple bioinformatics analysis of public data.

Analysis of transcriptome from 12 HCC cohorts demonstrated that H2AFZ mRNA level is significantly higher in HCC than in normal liver tissue. In addition, high expression of H2AFZ was significantly related to poor survival and progression-free state. Thus, our results suggest that H2AFZ upregulation occurs in most cases of HCC and deserves further clinical validation as a potential diagnostic and prognostic marker.

To explore the biological function of H2AFZ and probe the signaling events in controlling abnormal H2AFZ expression, we tested the networks of DEGs. GSEA analysis suggested that the functional consequence of H2AFZ mainly acts in cell cycle process and promotes cell proliferation. Moreover, significant enrichment was found in platinum drug resistance pathway. Further studies need to be done to investigate whether H2AFZ mediates resistance to chemotherapy critically in HCC.

For mining regulators potentially responsible for H2AFZ overexpression, we found that the E2F family constitute the main transcription factors for H2AFZ overexpression. E2F1 is one of the key links in the cell cycle regulation network. Activated E2F oncogenic signaling was frequently observed in the progression of liver cancer (21). Our results suggest that E2F1 is an important regulator of H2AFZ and that H2AFZ might act through this factor to regulate the cell cycle and proliferation capacity of HCC. Further studies are needed to test this hypothesis.

Next, H2AFZ in HCC is associated with a network of kinases including PLK1, CDK1, CDK2, AURKA, AURKB, and CHEK1. These kinases regulate genomic stability, mitosis, and the cell cycle, and showed differential expression and survival prognosis in HCC. CHEK1, the major downstream effector of ATR kinase, responds to DNA damage and thus mediates resistance to radiotherapy and chemotherapy. Activation of the ATR–CHK1 pathway suggested that the H2AFZ^high^ patients are less likely to be sensitive to radiotherapy and chemotherapy (22), which need to be further demonstrated. Moreover, these kinases are all involved in the p53 signaling pathway as regulators or effectors of p53 protein (23); thus, we discussed the relation between TP53 mutation and H2AFZ expression in the following context.

Tissue-specific PPI network analysis further demonstrates that H2AFZ regulates cell cycle signaling and DNA replication critically in HCC. Moreover, significant enrichment was found in the p53 signaling pathway and Th1/Th2 cell differentiation pathway. Thus, we further explored the association between H2AFZ expression and TP53 mutation and H2AFZ’s regulatory role in tumor immune in HCC.

We screened the top 10 most frequently mutated genes in the TCGA-LIHC cohort, and H2AFZ expression levels between mutants and wild types of these genes were then compared. Significantly higher H2FAZ expression level was found in TP53 mutants, compared to TP53 wild types, while no significant differential H2AFZ expression was found in mutants and wild types of other genes. Moreover, TP53 was found significantly co-expressed with H2AFZ in TP53 mutants, suggesting that TP53 mutation might be a regulatory event of H2AFZ expression in HCC.

Thus, we performed multiple *in vitro* experiments on Huh7 (TP53 Y220C mutant) and PLC/PRF/5 (TP53 R249S mutant) HCC cell lines. We demonstrated that H2AFZ expression was positively regulated by TP53, while TP53 expression was not altered by H2AFZ in Huh7 and PLC/PRF/5 cell lines, indicating that TP53 mutant is an upstream regulator of H2AFZ in HCC.

The tumor microenvironment is the non-cancerous cells present in or around tumors (24) and immune infiltration is a prognostic feature in cancers (25). For mining the relationship between H2AFZ and diverse immune infiltrations in LIHC, we performed multiple algorithms on RNA-seq data of TCGA-LIHC. Significantly higher CD4+ T cell, B cell, neutrophil, macrophage, and myeloid dendritic cell infiltration level was found in the H2AFZ^high^ group, compared to the H2AFZ^low^ group. Further studies revealed that infiltration levels of macrophage M0, CD4+ Th1, and CD4+ Th2 were significantly upregulated along with H2AFZ overexpression. Moreover, lower Th1/Th2 ratio was found in the H2AFZ^high^ group, which is an event related to immune deficiency and poor prognosis of HCC. Th2 cells could promote tumor immune evasion, thereby promoting tumor occurrence, development, and resistance to drugs (26). PPI network analysis of H2AFZ co-expressed genes suggested significant enrichment in the Th1/Th2 differentiation pathway, as mentioned formerly. These results further demonstrate that H2AFZ may play an important role in T-cell differentiation regulating.

Immune checkpoints are a series of molecules that mediate immune evasion of tumors and can be therapeutically targeted by ICBs (19, 20). Significant higher expression level of immune checkpoint genes including CD274, CTLA4, HAVCR2, LAG3, PDCD1, and TIGIT was found in the H2AFZ^high^ group, suggesting that H2AFZ overexpressed HCC patients are more likely to be sensitive to ICBs. Our result could potentially provide guidance for its clinical application. However, because ICBs have not been widely applied in HCC treatment currently, more clinical evidence is needed to demonstrate our hypothesis.

Integrated analysis of H2AFZ expression and TP53 status provided a more powerful prediction for HCC patient outcomes. Our results showed that H2AFZ^high^/TP53^mut^ HCC patients had the worst prognosis and the greatest risk of tumor progression while H2AFZ^low^/TP53^wt^ HCC patients had the best prognosis. Moreover, significantly higher HAVCR2, LAG3, and PDCD1LG2 expression level were found in the H2AFZ^high^/TP53^mut^ subgroup compared to other three subgroups, indicating that H2AFZ^high^/TP53^mut^ patients are most likely to be sensitive to ICBs.

In conclusion, the present study demonstrated that H2AFZ overexpression is regulated by TP53 mutation and promotes tumor occurrence and progression in HCC. Our results further revealed that H2AFZ regulates immune infiltration and Th1/Th2 cell differentiation. The H2AFZ^low^/TP53^wt^ group representing low immune checkpoint expression predicts a favorable outcome, while the H2AFZ^high^/TP53^mut^ group implying high immune checkpoint expression status suggests poor prognosis in HCC patients, respectively. Combined analysis of H2AFZ expression and TP53 status provided a better prognostic value for HCC survival and progression. The H2AFZ^high^ patients are less likely to be sensitive to radiotherapy and chemotherapy, while they are more likely to be sensitive to ICBs. Thus, our findings provided a potential target for future investigation and intervention of HCC.

## Materials and Methods

### Data Sources

For the LIHC patients of The Cancer Genome Atlas (TCGA) database, gene expression, clinical, and somatic mutation data were downloaded from the Genomic Data Commons (GDC) data portal (TCGA) (https://portal.gdc.cancer.gov/) and 50 of the tumors also had mRNA expression data of paired normal tissue samples.

### Gene Expression Correlation Analysis

The multi-gene correlation map was displayed by the R software package “pheatmap”. We used Spearman’s correlation analysis to describe the correlation between quantitative variables without a normal distribution. A *p*-value of less than 0.05 was considered statistically significant.

### Differential Expressed Gene Analysis and Gene Expression Comparison Analysis

“Limma” R package was used to identify the differentially expressed mRNAs in the H2AFZ^high^ group compared to the H2AFZ^low^ group. The adjusted *p*-value was analyzed to correct for false-positive results. Adjusted *p* < 0.05 and |Fold Change| >2 was defined as the thresholds for the screening of differential expression of mRNAs. “ClusterProfiler” R package was employed to analyze the GO functions of potential targets and enrich the Kyoto Encyclopedia of Genes and Genomes (KEGG) pathways.

### Gene Mutation Analysis

To identify the somatic mutations of the patients with LIHC in the TCGA database, mutation data were downloaded and visualized using the “maftools” package in R software.

### Survival Analysis of TCGA-LIHC Patients

The KM survival analysis with log-rank test was also used to compare the survival difference between the above two groups or more groups. TimeROC analysis was performed to compare the predictive accuracy of each gene and risk score. For Kaplan–Meier curves, *p*-values and hazard ratio (HR) with 95% confidence interval (CI) were generated by log-rank tests and univariate Cox proportional hazards regression. Risk curves were plotted using the “ggrisk” R package. KM curves were plotted using “survival” and “survminer” R packages. Time-dependent ROC curves were plotted using the “TimeROC” R package. *p*-value of <0.05 was considered statistically significant.

All the above analysis methods and R package were implemented by R foundation for statistical computing (2020) version 4.0.3.

### Construction and Validation of the Nomogram

Univariate and multivariate Cox regression analysis was performed to identify the proper terms to build the nomogram. The forest was used to show the *p*-value, HR, and 95% CI of each variable through “forestplot” R package. A nomogram was developed based on the results of multivariate Cox proportional hazards analysis to predict the 1-, 3-, and 5-year OS and PFS. Nomograms and calibration curves were plotted using the “rms” R package. Statistical analyses were performed using R software v4.0.3. *p*-value of <0.05 was considered statistically significant.

### Tumor Immune Microenvironment Analysis

To make reliable immune infiltration estimations, we utilized the “immunedeconv”, an R package that integrates six state-of-the-art algorithms, including TIMER, xCell, MCP-counter, CIBERSORT, EPIC, and quanTIseq. TIMER applies a deconvolution method to infer the abundance of tumor-infiltrating immune cells (TIICs) from gene expression profiles (27), and is used to estimate infiltration levels of B cells, CD4+ T cells, CD8+ T cells, neutrophils, macrophages, and dendritic cells. CIBERSORT performs deconvolution based on the ν-SVR method to infer the abundance of tumor-infiltrating immune cells (TIICs) from gene expression profiles (28), and is used for macrophage subtype infiltration level estimating. xCell is an algorithm that performs cell-type enrichment analysis from gene expression data for 64 immune and stroma cell types (29), and is used for CD4+ T-cell subtype and regulatory T-cell infiltration level estimating.

For grouping TCGA-LIHC patients into the Th1/Th2^low^ and Th1/Th2^high^ group, we initially wiped off 6 patients whose Th1 and Th2 score were both 0. Of 371 patients, 365 were then separated into the Th1/Th2^low^ group and Th1/Th2^high^ group, according to the median value of their Th1/Th2 ratio. Th1 and Th2 scores were decided by xCell outcomes.

All the above analysis methods and R packages were implemented by R software v4.0.3 (R Foundation for Statistical Computing, Vienna, Austria).

### Online Database Analysis

#### HCCDB Database Analysis

HCCDB (http://lifeome.net/database/hccdb/) is a database of HCC expression atlas containing data from the Gene Expression Omnibus (GEO), Liver Hepatocellular Carcinoma Project of The Cancer Genome Atlas (TCGA-LIHC), and Liver Cancer - RIKEN, JP Project from International Cancer Genome Consortium (ICGC LIRI-JP) (30). Differential H2AFZ mRNA level in HCC tissue and normal tissue was determined by HCCDB.

#### Oncomine Database Analysis

The expression level of the H2AFZ gene in HCC was examined in the Oncomine 4.5 database (https://www.oncomine.org/). The threshold was determined according to the following values: *p*-value of 0.05, fold change of 1.5, and gene ranking of all.

#### Network Analyst Database Analysis

Network interpreting gene expression analysis of H2AFZ DEGs was performed by NetworkAnalyst 3.0 tool (https://www.networkanalyst.ca), which integrates cell-type or tissue-specific PPI networks, gene regulatory networks, and gene co-expression networks (31). Function enrichment was based on a similar concept introduced by ClueGO and EnrichmentMap (32).

#### c-BioPortal Database Analysis

The cBio Cancer Genomics Portal (http://cbioportal.org) has multidimensional cancer genomics datasets (33). Mutation and copy number variation (CNV) of H2AFZ in HCC were analyzed using the c-BioPortal tool.

#### GEPIA Database Analysis

The Gene Expression Profiling Interactive Analysis (GEPIA) database (http://gepia.cancer-pku.cn/) (34) was used to generate survival curves, including OS and recurrence-free survival (RFS), based on gene expression with the log-rank test and the Mantel-Cox test in HCC.

### Experiments

#### Cell Lines

Huh7 and PLC/PRF/5 cell lines were purchased from the cell bank of FuHeng Biology (Shanghai, China). Cells were maintained in Dulbecco’s modified Eagle’s medium (DMEM, Invitrogen, Carlsbad, CA, USA) combined with 10% fetal bovine serum (FBS; Gibco, Grand Island, MA, USA). All cell lines were authenticated by short tandem repeat analysis during the study period.

#### Construction of TP53 and H2AFZ Interference and Overexpression Clones

A non-targeting control sequence, three short interfering RNA (siRNA) sequence targeting TP53, three siRNA sequence targeting H2AFZ, and full-length TP53 Y220C, TP53 R249S, and H2AFZ cDNA were cloned into the pHY-LV-OE1.7 (pHY-009, Hanyin Biotech, Shanghai, China). Cells at a confluence of 6% in six-well plates were infected by lentivirus, selected by 2 μg/ml puromycin for 5 days. The mRNA levels were detected by quantitative real-time polymerase chain reaction (qRT-PCR), and the protein levels were detected by Western blot.

#### Western Blot Analysis

Harvested cells were lysed in RIPA buffer (Beyotime, Shanghai, China). Protein concentrations were determined by BCA Protein Assay Kit (Pierce, Rockford, IL, USA). Forty micrograms of protein was resolved on 10% sodium dodecyl sulfate–polyacrylamide gel electrophoresis, transferred to polyvinylidene difluoride membranes, blocked with 5% non-fat dry milk for 1 h at room temperature, and then incubated with primary antibodies at 4°C overnight. Blots were detected by ECL reagent (Tiangen, Beijing, China).

#### Quantitative Polymerase Chain Reaction

Total RNA was extracted from harvested cells using TRIzol reagent (Invitrogen, Carlsbad, CA, USA) according to the manufacturer’s instructions. Total RNA was reversed to cDNA by PrimeScript RT Master Mix (Takara, Dalian, China) and mRNA levels were detected using SYBR Premix Ex Taq II (Takara) according to the manufacturer’s instructions. The primers synthesized by Hanyin Biotech (Shanghai, China) were as follows: TP53, forward 5’-CCTCAGCATCTTATCCGAGTGG-3’, reverse 5’-TGGATGGTGGTACAGTCAGAGC-3’; H2AFZ, forward 5’-GCAACTTGCTATTCGTGGAGATG-3’, reverse 5’-CAGGCATCCTTTAGACAGTCTTC-3’ and GAPDH forward, 5’-GTCTCCTCTGACTTCAACAGCG-3’, reverse 5’-ACCACCCTGTTGCTGTAGCCAA-3’. qPCR was performed with ABI 7500 (Applied Biosystem, Carlsbad, CA). Data were normalized for GAPDH levels by the ΔΔCt method.

#### Cell Function Assays *In Vitro*

The effect of TP53 mutation and H2AFZ overexpression on the cell cycle was analyzed by flow cytometry. Cells were trypsinized and fixed with ice-cold 70% ethanol at 4°C overnight and incubated with 50 μg/ml propidium iodide (Sigma-Aldrich) in the presence of 100 μg/ml RNase and 0.2% Triton X-100 for 30 min at 37°C. DNA content was determined in the BD FACSAria II system (BD Biosciences, Franklin Lakes, NJ, USA). We assessed cell proliferation using the Cell-Light EdU DNA Cell Proliferation Kit (C10310-1, RiboBio, Guangzhou, China) according to the manufacturer’s instructions. Transwell migration and invasion assay was undertaken in a chamber of 8-μm-pore Transwell inserts precoated with or without 100 μl Matrigel (BD Biosciences, San Jose, CA, USA). Cells (1 × 10^5^) were seeded in each insert. Cells that had migrated or invaded were fixed by 4% paraformaldehyde, stained with crystal violet for image capture, and counted under a light microscope.

Data are representative of three independent experiments carried out in triplicate.

## Data Availability Statement

The original contributions presented in the study are included in the article/**Supplementary Material**. Further inquiries can be directed to the corresponding authors.

## Author Contributions

DS and LD designed and conceived this project. MD, YD, and DZ developed methodology. DZ designed the experiments. MD, JC, and YD performed bioinformatics analysis and generated data. JC and YD analyzed and interpreted data and performed the experiments. MD wrote the draft. DS and LD revised and finalized the manuscript. All authors contributed to the article and approved the submitted version.

## Funding

This work was supported by grants from the National Natural Science Foundation of China (81972234), Medical and Health Science and Technology Project of Xiamen (3502Z20194032), and Young and Middle-aged Backbone Training Project in the Health System of Fujian Province (2020GGB060).

## Conflict of Interest

The authors declare that the research was conducted in the absence of any commercial or financial relationships that could be construed as a potential conflict of interest.

## Publisher’s Note

All claims expressed in this article are solely those of the authors and do not necessarily represent those of their affiliated organizations, or those of the publisher, the editors and the reviewers. Any product that may be evaluated in this article, or claim that may be made by its manufacturer, is not guaranteed or endorsed by the publisher.
