# Supplementary material for: H2AFZ Is a Prognostic Biomarker Correlated to TP53 Mutation and Immune Infiltration in Hepatocellular Carcinoma
Source: Front Oncol. 2021 Oct 25;11:701736. doi: 10.3389/fonc.2021.701736 (PMC8573175; doi:10.3389/fonc.2021.701736)
Supplement: Supplementary file 1 [file DataSheet_1.docx]

Supplementary Material

**List of supporting information**

Materials involved in this study

**List of materials involved in this study**

| **siRNAs** | **sequence** |
| --- | --- |
| H2AFZ siRNA1 | CCGTATTCATCGACACCTA |
| H2AFZ siRNA2 | CTTGCAACTTGCTATTCGT |
| H2AFZ siRNA3 | GGAAGAAAGGACAACAGAA |
| TP53 siRNA1 | GTACCACCATCCACTACAA |
| TP53 siRNA2 | AGAGAATCTCCGCAAGAAA |
| TP53 siRNA3 | GGAGTATTTGGATGACAGA |
| **primers** | **sequence** |
| Human-TP53-F | CCTCAGCATCTTATCCGAGTGG |
| Human-TP53-R | TGGATGGTGGTACAGTCAGAGC |
| Human-H2AFZ-F | GCAACTTGCTATTCGTGGAGATG |
| Human-H2AFZ-R | CAGGCATCCTTTAGACAGTCTTC |
| Human GAPDH-F | GTCTCCTCTGACTTCAACAGCG |
| Human GAPDH-R | ACCACCCTGTTGCTGTAGCCAA |

| **Antibodies** | **Company** | **Stock number** | **Dilution ratio** |
| --- | --- | --- | --- |
| P53 | santa | sc-126 | 1/500 |
| H2AZ | CST | 2718S | 1/500 |

| **Softwares** | **Version** |
| --- | --- |
| R | 4.0.3 |
| Graphpad prism | 9.2 |
| **R packages** | **Version** |
| ggplot2 | 3.3.5 |
| pheatmap | 1.0.12 |
| limma | 3.48.3 |
| ClusterProfiler | 4.0.5 |
| maftools | 2.8.0 |
| survival | 3.2.11 |
| surminer | 0.4.9 |
| timeROC | 0.4 |
| forestplot | 2.0.1 |
| Rms | 6.2.0 |
| immunedeconv | 2.0.3 |

Supplementary Figure 1. Mutations and copy number alterations of H2AFZ in HCC.

Supplementary Figure 2. Correlated expression of H2AFZ and DNA-damage-repair-related transcripts.

Supplementary Figure 3. Expression and survival outcome of H2AFZ-related regulators.

**Supplementary Figure 4. RT-qPCR and western blot assays.** P53 expression of negative control (NC)/TP53-siRNA1/TP53-siRNA2/TP53-siRNA3 Huh7 **(A)** and PLC/PRF/5 **(B)** cells. H2AFZ expression of negative control (NC)/H2AFZ-siRNA1/H2AFZ-siRNA2/H2AFZ-siRNA3 Huh7 **(C)** and PLC/PRF/5 **(D)** cells. P53 expression of TP53 (Y220C)-OE Huh7 cells **(E)** and TP53 (R249S)-OE PLC/PRF/5 cells **(F)**.

Supplementary Figure 5. The Th1/Th2^low^ group had significantly shorter OS time and PFS time compared to the Th1/Th2^high^ group (log-rank test, p<0.05). (A) Overall survival of the Th1/Th2^high^ group and the Th1/Th2^low^ group. (B) Progression-free survival of the Th1/Th2^high^ group and the Th1/Th2^low^ group.

Table 1. H2AFZ expression level in mutants and wild-types of the top 10 mutated genes in TCGA-LIHC.

Supplementary Table 1. Clinical data of TCGA-LIHC patients constructing Sankey gram in Figure 2B.

Supplementary Table 2. DEGs of H2AFZ^high^ and H2AFZ^low^ group.

Supplementary Table 3. Significant GO-BP annotations of DEGs.

Supplementary Table 4. Significant KEGG pathway annotations of DEGs.

Supplementary Table 5. Significant transcriptional factor (TF) annotations of DEGs.

## Supplementary Figures
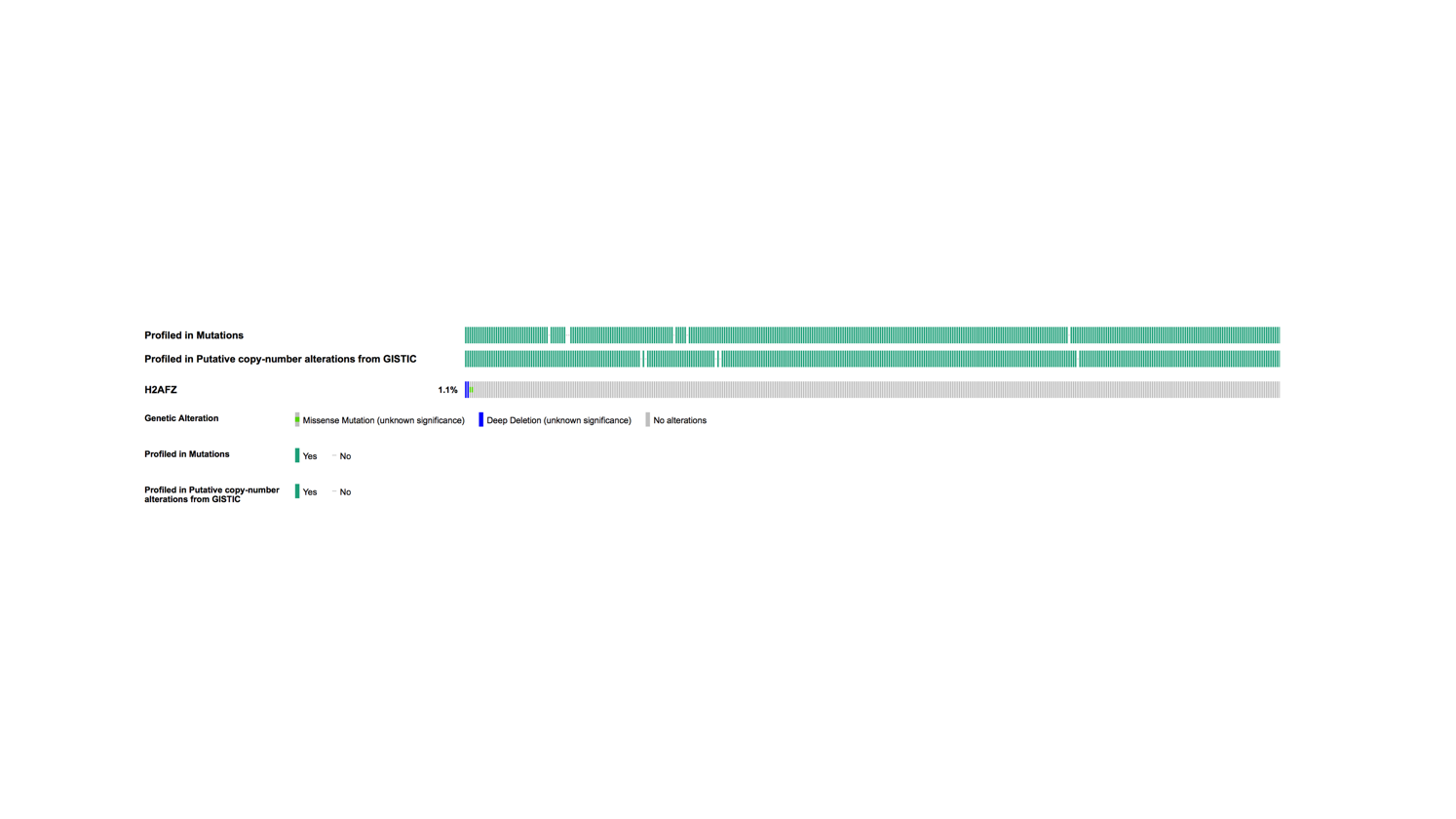
Supplementary Figure 1. Mutations and copy number alterations of H2AFZ in HCC.

##
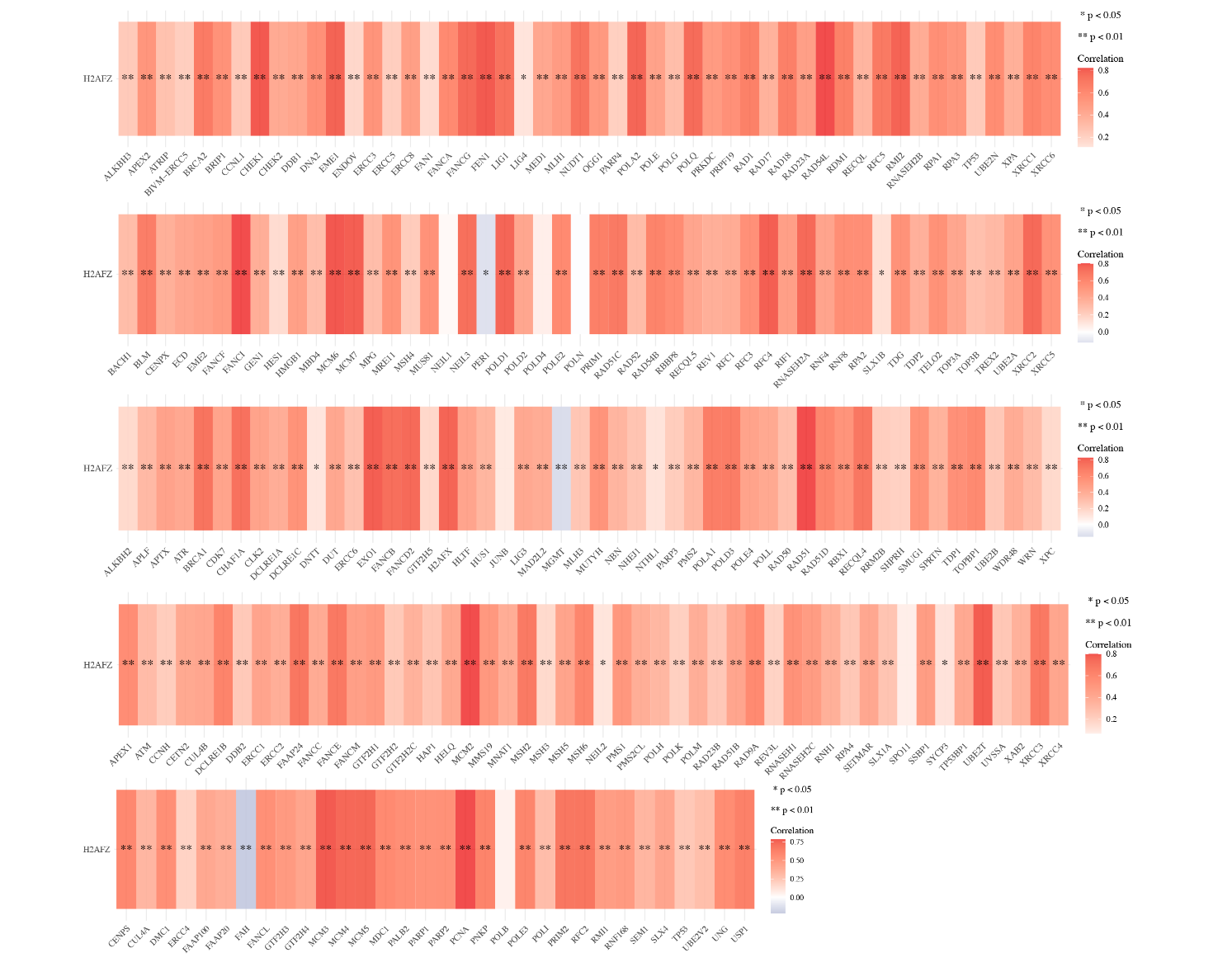
Supplementary Figure 2. Correlated expression of H2AFZ and DNA-damage-repair-related transcriptsSupplementary Figure 3. Expression and survival outcome of H2AFZ-related regulators


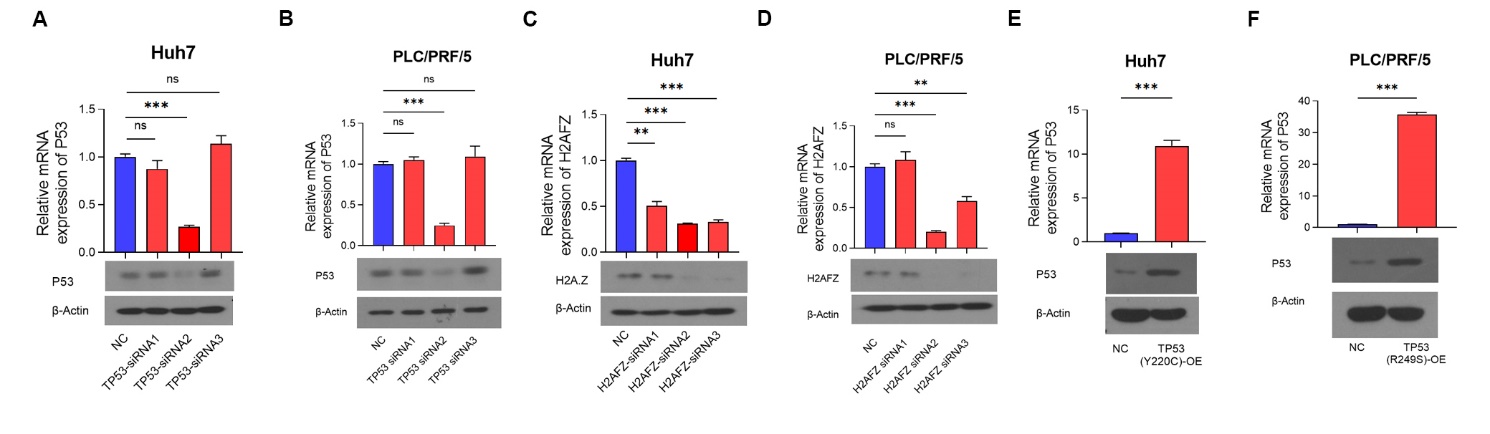


**Supplementary Figure 4. RT-qPCR and western blot assays.** P53 expression of negative control (NC)/TP53-siRNA1/TP53-siRNA2/TP53-siRNA3 Huh7 **(A)** and PLC/PRF/5 **(B)** cells. H2AFZ expression of negative control (NC)/H2AFZ-siRNA1/H2AFZ-siRNA2/H2AFZ-siRNA3 Huh7 **(C)** and PLC/PRF/5 **(D)** cells. P53 expression of TP53 (Y220C)-OE Huh7 cells **(E)** and TP53 (R249S)-OE PLC/PRF/5 cells **(F)**.

##

**Supplementary Figure 5.** The Th1/Th2^low^ group had significantly shorter OS time and PFS time compared to the Th1/Th2^high^ group (log-rank test, p<0.05). (A) Overall survival of the Th1/Th2^high^ group and the Th1/Th2^low^ group. (B) Progression-free survival of the Th1/Th2^high^ group and the Th1/Th2^low^ group.

**Supplementary Tables**

| **Barcode** | **Race** | **pT_stage** | **Grade** | **H2AFZ** | **Status** |
| --- | --- | --- | --- | --- | --- |
| **TCGA-DD-A3A3-01** | ASIAN | T1 | G2 | High exp | Dead |
| **TCGA-DD-A1EF-01** | WHITE | T1 | G3 | High exp | Dead |
| **TCGA-ED-A627-01** | WHITE | T1 | G2 | Low exp | Alive |
| **TCGA-DD-AACB-01** | ASIAN | T1 | G3 | High exp | Alive |
| **TCGA-G3-A3CG-01** | WHITE | T1 | G2 | Low exp | Alive |
| **TCGA-BC-A10W-01** | ASIAN | T4 | G3 | High exp | Dead |
| **TCGA-G3-A25Z-01** | ASIAN | T1 | G2 | Low exp | Alive |
| **TCGA-DD-AADY-01** | ASIAN | T1 | G2 | Low exp | Alive |
| **TCGA-DD-AAEH-01** | ASIAN | T1 | G2 | Low exp | Alive |
| **TCGA-G3-A5SI-01** | ASIAN | T2 | G2 | High exp | Dead |
| **TCGA-G3-A5SL-01** | WHITE | T2 | G2 | Low exp | Alive |
| **TCGA-EP-A12J-01** | BLACK | T1 | G1 | Low exp | Alive |
| **TCGA-DD-AAD2-01** | ASIAN | T1 | G2 | High exp | Alive |
| **TCGA-UB-A7MB-01** | WHITE | T2 | G3 | High exp | Alive |
| **TCGA-UB-A7MC-01** | WHITE | T3 | G3 | High exp | Alive |
| **TCGA-T1-A6J8-01** | WHITE | T1 | G2 | Low exp | Alive |
| **TCGA-BC-4072-01** | WHITE | T3 | G3 | High exp | Dead |
| **TCGA-DD-AACQ-01** | ASIAN | T2 | G3 | Low exp | Dead |
| **TCGA-2Y-A9GW-01** | WHITE | T1 | G2 | Low exp | Dead |
| **TCGA-DD-AAEI-01** | ASIAN | T1 | G2 | High exp | Alive |
| **TCGA-DD-AADN-01** | ASIAN | T1 | G4 | High exp | Alive |
| **TCGA-QA-A7B7-01** | BLACK | T2 | G2 | High exp | Alive |
| **TCGA-5C-A9VH-01** | WHITE | T1 | G2 | High exp | Alive |
| **TCGA-ZP-A9D4-01** | WHITE | T1 | G1 | Low exp | Alive |
| **TCGA-G3-AAV6-01** | WHITE | T3 | G3 | High exp | Dead |
| **TCGA-DD-A4NG-01** | WHITE | T3 | G2 | Low exp | Dead |
| **TCGA-ED-A8O5-01** | ASIAN | T3 | G3 | Low exp | Alive |
| **TCGA-DD-AACO-01** | ASIAN | T1 | G3 | High exp | Alive |
| **TCGA-DD-AACZ-01** | ASIAN | T1 | G4 | High exp | Dead |
| **TCGA-CC-5264-01** | ASIAN | T3 | G2 | High exp | Dead |
| **TCGA-DD-AAW3-01** | ASIAN | T1 | G2 | Low exp | Alive |
| **TCGA-DD-AAVX-01** | ASIAN | T2 | G2 | Low exp | Alive |
| **TCGA-2Y-A9HA-01** | WHITE | T2 | G2 | High exp | Dead |
| **TCGA-ED-A82E-01** | ASIAN | T3 | G2 | High exp | Alive |
| **TCGA-DD-AA3A-01** | WHITE | T1 | G4 | High exp | Dead |
| **TCGA-GJ-A3OU-01** | WHITE | T1 | G2 | High exp | Alive |
| **TCGA-G3-A7M9-01** | WHITE | T3 | G2 | High exp | Dead |
| **TCGA-EP-A3JL-01** | WHITE | T1 | G2 | Low exp | Alive |
| **TCGA-DD-AAEG-01** | ASIAN | T1 | G3 | Low exp | Alive |
| **TCGA-UB-A7MA-01** | WHITE | T2 | G2 | High exp | Alive |
| **TCGA-XR-A8TG-01** | WHITE | T1 | G2 | High exp | Alive |
| **TCGA-BC-A10X-01** | WHITE | T3 | G2 | Low exp | Dead |
| **TCGA-EP-A3RK-01** | WHITE | T3 | G2 | High exp | Alive |
| **TCGA-ED-A66Y-01** | ASIAN | T3 | G3 | High exp | Dead |
| **TCGA-DD-AADF-01** | ASIAN | T1 | G4 | High exp | Dead |
| **TCGA-CC-A9FV-01** | ASIAN | T3 | G2 | High exp | Alive |
| **TCGA-MI-A75G-01** | WHITE | T2 | G2 | Low exp | Alive |
| **TCGA-DD-A39V-01** | WHITE | T2 | G3 | High exp | Dead |
| **TCGA-DD-AACF-01** | ASIAN | T1 | G3 | High exp | Dead |
| **TCGA-G3-AAV0-01** | ASIAN | T1 | G2 | Low exp | Alive |
| **TCGA-KR-A7K2-01** | WHITE | T1 | G1 | Low exp | Alive |
| **TCGA-RC-A6M3-01** | AMERICAN INDIAN | T2 | G3 | High exp | Alive |
| **TCGA-DD-AACU-01** | ASIAN | T1 | G3 | Low exp | Alive |
| **TCGA-G3-A7M5-01** | ASIAN | T1 | G2 | Low exp | Alive |
| **TCGA-BW-A5NP-01** | WHITE | T2 | G3 | High exp | Alive |
| **TCGA-G3-A5SJ-01** | WHITE | T1 | G2 | High exp | Alive |
| **TCGA-DD-AACN-01** | ASIAN | T1 | G3 | Low exp | Alive |
| **TCGA-ED-A5KG-01** | ASIAN | T2 | G2 | High exp | Alive |
| **TCGA-G3-AAV5-01** | WHITE | T2 | G2 | High exp | Alive |
| **TCGA-NI-A4U2-01** | WHITE | T3 | G1 | Low exp | Dead |
| **TCGA-EP-A2KC-01** | BLACK | T1 | G3 | Low exp | Dead |
| **TCGA-RC-A6M5-01** | WHITE | T1 | G2 | Low exp | Alive |
| **TCGA-2Y-A9GU-01** | WHITE | T1 | G2 | High exp | Alive |
| **TCGA-G3-AAV2-01** | WHITE | T1 | G1 | Low exp | Alive |
| **TCGA-DD-AAEA-01** | ASIAN | T1 | G3 | Low exp | Alive |
| **TCGA-DD-A4ND-01** | WHITE | T1 | G3 | Low exp | Alive |
| **TCGA-DD-A4NO-01** | WHITE | T1 | G1 | Low exp | Alive |
| **TCGA-RC-A7SK-01** | ASIAN | T1 | G3 | Low exp | Alive |
| **TCGA-CC-A3MB-01** | ASIAN | T3 | G1 | High exp | Dead |
| **TCGA-DD-A4NA-01** | WHITE | T2 | G3 | High exp | Alive |
| **TCGA-DD-AAE2-01** | ASIAN | T1 | G3 | High exp | Alive |
| **TCGA-RG-A7D4-01** | BLACK | T2 | G2 | High exp | Alive |
| **TCGA-LG-A9QC-01** | WHITE | T1 | G2 | Low exp | Alive |
| **TCGA-DD-A3A9-01** | WHITE | T4 | G2 | Low exp | Dead |
| **TCGA-G3-A25V-01** | WHITE | T1 | G2 | Low exp | Alive |
| **TCGA-ZP-A9D1-01** | WHITE | T1 | G2 | Low exp | Alive |
| **TCGA-BC-A216-01** | WHITE | T3 | G2 | High exp | Alive |
| **TCGA-ZP-A9D2-01** | WHITE | T2 | G2 | High exp | Dead |
| **TCGA-ES-A2HS-01** | WHITE | T1 | G2 | Low exp | Dead |
| **TCGA-DD-AAVS-01** | ASIAN | T1 | G2 | Low exp | Alive |
| **TCGA-DD-A4NV-01** | WHITE | T3 | G1 | Low exp | Alive |
| **TCGA-BW-A5NQ-01** | WHITE | T1 | G3 | High exp | Alive |
| **TCGA-CC-A7IH-01** | ASIAN | T3 | G1 | High exp | Alive |
| **TCGA-WQ-A9G7-01** | WHITE | T3 | G3 | High exp | Alive |
| **TCGA-BC-A110-01** | BLACK | T1 | G1 | Low exp | Dead |
| **TCGA-RC-A7SB-01** | ASIAN | T2 | G2 | Low exp | Alive |
| **TCGA-BC-A10T-01** | WHITE | T4 | G1 | Low exp | Dead |
| **TCGA-MR-A8JO-01** | WHITE | T1 | G3 | Low exp | Alive |
| **TCGA-2Y-A9GX-01** | WHITE | T1 | G2 | High exp | Alive |
| **TCGA-K7-A5RG-01** | BLACK | T1 | G1 | Low exp | Alive |
| **TCGA-UB-AA0U-01** | WHITE | T2 | G2 | High exp | Alive |
| **TCGA-DD-AAD3-01** | ASIAN | T1 | G2 | Low exp | Alive |
| **TCGA-DD-A1ED-01** | WHITE | T1 | G1 | Low exp | Alive |
| **TCGA-DD-AAVZ-01** | ASIAN | T1 | G2 | Low exp | Alive |
| **TCGA-CC-A123-01** | ASIAN | T3 | G1 | Low exp | Alive |
| **TCGA-DD-A11B-01** | WHITE | T1 | G2 | Low exp | Dead |
| **TCGA-G3-A25Y-01** | ASIAN | T1 | G3 | High exp | Dead |
| **TCGA-DD-AADQ-01** | ASIAN | T2 | G3 | Low exp | Alive |
| **TCGA-2Y-A9GT-01** | WHITE | T1 | G2 | Low exp | Dead |
| **TCGA-DD-A119-01** | ASIAN | T3 | G3 | Low exp | Dead |
| **TCGA-DD-AADO-01** | ASIAN | T1 | G3 | Low exp | Alive |
| **TCGA-DD-A4NQ-01** | WHITE | T2 | G3 | High exp | Dead |
| **TCGA-DD-A4NL-01** | WHITE | T1 | G1 | Low exp | Alive |
| **TCGA-RC-A7SF-01** | ASIAN | T1 | G2 | Low exp | Alive |
| **TCGA-DD-A39Y-01** | ASIAN | T1 | G3 | High exp | Dead |
| **TCGA-ES-A2HT-01** | BLACK | T1 | G2 | Low exp | Dead |
| **TCGA-DD-AADA-01** | ASIAN | T1 | G3 | Low exp | Alive |
| **TCGA-DD-A4NI-01** | WHITE | T2 | G2 | Low exp | Alive |
| **TCGA-G3-A3CI-01** | WHITE | T1 | G2 | Low exp | Alive |
| **TCGA-WX-AA46-01** | WHITE | T2 | G1 | Low exp | Alive |
| **TCGA-DD-AACD-01** | ASIAN | T1 | G4 | Low exp | Dead |
| **TCGA-DD-A73G-01** | WHITE | T1 | G3 | High exp | Alive |
| **TCGA-2Y-A9H0-01** | WHITE | T3 | G1 | High exp | Alive |
| **TCGA-DD-AACC-01** | ASIAN | T1 | G2 | Low exp | Dead |
| **TCGA-DD-A1EE-01** | WHITE | T3 | G3 | High exp | Dead |
| **TCGA-FV-A4ZQ-01** | WHITE | T1 | G2 | High exp | Alive |
| **TCGA-G3-AAV3-01** | WHITE | T2 | G2 | High exp | Alive |
| **TCGA-BC-4073-01** | WHITE | T3 | G3 | High exp | Alive |
| **TCGA-G3-A3CH-01** | ASIAN | T3 | G2 | Low exp | Alive |
| **TCGA-G3-A3CK-01** | ASIAN | T1 | G2 | Low exp | Alive |
| **TCGA-DD-A116-01** | ASIAN | T3 | G3 | Low exp | Dead |
| **TCGA-5R-AA1C-01** | WHITE | T2 | G2 | Low exp | Alive |
| **TCGA-DD-AAE4-01** | ASIAN | T1 | G1 | High exp | Alive |
| **TCGA-DD-AACL-01** | ASIAN | T1 | G3 | High exp | Dead |
| **TCGA-DD-AADW-01** | ASIAN | T1 | G3 | High exp | Alive |
| **TCGA-CC-A3M9-01** | ASIAN | T3 | G3 | High exp | Dead |
| **TCGA-DD-A3A5-01** | WHITE | T3 | G2 | Low exp | Dead |
| **TCGA-FV-A3I1-01** | WHITE | T2 | G2 | High exp | Dead |
| **TCGA-DD-AAEB-01** | ASIAN | T1 | G2 | Low exp | Alive |
| **TCGA-DD-AAE1-01** | ASIAN | T1 | G3 | Low exp | Alive |
| **TCGA-DD-A39X-01** | WHITE | T1 | G2 | High exp | Dead |
| **TCGA-DD-AACG-01** | ASIAN | T2 | G4 | High exp | Dead |
| **TCGA-DD-A4NN-01** | WHITE | T1 | G3 | High exp | Dead |
| **TCGA-K7-A6G5-01** | WHITE | T1 | G2 | Low exp | Alive |
| **TCGA-2Y-A9H1-01** | WHITE | T1 | G2 | Low exp | Dead |
| **TCGA-MI-A75C-01** | WHITE | T1 | G3 | Low exp | Alive |
| **TCGA-ED-A66X-01** | ASIAN | T3 | G3 | High exp | Alive |
| **TCGA-DD-AAW1-01** | ASIAN | T3 | G2 | Low exp | Alive |
| **TCGA-DD-AADB-01** | ASIAN | T1 | G4 | High exp | Alive |
| **TCGA-G3-AAV1-01** | ASIAN | T4 | G3 | Low exp | Dead |
| **TCGA-DD-A4NJ-01** | WHITE | T2 | G2 | High exp | Alive |
| **TCGA-5C-AAPD-01** | ASIAN | T2 | G1 | High exp | Alive |
| **TCGA-EP-A26S-01** | WHITE | T1 | G2 | Low exp | Alive |
| **TCGA-BD-A2L6-01** | WHITE | T2 | G2 | High exp | Alive |
| **TCGA-DD-A1EK-01** | WHITE | T4 | G2 | High exp | Dead |
| **TCGA-RC-A6M4-01** | WHITE | T3 | G2 | High exp | Alive |
| **TCGA-DD-A1EH-01** | WHITE | T3 | G3 | Low exp | Alive |
| **TCGA-ED-A7PZ-01** | ASIAN | T2 | G2 | High exp | Alive |
| **TCGA-CC-A9FS-01** | ASIAN | T2 | G2 | Low exp | Alive |
| **TCGA-CC-A3MA-01** | ASIAN | T3 | G2 | High exp | Dead |
| **TCGA-DD-A4NH-01** | WHITE | T3 | G3 | High exp | Alive |
| **TCGA-FV-A2QR-01** | WHITE | T1 | G1 | High exp | Dead |
| **TCGA-2Y-A9H5-01** | WHITE | T1 | G3 | High exp | Dead |
| **TCGA-5C-A9VG-01** | WHITE | T2 | G2 | High exp | Alive |
| **TCGA-PD-A5DF-01** | WHITE | T4 | G2 | High exp | Dead |
| **TCGA-DD-AAD6-01** | ASIAN | T3 | G3 | Low exp | Alive |
| **TCGA-DD-A73F-01** | WHITE | T1 | G1 | High exp | Alive |
| **TCGA-DD-A1EI-01** | ASIAN | T1 | G2 | High exp | Alive |
| **TCGA-ZS-A9CF-01** | WHITE | T2 | G2 | High exp | Alive |
| **TCGA-RC-A7SH-01** | ASIAN | T2 | G3 | Low exp | Alive |
| **TCGA-ZS-A9CE-01** | WHITE | T2 | G1 | Low exp | Alive |
| **TCGA-DD-AAVV-01** | ASIAN | T2 | G3 | Low exp | Alive |
| **TCGA-CC-5259-01** | ASIAN | T4 | G2 | Low exp | Alive |
| **TCGA-G3-A7M7-01** | WHITE | T1 | G1 | Low exp | Alive |
| **TCGA-UB-A7ME-01** | ASIAN | T1 | G2 | High exp | Alive |
| **TCGA-DD-A4NK-01** | WHITE | T3 | G2 | Low exp | Dead |
| **TCGA-DD-AACS-01** | ASIAN | T1 | G3 | High exp | Alive |
| **TCGA-DD-A39W-01** | WHITE | T3 | G2 | Low exp | Dead |
| **TCGA-DD-AACV-01** | ASIAN | T1 | G3 | High exp | Alive |
| **TCGA-CC-A7IG-01** | ASIAN | T2 | G2 | High exp | Dead |
| **TCGA-DD-AADV-01** | ASIAN | T1 | G3 | High exp | Alive |
| **TCGA-CC-A8HS-01** | ASIAN | T3 | G1 | High exp | Dead |
| **TCGA-DD-AADJ-01** | ASIAN | T1 | G3 | Low exp | Alive |
| **TCGA-DD-AACA-01** | ASIAN | T1 | G3 | High exp | Alive |
| **TCGA-MI-A75E-01** | WHITE | T4 | G2 | Low exp | Alive |
| **TCGA-DD-A118-01** | WHITE | T2 | G2 | High exp | Alive |
| **TCGA-BC-A3KG-01** | WHITE | T2 | G3 | High exp | Alive |
| **TCGA-CC-5263-01** | ASIAN | T3 | G1 | High exp | Dead |
| **TCGA-BC-A69H-01** | WHITE | T2 | G3 | High exp | Alive |
| **TCGA-DD-A11A-01** | BLACK | T1 | G3 | Low exp | Alive |
| **TCGA-DD-AADR-01** | ASIAN | T1 | G3 | High exp | Alive |
| **TCGA-DD-A11C-01** | WHITE | T1 | G3 | Low exp | Alive |
| **TCGA-DD-AAD8-01** | ASIAN | T1 | G2 | High exp | Alive |
| **TCGA-CC-A8HV-01** | ASIAN | T2 | G2 | High exp | Dead |
| **TCGA-DD-AAVW-01** | ASIAN | T1 | G2 | Low exp | Alive |
| **TCGA-ZP-A9CV-01** | WHITE | T1 | G1 | Low exp | Dead |
| **TCGA-ED-A7XO-01** | ASIAN | T3 | G2 | High exp | Alive |
| **TCGA-2Y-A9GZ-01** | WHITE | T2 | G2 | Low exp | Dead |
| **TCGA-ZP-A9D0-01** | BLACK | T1 | G1 | Low exp | Alive |
| **TCGA-DD-AAEE-01** | ASIAN | T1 | G4 | High exp | Alive |
| **TCGA-ZS-A9CD-01** | WHITE | T2 | G2 | Low exp | Dead |
| **TCGA-CC-A8HU-01** | ASIAN | T3 | G3 | High exp | Dead |
| **TCGA-ZP-A9CY-01** | WHITE | T1 | G1 | Low exp | Alive |
| **TCGA-DD-A73B-01** | WHITE | T1 | G2 | High exp | Dead |
| **TCGA-MR-A520-01** | WHITE | T1 | G1 | Low exp | Alive |
| **TCGA-CC-A3MC-01** | ASIAN | T3 | G2 | High exp | Alive |
| **TCGA-DD-AAE0-01** | ASIAN | T3 | G4 | High exp | Alive |
| **TCGA-BC-A10S-01** | WHITE | T3 | G1 | Low exp | Dead |
| **TCGA-DD-A1EA-01** | ASIAN | T2 | G2 | Low exp | Alive |
| **TCGA-3K-AAZ8-01** | BLACK | T3 | G1 | Low exp | Alive |
| **TCGA-FV-A2QQ-01** | WHITE | T1 | G2 | Low exp | Alive |
| **TCGA-DD-AACI-01** | ASIAN | T2 | G3 | Low exp | Alive |
| **TCGA-DD-AAD0-01** | ASIAN | T1 | G2 | High exp | Alive |
| **TCGA-DD-A114-01** | BLACK | T2 | G3 | High exp | Dead |
| **TCGA-2Y-A9H6-01** | WHITE | T1 | G2 | Low exp | Alive |
| **TCGA-G3-A25U-01** | ASIAN | T1 | G3 | High exp | Alive |
| **TCGA-ZS-A9CG-01** | WHITE | T2 | G2 | Low exp | Alive |
| **TCGA-DD-A1EG-01** | WHITE | T1 | G3 | High exp | Dead |
| **TCGA-RC-A7S9-01** | ASIAN | T1 | G3 | High exp | Alive |
| **TCGA-CC-A8HT-01** | ASIAN | T3 | G2 | High exp | Dead |
| **TCGA-2Y-A9H2-01** | WHITE | T1 | G3 | High exp | Alive |
| **TCGA-DD-AADC-01** | ASIAN | T1 | G3 | High exp | Dead |
| **TCGA-EP-A2KA-01** | WHITE | T3 | G3 | High exp | Dead |
| **TCGA-WQ-AB4B-01** | WHITE | T2 | G2 | Low exp | Alive |
| **TCGA-DD-AACW-01** | ASIAN | T1 | G3 | Low exp | Alive |
| **TCGA-DD-AAE7-01** | ASIAN | T1 | G2 | Low exp | Alive |
| **TCGA-DD-AACH-01** | ASIAN | T2 | G3 | High exp | Dead |
| **TCGA-LG-A9QD-01** | WHITE | T3 | G2 | Low exp | Alive |
| **TCGA-BC-A3KF-01** | WHITE | T1 | G2 | Low exp | Alive |
| **TCGA-DD-A115-01** | WHITE | T3 | G2 | High exp | Dead |
| **TCGA-2Y-A9H3-01** | WHITE | T2 | G1 | Low exp | Alive |
| **TCGA-BC-A217-01** | WHITE | T2 | G3 | High exp | Dead |
| **TCGA-G3-AAV4-01** | WHITE | T1 | G1 | Low exp | Dead |
| **TCGA-DD-AADI-01** | ASIAN | T1 | G3 | Low exp | Alive |
| **TCGA-FV-A3R3-01** | WHITE | T1 | G2 | Low exp | Dead |
| **TCGA-CC-5261-01** | ASIAN | T2 | G2 | High exp | Dead |
| **TCGA-BW-A5NO-01** | BLACK | T3 | G2 | Low exp | Alive |
| **TCGA-DD-A4NB-01** | WHITE | T1 | G2 | Low exp | Alive |
| **TCGA-2Y-A9H4-01** | BLACK | T1 | G2 | High exp | Alive |
| **TCGA-DD-AAVY-01** | ASIAN | T3 | G2 | Low exp | Alive |
| **TCGA-XR-A8TD-01** | WHITE | T3 | G3 | High exp | Alive |
| **TCGA-DD-AADG-01** | ASIAN | T3 | G3 | Low exp | Alive |
| **TCGA-DD-A4NE-01** | WHITE | T3 | G3 | High exp | Dead |
| **TCGA-2Y-A9H7-01** | WHITE | T1 | G2 | High exp | Alive |
| **TCGA-ED-A7PY-01** | ASIAN | T2 | G3 | Low exp | Alive |
| **TCGA-G3-A25S-01** | WHITE | T1 | G2 | High exp | Dead |
| **TCGA-DD-AAE3-01** | ASIAN | T1 | G2 | Low exp | Alive |
| **TCGA-YA-A8S7-01** | WHITE | T3 | G3 | High exp | Dead |
| **TCGA-CC-5260-01** | ASIAN | T4 | G1 | High exp | Dead |
| **TCGA-K7-A5RF-01** | WHITE | T1 | G1 | Low exp | Alive |
| **TCGA-CC-A5UC-01** | ASIAN | T3 | G3 | High exp | Dead |
| **TCGA-DD-A3A6-01** | WHITE | T2 | G2 | Low exp | Dead |
| **TCGA-FV-A3I0-01** | WHITE | T2 | G2 | High exp | Alive |
| **TCGA-CC-A7IF-01** | ASIAN | T3 | G1 | High exp | Dead |
| **TCGA-FV-A496-01** | WHITE | T1 | G2 | Low exp | Alive |
| **TCGA-DD-A4NP-01** | WHITE | T1 | G3 | Low exp | Alive |
| **TCGA-DD-AAED-01** | ASIAN | T1 | G3 | Low exp | Alive |
| **TCGA-DD-A73E-01** | WHITE | T1 | G1 | High exp | Alive |
| **TCGA-CC-A5UD-01** | ASIAN | T3 | G2 | High exp | Dead |
| **TCGA-DD-AACK-01** | ASIAN | T1 | G2 | High exp | Alive |
| **TCGA-NI-A8LF-01** | WHITE | T1 | G3 | Low exp | Alive |
| **TCGA-ED-A4XI-01** | ASIAN | T2 | G3 | Low exp | Alive |
| **TCGA-BC-A5W4-01** | WHITE | T3 | G3 | Low exp | Dead |
| **TCGA-DD-A4NF-01** | WHITE | T1 | G2 | Low exp | Alive |
| **TCGA-DD-AACT-01** | ASIAN | T1 | G2 | Low exp | Alive |
| **TCGA-DD-AADK-01** | ASIAN | T2 | G3 | High exp | Alive |
| **TCGA-BC-A8YO-01** | WHITE | T4 | G3 | High exp | Alive |
| **TCGA-G3-A5SK-01** | WHITE | T1 | G1 | Low exp | Alive |
| **TCGA-DD-A3A2-01** | WHITE | T1 | G1 | Low exp | Dead |
| **TCGA-CC-5262-01** | ASIAN | T4 | G1 | High exp | Dead |
| **TCGA-BC-A10Y-01** | WHITE | T4 | G3 | High exp | Dead |
| **TCGA-FV-A495-01** | WHITE | T2 | G2 | High exp | Alive |
| **TCGA-KR-A7K7-01** | WHITE | T2 | G1 | High exp | Alive |
| **TCGA-CC-5258-01** | ASIAN | T2 | G2 | High exp | Dead |
| **TCGA-5R-AA1D-01** | WHITE | T3 | G3 | Low exp | Alive |
| **TCGA-ED-A97K-01** | ASIAN | T3 | G2 | Low exp | Alive |
| **TCGA-LG-A6GG-01** | WHITE | T2 | G2 | Low exp | Alive |
| **TCGA-EP-A2KB-01** | WHITE | T1 | G2 | Low exp | Dead |
| **TCGA-WJ-A86L-01** | WHITE | T1 | G2 | Low exp | Alive |
| **TCGA-2Y-A9GY-01** | WHITE | T2 | G3 | High exp | Dead |
| **TCGA-DD-AADM-01** | ASIAN | T2 | G3 | High exp | Dead |
| **TCGA-DD-AACJ-01** | ASIAN | T2 | G2 | Low exp | Alive |
| **TCGA-CC-A7IE-01** | ASIAN | T3 | G2 | Low exp | Dead |
| **TCGA-GJ-A6C0-01** | WHITE | T2 | G2 | High exp | Dead |
| **TCGA-DD-A1EJ-01** | WHITE | T1 | G2 | High exp | Dead |
| **TCGA-XR-A8TF-01** | WHITE | T1 | G1 | Low exp | Dead |
| **TCGA-2V-A95S-01** | ASIAN | T2 | G3 | High exp | Alive |
| **TCGA-K7-AAU7-01** | WHITE | T2 | G2 | High exp | Alive |
| **TCGA-CC-A7IL-01** | ASIAN | T3 | G1 | Low exp | Dead |
| **TCGA-G3-AAV7-01** | ASIAN | T2 | G2 | High exp | Alive |
| **TCGA-DD-A11D-01** | WHITE | T1 | G2 | Low exp | Dead |
| **TCGA-CC-A9FU-01** | ASIAN | T3 | G2 | High exp | Alive |
| **TCGA-DD-AAE6-01** | ASIAN | T1 | G2 | Low exp | Alive |
| **TCGA-DD-AADL-01** | ASIAN | T1 | G4 | Low exp | Alive |
| **TCGA-DD-AADS-01** | ASIAN | T1 | G2 | Low exp | Alive |
| **TCGA-CC-A7II-01** | ASIAN | T3 | G3 | High exp | Alive |
| **TCGA-5R-AAAM-01** | WHITE | T2 | G2 | Low exp | Dead |
| **TCGA-CC-A7IK-01** | ASIAN | T3 | G3 | High exp | Dead |
| **TCGA-GJ-A9DB-01** | WHITE | T1 | G2 | High exp | Dead |
| **TCGA-G3-A25T-01** | WHITE | T3 | G2 | High exp | Alive |
| **TCGA-G3-A7M6-01** | WHITE | T1 | G3 | High exp | Alive |
| **TCGA-DD-A73C-01** | WHITE | T3 | G1 | Low exp | Alive |
| **TCGA-DD-AAVR-01** | ASIAN | T1 | G2 | Low exp | Alive |
| **TCGA-DD-AAVP-01** | ASIAN | T1 | G1 | High exp | Alive |
| **TCGA-DD-A73A-01** | WHITE | T1 | G2 | Low exp | Alive |
| **TCGA-2Y-A9GV-01** | WHITE | T1 | G1 | Low exp | Dead |
| **TCGA-DD-AAE9-01** | ASIAN | T1 | G3 | High exp | Alive |
| **TCGA-DD-AAD1-01** | ASIAN | T1 | G4 | High exp | Alive |
| **TCGA-UB-A7MF-01** | WHITE | T3 | G2 | High exp | Dead |
| **TCGA-4R-AA8I-01** | WHITE | T2 | G2 | High exp | Dead |
| **TCGA-BC-A112-01** | WHITE | T3 | G2 | High exp | Dead |
| **TCGA-ED-A459-01** | ASIAN | T2 | G2 | High exp | Alive |
| **TCGA-BD-A3EP-01** | BLACK | T1 | G2 | Low exp | Alive |
| **TCGA-G3-A6UC-01** | WHITE | T3 | G2 | Low exp | Alive |
| **TCGA-2Y-A9H9-01** | WHITE | T1 | G2 | High exp | Alive |
| **TCGA-DD-AAEK-01** | ASIAN | T2 | G3 | Low exp | Alive |
| **TCGA-DD-AAD5-01** | ASIAN | T1 | G3 | High exp | Alive |
| **TCGA-DD-AAW0-01** | ASIAN | T1 | G2 | Low exp | Alive |
| **TCGA-DD-AAC9-01** | ASIAN | T1 | G2 | Low exp | Alive |
| **TCGA-DD-AAC8-01** | ASIAN | T1 | G3 | Low exp | Dead |
| **TCGA-XR-A8TE-01** | WHITE | T3 | G1 | Low exp | Alive |
| **TCGA-DD-AAVU-01** | ASIAN | T2 | G2 | Low exp | Alive |
| **TCGA-2Y-A9GS-01** | WHITE | T2 | G2 | High exp | Dead |
| **TCGA-DD-A73D-01** | WHITE | T2 | G1 | Low exp | Alive |
| **TCGA-KR-A7K0-01** | WHITE | T1 | G1 | Low exp | Dead |
| **TCGA-WX-AA44-01** | WHITE | T1 | G3 | High exp | Alive |
| **TCGA-ED-A8O6-01** | ASIAN | T3 | G3 | High exp | Dead |
| **TCGA-DD-AADU-01** | ASIAN | T2 | G3 | Low exp | Alive |
| **TCGA-2Y-A9H8-01** | WHITE | T1 | G2 | High exp | Dead |
| **TCGA-G3-A3CJ-01** | AMERICAN INDIAN | T2 | G2 | Low exp | Alive |
| **TCGA-DD-A4NS-01** | WHITE | T1 | G2 | Low exp | Dead |
| **TCGA-DD-AACP-01** | ASIAN | T1 | G3 | High exp | Alive |
| **TCGA-G3-A5SM-01** | WHITE | T2 | G3 | Low exp | Alive |
| **TCGA-DD-AAVQ-01** | ASIAN | T1 | G2 | High exp | Alive |
| **TCGA-DD-AACE-01** | ASIAN | T1 | G3 | Low exp | Alive |
| **TCGA-G3-A7M8-01** | ASIAN | T1 | G1 | Low exp | Alive |
| **TCGA-BD-A3ER-01** | WHITE | T2 | G2 | Low exp | Alive |
| **TCGA-DD-A113-01** | WHITE | T2 | G3 | High exp | Alive |
| **TCGA-DD-A3A8-01** | WHITE | T2 | G2 | Low exp | Dead |
| **TCGA-ED-A7PX-01** | ASIAN | T2 | G3 | High exp | Alive |
| **TCGA-DD-AADP-01** | ASIAN | T1 | G3 | Low exp | Alive |
| **TCGA-CC-A7IJ-01** | ASIAN | T2 | G3 | High exp | Alive |
| **TCGA-BC-A10U-01** | WHITE | T2 | G2 | Low exp | Dead |
| **TCGA-DD-AADD-01** | ASIAN | T1 | G4 | High exp | Alive |
| **TCGA-ZP-A9CZ-01** | ASIAN | T1 | G1 | High exp | Alive |
| **TCGA-DD-AACY-01** | ASIAN | T1 | G3 | Low exp | Alive |
| **TCGA-DD-A1EC-01** | WHITE | T1 | G3 | High exp | Alive |
| **TCGA-DD-A1EL-01** | BLACK | T2 | G3 | High exp | Dead |
| **TCGA-RC-A6M6-01** | WHITE | T2 | G3 | High exp | Alive |
| **TCGA-MI-A75I-01** | BLACK | T2 | G1 | High exp | Alive |
| **TCGA-UB-A7MD-01** | BLACK | T1 | G3 | High exp | Dead |
| **TCGA-CC-A1HT-01** | ASIAN | T3 | G3 | High exp | Dead |
| **TCGA-WX-AA47-01** | WHITE | T3 | G2 | Low exp | Dead |
| **TCGA-FV-A4ZP-01** | WHITE | T3 | G2 | High exp | Dead |
| **TCGA-ED-A7XP-01** | ASIAN | T2 | G3 | Low exp | Alive |
| **TCGA-CC-A5UE-01** | ASIAN | T4 | G2 | High exp | Dead |
| **TCGA-CC-A9FW-01** | ASIAN | T3 | G2 | High exp | Alive |
| **TCGA-XR-A8TC-01** | WHITE | T1 | G2 | Low exp | Alive |
| **TCGA-BC-A69I-01** | WHITE | T1 | G1 | Low exp | Alive |
| **TCGA-DD-AACX-01** | ASIAN | T2 | G3 | High exp | Alive |
| **TCGA-DD-AAW2-01** | ASIAN | T1 | G2 | Low exp | Alive |
| **TCGA-BC-A10R-01** | WHITE | T3 | G2 | Low exp | Dead |
| **TCGA-G3-A25X-01** | ASIAN | T2 | G3 | High exp | Alive |
| **TCGA-DD-A3A4-01** | WHITE | T3 | G3 | Low exp | Dead |
| **TCGA-BC-A10Z-01** | WHITE | T1 | G2 | Low exp | Dead |
| **TCGA-DD-A4NR-01** | WHITE | T1 | G3 | High exp | Dead |

**Supplementary Table 1.** Clinical data of TCGA-LIHC patients constructing Sankey gram in Figure 2B.

| **Genes** |  | **logFC** | **AveExpr** | **t** | **P.Value** | **adj.P.Val** |
| --- | --- | --- | --- | --- | --- | --- |
| H2AFZ | Up | 1.57E+00 | 6.63E+00 | 2.82E+01 | 3.26E-94 | 5.40E-90 |
| KIF2C | Up | 1.98E+00 | 2.59E+00 | 2.04E+01 | 8.57E-63 | 7.10E-59 |
| ZWINT | Up | 1.86E+00 | 3.94E+00 | 2.01E+01 | 2.05E-61 | 9.53E-58 |
| CDT1 | Up | 1.92E+00 | 3.00E+00 | 2.01E+01 | 2.30E-61 | 9.53E-58 |
| CENPM | Up | 1.98E+00 | 2.99E+00 | 2.01E+01 | 3.73E-61 | 1.24E-57 |
| MAD2L1 | Up | 1.43E+00 | 2.04E+00 | 2.00E+01 | 1.02E-60 | 2.81E-57 |
| TACC3 | Up | 1.65E+00 | 3.31E+00 | 1.99E+01 | 1.88E-60 | 4.11E-57 |
| CDCA5 | Up | 1.77E+00 | 2.89E+00 | 1.99E+01 | 1.98E-60 | 4.11E-57 |
| CDC20 | Up | 2.39E+00 | 3.95E+00 | 1.98E+01 | 2.94E-60 | 5.40E-57 |
| PLK1 | Up | 1.90E+00 | 2.48E+00 | 1.98E+01 | 4.60E-60 | 7.63E-57 |
| BIRC5 | Up | 2.17E+00 | 3.57E+00 | 1.98E+01 | 6.16E-60 | 9.29E-57 |
| CDC45 | Up | 1.66E+00 | 2.42E+00 | 1.97E+01 | 1.08E-59 | 1.45E-56 |
| ORC1 | Up | 1.52E+00 | 1.86E+00 | 1.97E+01 | 1.14E-59 | 1.45E-56 |
| CCNB1 | Up | 2.00E+00 | 4.10E+00 | 1.97E+01 | 1.37E-59 | 1.54E-56 |
| PKMYT1 | Up | 1.44E+00 | 1.85E+00 | 1.97E+01 | 1.39E-59 | 1.54E-56 |
| KIF4A | Up | 1.86E+00 | 2.51E+00 | 1.93E+01 | 3.67E-58 | 3.80E-55 |
| CENPA | Up | 1.71E+00 | 2.03E+00 | 1.92E+01 | 1.30E-57 | 1.27E-54 |
| MELK | Up | 1.77E+00 | 2.46E+00 | 1.92E+01 | 1.53E-57 | 1.41E-54 |
| RAD51 | Up | 1.35E+00 | 1.76E+00 | 1.92E+01 | 1.79E-57 | 1.56E-54 |
| CCNB2 | Up | 1.92E+00 | 2.99E+00 | 1.92E+01 | 1.88E-57 | 1.56E-54 |
| PCLAF | Up | 1.62E+00 | 2.58E+00 | 1.90E+01 | 1.03E-56 | 8.16E-54 |
| EXO1 | Up | 1.44E+00 | 1.79E+00 | 1.89E+01 | 2.55E-56 | 1.92E-53 |
| CCNF | Up | 1.30E+00 | 2.03E+00 | 1.89E+01 | 3.00E-56 | 2.16E-53 |
| KIFC1 | Up | 2.00E+00 | 3.25E+00 | 1.89E+01 | 4.06E-56 | 2.80E-53 |
| TROAP | Up | 1.82E+00 | 2.41E+00 | 1.88E+01 | 6.79E-56 | 4.44E-53 |
| CDK1 | Up | 1.88E+00 | 3.06E+00 | 1.88E+01 | 6.96E-56 | 4.44E-53 |
| SPC25 | Up | 1.51E+00 | 2.23E+00 | 1.87E+01 | 1.17E-55 | 7.15E-53 |
| BUB1 | Up | 1.54E+00 | 1.94E+00 | 1.87E+01 | 1.92E-55 | 1.14E-52 |
| TPX2 | Up | 2.00E+00 | 4.04E+00 | 1.87E+01 | 2.10E-55 | 1.20E-52 |
| GINS1 | Up | 1.71E+00 | 2.43E+00 | 1.87E+01 | 2.25E-55 | 1.24E-52 |
| TYMS | Up | 1.83E+00 | 4.07E+00 | 1.87E+01 | 2.37E-55 | 1.27E-52 |
| CDCA8 | Up | 1.80E+00 | 2.96E+00 | 1.87E+01 | 2.69E-55 | 1.39E-52 |
| NCAPG | Up | 1.69E+00 | 2.30E+00 | 1.85E+01 | 8.35E-55 | 4.19E-52 |
| MCM2 | Up | 1.89E+00 | 3.83E+00 | 1.84E+01 | 2.35E-54 | 1.14E-51 |
| SGO1 | Up | 1.27E+00 | 1.44E+00 | 1.84E+01 | 2.68E-54 | 1.27E-51 |
| TEDC2 | Up | 1.47E+00 | 2.18E+00 | 1.84E+01 | 3.74E-54 | 1.72E-51 |
| NDC80 | Up | 1.59E+00 | 2.45E+00 | 1.83E+01 | 7.70E-54 | 3.45E-51 |
| MCM10 | Up | 1.32E+00 | 1.33E+00 | 1.83E+01 | 9.79E-54 | 4.27E-51 |
| WDR62 | Up | 1.17E+00 | 1.38E+00 | 1.82E+01 | 1.68E-53 | 7.12E-51 |
| NCAPH | Up | 1.62E+00 | 2.22E+00 | 1.81E+01 | 4.64E-53 | 1.92E-50 |
| CHAF1B | Up | 1.50E+00 | 1.84E+00 | 1.81E+01 | 5.02E-53 | 2.03E-50 |
| CDC6 | Up | 1.73E+00 | 2.60E+00 | 1.81E+01 | 5.23E-53 | 2.06E-50 |
| UHRF1 | Up | 1.59E+00 | 1.75E+00 | 1.80E+01 | 9.90E-53 | 3.81E-50 |
| NEK2 | Up | 1.78E+00 | 2.61E+00 | 1.80E+01 | 2.54E-52 | 9.56E-50 |
| RRM2 | Up | 1.83E+00 | 3.62E+00 | 1.79E+01 | 4.25E-52 | 1.56E-49 |
| DLGAP5 | Up | 1.64E+00 | 2.11E+00 | 1.79E+01 | 4.68E-52 | 1.68E-49 |
| SKA3 | Up | 1.48E+00 | 1.96E+00 | 1.79E+01 | 5.28E-52 | 1.86E-49 |
| FANCI | Up | 1.39E+00 | 2.21E+00 | 1.79E+01 | 5.46E-52 | 1.89E-49 |
| HJURP | Up | 1.69E+00 | 2.37E+00 | 1.78E+01 | 6.87E-52 | 2.32E-49 |
| SKA1 | Up | 1.67E+00 | 2.13E+00 | 1.78E+01 | 1.08E-51 | 3.57E-49 |
| EZH2 | Up | 1.40E+00 | 2.80E+00 | 1.77E+01 | 1.78E-51 | 5.79E-49 |
| KIF18B | Up | 1.64E+00 | 1.88E+00 | 1.77E+01 | 2.45E-51 | 7.81E-49 |
| FEN1 | Up | 1.37E+00 | 4.79E+00 | 1.77E+01 | 3.81E-51 | 1.19E-48 |
| CHEK1 | Up | 1.18E+00 | 1.92E+00 | 1.77E+01 | 3.95E-51 | 1.21E-48 |
| STMN1 | Up | 1.61E+00 | 4.73E+00 | 1.77E+01 | 4.16E-51 | 1.25E-48 |
| GINS2 | Up | 1.40E+00 | 2.68E+00 | 1.77E+01 | 4.23E-51 | 1.25E-48 |
| MKI67 | Up | 1.74E+00 | 2.53E+00 | 1.76E+01 | 5.41E-51 | 1.57E-48 |
| PRR11 | Up | 1.53E+00 | 2.01E+00 | 1.76E+01 | 7.45E-51 | 2.13E-48 |
| MYBL2 | Up | 2.48E+00 | 3.60E+00 | 1.76E+01 | 1.04E-50 | 2.91E-48 |
| ASF1B | Up | 1.80E+00 | 3.11E+00 | 1.76E+01 | 1.13E-50 | 3.13E-48 |
| UBE2T | Up | 1.65E+00 | 4.19E+00 | 1.75E+01 | 1.46E-50 | 3.96E-48 |
| KIF11 | Up | 1.46E+00 | 2.22E+00 | 1.75E+01 | 1.55E-50 | 4.15E-48 |
| CDCA3 | Up | 1.39E+00 | 1.92E+00 | 1.75E+01 | 1.81E-50 | 4.77E-48 |
| BUB1B | Up | 1.59E+00 | 1.94E+00 | 1.75E+01 | 2.21E-50 | 5.73E-48 |
| RAD54L | Up | 1.38E+00 | 1.37E+00 | 1.74E+01 | 3.60E-50 | 9.18E-48 |
| PTTG1 | Up | 1.97E+00 | 3.98E+00 | 1.74E+01 | 6.27E-50 | 1.57E-47 |
| E2F2 | Up | 1.05E+00 | 1.10E+00 | 1.74E+01 | 6.76E-50 | 1.67E-47 |
| AURKB | Up | 1.83E+00 | 2.93E+00 | 1.74E+01 | 7.09E-50 | 1.73E-47 |
| DTL | Up | 1.62E+00 | 2.47E+00 | 1.73E+01 | 1.02E-49 | 2.45E-47 |
| RFC4 | Up | 1.34E+00 | 3.67E+00 | 1.73E+01 | 1.15E-49 | 2.73E-47 |
| MCM6 | Up | 1.50E+00 | 4.13E+00 | 1.73E+01 | 1.59E-49 | 3.70E-47 |
| SHCBP1 | Up | 1.39E+00 | 1.49E+00 | 1.73E+01 | 1.66E-49 | 3.83E-47 |
| CENPH | Up | 1.21E+00 | 2.45E+00 | 1.73E+01 | 1.69E-49 | 3.83E-47 |
| PHF19 | Up | 1.25E+00 | 2.35E+00 | 1.73E+01 | 1.79E-49 | 4.00E-47 |
| CENPE | Up | 1.12E+00 | 1.20E+00 | 1.72E+01 | 3.00E-49 | 6.62E-47 |
| KIF23 | Up | 1.45E+00 | 1.65E+00 | 1.72E+01 | 4.27E-49 | 9.31E-47 |
| CDC25C | Up | 1.47E+00 | 2.03E+00 | 1.72E+01 | 5.58E-49 | 1.19E-46 |
| RMI2 | Up | 1.58E+00 | 2.57E+00 | 1.72E+01 | 5.58E-49 | 1.19E-46 |
| TOP2A | Up | 2.13E+00 | 3.66E+00 | 1.70E+01 | 1.51E-48 | 3.16E-46 |
| TRIP13 | Up | 1.55E+00 | 2.01E+00 | 1.70E+01 | 1.83E-48 | 3.79E-46 |
| H2AFX | Up | 1.45E+00 | 5.20E+00 | 1.70E+01 | 2.50E-48 | 5.12E-46 |
| RACGAP1 | Up | 1.46E+00 | 3.06E+00 | 1.70E+01 | 3.31E-48 | 6.68E-46 |
| NUF2 | Up | 1.66E+00 | 2.24E+00 | 1.69E+01 | 4.39E-48 | 8.76E-46 |
| CENPU | Up | 1.51E+00 | 2.96E+00 | 1.69E+01 | 7.21E-48 | 1.42E-45 |
| CCNA2 | Up | 1.84E+00 | 3.12E+00 | 1.69E+01 | 7.39E-48 | 1.44E-45 |
| CKAP2L | Up | 1.33E+00 | 1.60E+00 | 1.69E+01 | 7.79E-48 | 1.50E-45 |
| CDC25A | Up | 1.30E+00 | 1.63E+00 | 1.68E+01 | 1.14E-47 | 2.18E-45 |
| ORC6 | Up | 1.19E+00 | 1.39E+00 | 1.68E+01 | 1.71E-47 | 3.22E-45 |
| DEPDC1B | Up | 1.67E+00 | 1.86E+00 | 1.68E+01 | 1.91E-47 | 3.56E-45 |
| FOXM1 | Up | 1.86E+00 | 2.90E+00 | 1.68E+01 | 2.00E-47 | 3.69E-45 |
| MCM7 | Up | 1.36E+00 | 5.44E+00 | 1.67E+01 | 2.98E-47 | 5.36E-45 |
| PRC1 | Up | 1.65E+00 | 2.96E+00 | 1.67E+01 | 4.97E-47 | 8.86E-45 |
| ARHGAP11A | Up | 1.34E+00 | 1.92E+00 | 1.66E+01 | 6.85E-47 | 1.21E-44 |
| TCF19 | Up | 1.70E+00 | 3.47E+00 | 1.66E+01 | 8.63E-47 | 1.51E-44 |
| PLK4 | Up | 1.05E+00 | 1.28E+00 | 1.66E+01 | 9.05E-47 | 1.56E-44 |
| POLA2 | Up | 1.03E+00 | 2.77E+00 | 1.66E+01 | 9.48E-47 | 1.62E-44 |
| FANCG | Up | 1.16E+00 | 2.93E+00 | 1.66E+01 | 1.10E-46 | 1.86E-44 |
| POLD1 | Up | 1.17E+00 | 3.51E+00 | 1.66E+01 | 1.42E-46 | 2.38E-44 |
| NUSAP1 | Up | 1.72E+00 | 4.07E+00 | 1.65E+01 | 1.79E-46 | 2.96E-44 |
| FANCD2 | Up | 1.17E+00 | 1.61E+00 | 1.65E+01 | 2.14E-46 | 3.51E-44 |
| DEPDC1 | Up | 1.36E+00 | 1.48E+00 | 1.65E+01 | 2.19E-46 | 3.56E-44 |
| TTK | Up | 1.45E+00 | 1.72E+00 | 1.65E+01 | 2.22E-46 | 3.56E-44 |
| EME1 | Up | 1.14E+00 | 1.39E+00 | 1.65E+01 | 2.45E-46 | 3.90E-44 |
| TK1 | Up | 1.69E+00 | 5.23E+00 | 1.65E+01 | 2.97E-46 | 4.69E-44 |
| PARPBP | Up | 1.08E+00 | 1.40E+00 | 1.64E+01 | 8.70E-46 | 1.36E-43 |
| LMNB1 | Up | 1.68E+00 | 3.83E+00 | 1.64E+01 | 9.07E-46 | 1.40E-43 |
| MCM3 | Up | 1.43E+00 | 5.46E+00 | 1.64E+01 | 1.05E-45 | 1.61E-43 |
| KIF20A | Up | 1.70E+00 | 2.59E+00 | 1.63E+01 | 1.78E-45 | 2.71E-43 |
| OIP5 | Up | 1.41E+00 | 2.22E+00 | 1.63E+01 | 1.80E-45 | 2.71E-43 |
| CIP2A | Up | 1.21E+00 | 1.39E+00 | 1.62E+01 | 4.72E-45 | 7.04E-43 |
| PCNA | Up | 1.21E+00 | 6.72E+00 | 1.62E+01 | 6.00E-45 | 8.88E-43 |
| CENPF | Up | 1.62E+00 | 2.34E+00 | 1.61E+01 | 1.14E-44 | 1.67E-42 |
| GTSE1 | Up | 1.47E+00 | 1.80E+00 | 1.61E+01 | 1.48E-44 | 2.14E-42 |
| ANLN | Up | 1.66E+00 | 2.26E+00 | 1.61E+01 | 1.50E-44 | 2.17E-42 |
| CEP55 | Up | 1.48E+00 | 1.77E+00 | 1.61E+01 | 1.61E-44 | 2.29E-42 |
| MCM4 | Up | 1.47E+00 | 4.00E+00 | 1.61E+01 | 1.90E-44 | 2.69E-42 |
| UBE2C | Up | 2.13E+00 | 4.31E+00 | 1.60E+01 | 2.37E-44 | 3.33E-42 |
| CDKN3 | Up | 1.73E+00 | 3.34E+00 | 1.60E+01 | 2.79E-44 | 3.89E-42 |
| LIG1 | Up | 1.10E+00 | 3.57E+00 | 1.60E+01 | 4.43E-44 | 6.11E-42 |
| KIF18A | Up | 1.14E+00 | 1.19E+00 | 1.60E+01 | 4.93E-44 | 6.75E-42 |
| HMMR | Up | 1.50E+00 | 2.48E+00 | 1.59E+01 | 1.04E-43 | 1.41E-41 |
| SPC24 | Up | 1.56E+00 | 3.16E+00 | 1.59E+01 | 1.18E-43 | 1.58E-41 |
| KPNA2 | Up | 1.35E+00 | 5.40E+00 | 1.59E+01 | 1.18E-43 | 1.58E-41 |
| E2F1 | Up | 1.87E+00 | 3.94E+00 | 1.58E+01 | 1.62E-43 | 2.15E-41 |
| DTYMK | Up | 1.08E+00 | 4.69E+00 | 1.58E+01 | 1.97E-43 | 2.55E-41 |
| MCM5 | Up | 1.30E+00 | 4.23E+00 | 1.58E+01 | 2.02E-43 | 2.59E-41 |
| CKS2 | Up | 1.41E+00 | 5.93E+00 | 1.58E+01 | 2.03E-43 | 2.59E-41 |
| RNASEH2A | Up | 1.30E+00 | 4.51E+00 | 1.58E+01 | 2.18E-43 | 2.75E-41 |
| HMGB2 | Up | 1.40E+00 | 5.17E+00 | 1.58E+01 | 2.50E-43 | 3.14E-41 |
| CENPK | Up | 1.06E+00 | 1.29E+00 | 1.58E+01 | 3.39E-43 | 4.22E-41 |
| PIF1 | Up | 1.08E+00 | 1.24E+00 | 1.58E+01 | 3.42E-43 | 4.22E-41 |
| ECT2 | Up | 1.56E+00 | 2.55E+00 | 1.56E+01 | 1.02E-42 | 1.25E-40 |
| TONSL | Up | 1.12E+00 | 2.61E+00 | 1.56E+01 | 1.32E-42 | 1.60E-40 |
| SGO2 | Up | 1.07E+00 | 1.42E+00 | 1.56E+01 | 2.39E-42 | 2.89E-40 |
| KIF15 | Up | 1.23E+00 | 1.32E+00 | 1.55E+01 | 2.43E-42 | 2.91E-40 |
| CHAF1A | Up | 1.12E+00 | 3.37E+00 | 1.55E+01 | 2.46E-42 | 2.93E-40 |
| CDC7 | Up | 1.22E+00 | 1.88E+00 | 1.55E+01 | 2.65E-42 | 3.13E-40 |
| STIL | Up | 1.02E+00 | 1.30E+00 | 1.55E+01 | 5.18E-42 | 6.09E-40 |
| WDR76 | Up | 1.33E+00 | 2.13E+00 | 1.54E+01 | 1.04E-41 | 1.20E-39 |
| UBE2S | Up | 1.33E+00 | 3.67E+00 | 1.54E+01 | 1.48E-41 | 1.70E-39 |
| TRAIP | Up | 1.14E+00 | 1.84E+00 | 1.53E+01 | 2.38E-41 | 2.70E-39 |
| BRCA1 | Up | 1.02E+00 | 1.64E+00 | 1.53E+01 | 2.62E-41 | 2.96E-39 |
| NASP | Up | 1.01E+00 | 4.25E+00 | 1.53E+01 | 3.49E-41 | 3.90E-39 |
| CDCA2 | Up | 1.13E+00 | 1.23E+00 | 1.52E+01 | 8.07E-41 | 8.85E-39 |
| SPDL1 | Up | 1.03E+00 | 2.12E+00 | 1.52E+01 | 9.82E-41 | 1.07E-38 |
| RECQL4 | Up | 1.46E+00 | 3.41E+00 | 1.51E+01 | 1.26E-40 | 1.35E-38 |
| SPATS2 | Up | 1.05E+00 | 2.80E+00 | 1.51E+01 | 1.39E-40 | 1.49E-38 |
| CENPW | Up | 1.58E+00 | 3.98E+00 | 1.51E+01 | 1.77E-40 | 1.86E-38 |
| RBL1 | Up | 1.02E+00 | 1.70E+00 | 1.51E+01 | 2.11E-40 | 2.19E-38 |
| PBK | Up | 1.59E+00 | 2.63E+00 | 1.50E+01 | 5.53E-40 | 5.69E-38 |
| MXD3 | Up | 1.05E+00 | 2.44E+00 | 1.49E+01 | 9.12E-40 | 9.27E-38 |
| CENPO | Up | 1.06E+00 | 1.89E+00 | 1.49E+01 | 1.42E-39 | 1.43E-37 |
| E2F8 | Up | 1.23E+00 | 1.44E+00 | 1.48E+01 | 1.85E-39 | 1.85E-37 |
| MTFR2 | Up | 1.04E+00 | 1.29E+00 | 1.48E+01 | 1.91E-39 | 1.90E-37 |
| RAD51AP1 | Up | 1.34E+00 | 2.11E+00 | 1.48E+01 | 2.03E-39 | 2.01E-37 |
| NRM | Up | 1.44E+00 | 3.85E+00 | 1.48E+01 | 2.31E-39 | 2.28E-37 |
| C17orf53 | Up | 1.09E+00 | 1.73E+00 | 1.47E+01 | 4.62E-39 | 4.53E-37 |
| WDHD1 | Up | 1.00E+00 | 1.38E+00 | 1.47E+01 | 4.67E-39 | 4.56E-37 |
| CHTF18 | Up | 1.06E+00 | 2.34E+00 | 1.47E+01 | 5.15E-39 | 4.99E-37 |
| CDCA4 | Up | 1.14E+00 | 2.80E+00 | 1.47E+01 | 1.10E-38 | 1.05E-36 |
| DSN1 | Up | 1.05E+00 | 3.99E+00 | 1.46E+01 | 1.28E-38 | 1.22E-36 |
| NEIL3 | Up | 1.14E+00 | 1.20E+00 | 1.46E+01 | 1.44E-38 | 1.37E-36 |
| NUDT1 | Up | 1.19E+00 | 3.53E+00 | 1.46E+01 | 1.47E-38 | 1.38E-36 |
| SPAG5 | Up | 1.37E+00 | 3.58E+00 | 1.46E+01 | 2.26E-38 | 2.10E-36 |
| ASPM | Up | 1.43E+00 | 2.21E+00 | 1.45E+01 | 4.29E-38 | 3.97E-36 |
| HELLS | Up | 1.08E+00 | 1.41E+00 | 1.44E+01 | 8.50E-38 | 7.82E-36 |
| NCAPD2 | Up | 1.28E+00 | 3.47E+00 | 1.44E+01 | 9.42E-38 | 8.62E-36 |
| RHNO1 | Up | 1.05E+00 | 3.55E+00 | 1.44E+01 | 1.79E-37 | 1.63E-35 |
| CEP131 | Up | 1.02E+00 | 3.26E+00 | 1.42E+01 | 4.83E-37 | 4.28E-35 |
| NCAPG2 | Up | 1.11E+00 | 2.35E+00 | 1.41E+01 | 1.83E-36 | 1.56E-34 |
| DNMT1 | Up | 1.23E+00 | 3.32E+00 | 1.41E+01 | 2.00E-36 | 1.70E-34 |
| MIS18A | Up | 1.04E+00 | 3.50E+00 | 1.40E+01 | 7.16E-36 | 5.96E-34 |
| FAM111B | Up | 1.35E+00 | 2.28E+00 | 1.39E+01 | 7.66E-36 | 6.28E-34 |
| ALYREF | Up | 1.04E+00 | 5.93E+00 | 1.37E+01 | 5.20E-35 | 4.20E-33 |
| GAS2L3 | Up | 1.04E+00 | 1.53E+00 | 1.37E+01 | 6.04E-35 | 4.86E-33 |
| KNTC1 | Up | 1.11E+00 | 1.79E+00 | 1.37E+01 | 7.12E-35 | 5.67E-33 |
| PAQR4 | Up | 1.40E+00 | 2.54E+00 | 1.37E+01 | 9.33E-35 | 7.36E-33 |
| LMNB2 | Up | 1.19E+00 | 3.57E+00 | 1.36E+01 | 1.75E-34 | 1.35E-32 |
| WDR34 | Up | 1.14E+00 | 5.15E+00 | 1.36E+01 | 1.96E-34 | 1.50E-32 |
| NUP210 | Up | 1.12E+00 | 4.10E+00 | 1.35E+01 | 6.19E-34 | 4.64E-32 |
| KIF14 | Up | 1.00E+00 | 1.28E+00 | 1.34E+01 | 9.15E-34 | 6.80E-32 |
| FANCE | Up | 1.07E+00 | 2.14E+00 | 1.34E+01 | 9.37E-34 | 6.93E-32 |
| TMEM106C | Up | 1.12E+00 | 5.44E+00 | 1.33E+01 | 1.84E-33 | 1.32E-31 |
| MSH2 | Up | 1.02E+00 | 3.29E+00 | 1.33E+01 | 2.07E-33 | 1.46E-31 |
| MND1 | Up | 1.09E+00 | 2.22E+00 | 1.33E+01 | 2.09E-33 | 1.48E-31 |
| CDK2 | Up | 1.04E+00 | 3.62E+00 | 1.31E+01 | 2.20E-32 | 1.47E-30 |
| CBX1 | Up | 1.00E+00 | 4.66E+00 | 1.30E+01 | 2.72E-32 | 1.79E-30 |
| CDKN2C | Up | 1.31E+00 | 3.57E+00 | 1.30E+01 | 2.89E-32 | 1.90E-30 |
| SPINDOC | Up | 1.14E+00 | 2.97E+00 | 1.30E+01 | 3.00E-32 | 1.96E-30 |
| RIBC2 | Up | 1.18E+00 | 1.20E+00 | 1.29E+01 | 7.18E-32 | 4.54E-30 |
| PSRC1 | Up | 1.14E+00 | 2.29E+00 | 1.29E+01 | 8.80E-32 | 5.50E-30 |
| CDC25B | Up | 1.21E+00 | 4.77E+00 | 1.29E+01 | 8.91E-32 | 5.55E-30 |
| PRIM1 | Up | 1.11E+00 | 3.13E+00 | 1.27E+01 | 4.63E-31 | 2.75E-29 |
| CCNE1 | Up | 1.55E+00 | 2.10E+00 | 1.27E+01 | 6.45E-31 | 3.75E-29 |
| AURKA | Up | 1.29E+00 | 3.83E+00 | 1.27E+01 | 7.51E-31 | 4.32E-29 |
| CDK4 | Up | 1.09E+00 | 4.99E+00 | 1.26E+01 | 1.44E-30 | 8.05E-29 |
| SAPCD2 | Up | 1.20E+00 | 1.33E+00 | 1.25E+01 | 3.75E-30 | 2.03E-28 |
| RCC2 | Up | 1.06E+00 | 4.78E+00 | 1.24E+01 | 9.95E-30 | 5.24E-28 |
| CLIC1 | Up | 1.15E+00 | 7.68E+00 | 1.24E+01 | 1.22E-29 | 6.36E-28 |
| ESPL1 | Up | 1.13E+00 | 1.84E+00 | 1.23E+01 | 1.98E-29 | 1.01E-27 |
| CCDC34 | Up | 1.05E+00 | 2.93E+00 | 1.21E+01 | 9.54E-29 | 4.58E-27 |
| JPT1 | Up | 1.06E+00 | 4.96E+00 | 1.21E+01 | 1.51E-28 | 7.10E-27 |
| TUBA1C | Up | 1.12E+00 | 4.29E+00 | 1.20E+01 | 2.34E-28 | 1.07E-26 |
| TUBA1B | Up | 1.38E+00 | 5.80E+00 | 1.20E+01 | 2.37E-28 | 1.08E-26 |
| G6PD | Up | 1.63E+00 | 4.22E+00 | 1.19E+01 | 7.64E-28 | 3.28E-26 |
| C19orf48 | Up | 1.05E+00 | 5.16E+00 | 1.18E+01 | 1.55E-27 | 6.39E-26 |
| PAFAH1B3 | Up | 1.48E+00 | 4.49E+00 | 1.17E+01 | 3.91E-27 | 1.55E-25 |
| PKM | Up | 1.71E+00 | 4.95E+00 | 1.17E+01 | 5.08E-27 | 1.98E-25 |
| INCENP | Up | 1.02E+00 | 2.51E+00 | 1.16E+01 | 8.33E-27 | 3.19E-25 |
| BAK1 | Up | 1.05E+00 | 4.06E+00 | 1.16E+01 | 1.32E-26 | 4.93E-25 |
| HMGA1 | Up | 1.35E+00 | 6.15E+00 | 1.14E+01 | 3.93E-26 | 1.39E-24 |
| IQGAP3 | Up | 1.24E+00 | 2.64E+00 | 1.14E+01 | 3.98E-26 | 1.40E-24 |
| PLXNA1 | Up | 1.03E+00 | 2.40E+00 | 1.13E+01 | 1.47E-25 | 4.78E-24 |
| C12orf75 | Up | 1.74E+00 | 2.83E+00 | 1.11E+01 | 5.91E-25 | 1.77E-23 |
| PSPH | Up | 1.11E+00 | 4.23E+00 | 1.11E+01 | 8.66E-25 | 2.55E-23 |
| PIMREG | Up | 1.04E+00 | 1.06E+00 | 1.10E+01 | 1.50E-24 | 4.28E-23 |
| LPCAT1 | Up | 1.23E+00 | 3.70E+00 | 1.09E+01 | 4.10E-24 | 1.11E-22 |
| ALDOA | Up | 1.10E+00 | 7.11E+00 | 1.08E+01 | 9.83E-24 | 2.54E-22 |
| MMD | Up | 1.12E+00 | 3.63E+00 | 1.07E+01 | 2.36E-23 | 5.90E-22 |
| KIAA1522 | Up | 1.02E+00 | 4.85E+00 | 1.05E+01 | 1.19E-22 | 2.66E-21 |
| ZBTB12 | Up | 1.02E+00 | 2.25E+00 | 1.04E+01 | 2.24E-22 | 4.83E-21 |
| SMC4 | Up | 1.13E+00 | 2.44E+00 | 1.03E+01 | 3.62E-22 | 7.56E-21 |
| ARHGEF2 | Up | 1.02E+00 | 2.95E+00 | 1.03E+01 | 4.42E-22 | 9.11E-21 |
| TYRO3 | Up | 1.09E+00 | 1.41E+00 | 1.02E+01 | 7.79E-22 | 1.54E-20 |
| CDCA7L | Up | 1.06E+00 | 2.05E+00 | 1.01E+01 | 1.93E-21 | 3.60E-20 |
| HILPDA | Up | 1.01E+00 | 2.73E+00 | 1.01E+01 | 2.03E-21 | 3.76E-20 |
| IKBKE | Up | 1.05E+00 | 2.23E+00 | 1.01E+01 | 3.03E-21 | 5.51E-20 |
| RASSF3 | Up | 1.11E+00 | 3.95E+00 | 1.01E+01 | 3.06E-21 | 5.55E-20 |
| ATP1A1 | Up | 1.02E+00 | 7.19E+00 | 1.00E+01 | 3.69E-21 | 6.62E-20 |
| MARCKSL1 | Up | 1.21E+00 | 6.29E+00 | 9.97E+00 | 6.84E-21 | 1.18E-19 |
| CDKN2A | Up | 1.34E+00 | 2.85E+00 | 9.95E+00 | 7.84E-21 | 1.34E-19 |
| GMNN | Up | 1.05E+00 | 5.10E+00 | 9.86E+00 | 1.54E-20 | 2.51E-19 |
| ATP1B3 | Up | 1.09E+00 | 4.61E+00 | 9.84E+00 | 1.87E-20 | 3.00E-19 |
| PITX1 | Up | 1.49E+00 | 1.46E+00 | 9.81E+00 | 2.44E-20 | 3.86E-19 |
| CA9 | Up | 2.15E+00 | 1.74E+00 | 9.77E+00 | 3.20E-20 | 4.96E-19 |
| IGSF3 | Up | 1.25E+00 | 2.18E+00 | 9.73E+00 | 4.51E-20 | 6.85E-19 |
| MFSD10 | Up | 1.01E+00 | 4.13E+00 | 9.72E+00 | 4.88E-20 | 7.38E-19 |
| ASRGL1 | Up | 1.04E+00 | 1.92E+00 | 9.71E+00 | 5.07E-20 | 7.62E-19 |
| SLC16A3 | Up | 1.24E+00 | 2.31E+00 | 9.69E+00 | 6.17E-20 | 9.15E-19 |
| SPHK1 | Up | 1.70E+00 | 2.46E+00 | 9.68E+00 | 6.42E-20 | 9.50E-19 |
| FAM83D | Up | 1.12E+00 | 3.47E+00 | 9.63E+00 | 9.92E-20 | 1.43E-18 |
| PLP2 | Up | 1.37E+00 | 5.68E+00 | 9.62E+00 | 1.02E-19 | 1.47E-18 |
| P3H4 | Up | 1.03E+00 | 3.05E+00 | 9.58E+00 | 1.40E-19 | 1.98E-18 |
| MARCKS | Up | 1.08E+00 | 5.43E+00 | 9.53E+00 | 2.12E-19 | 2.92E-18 |
| SLC1A5 | Up | 1.42E+00 | 3.61E+00 | 9.52E+00 | 2.31E-19 | 3.16E-18 |
| NXPH4 | Up | 1.52E+00 | 1.73E+00 | 9.47E+00 | 3.40E-19 | 4.52E-18 |
| IL4I1 | Up | 1.16E+00 | 1.80E+00 | 9.44E+00 | 4.19E-19 | 5.52E-18 |
| NFE2L3 | Up | 1.01E+00 | 2.20E+00 | 9.42E+00 | 4.84E-19 | 6.30E-18 |
| LRRC1 | Up | 1.17E+00 | 2.33E+00 | 9.41E+00 | 5.38E-19 | 6.96E-18 |
| ANXA5 | Up | 1.05E+00 | 6.92E+00 | 9.35E+00 | 8.66E-19 | 1.08E-17 |
| TREM2 | Up | 1.29E+00 | 3.14E+00 | 9.29E+00 | 1.31E-18 | 1.60E-17 |
| KCTD17 | Up | 1.30E+00 | 3.00E+00 | 9.26E+00 | 1.62E-18 | 1.96E-17 |
| SNX7 | Up | 1.17E+00 | 3.69E+00 | 9.24E+00 | 1.95E-18 | 2.32E-17 |
| NT5DC2 | Up | 1.23E+00 | 3.34E+00 | 9.14E+00 | 4.23E-18 | 4.77E-17 |
| ELOVL7 | Up | 1.41E+00 | 1.80E+00 | 9.08E+00 | 6.33E-18 | 7.00E-17 |
| HOMER3 | Up | 1.02E+00 | 2.97E+00 | 9.01E+00 | 1.08E-17 | 1.15E-16 |
| TMSB10 | Up | 1.23E+00 | 1.07E+01 | 9.01E+00 | 1.13E-17 | 1.20E-16 |
| CTSC | Up | 1.05E+00 | 3.85E+00 | 8.96E+00 | 1.59E-17 | 1.67E-16 |
| TMEM51 | Up | 1.16E+00 | 2.73E+00 | 8.93E+00 | 2.02E-17 | 2.09E-16 |
| IMPDH1 | Up | 1.08E+00 | 2.96E+00 | 8.91E+00 | 2.27E-17 | 2.32E-16 |
| PYCARD | Up | 1.24E+00 | 3.98E+00 | 8.87E+00 | 3.12E-17 | 3.14E-16 |
| DSG2 | Up | 1.36E+00 | 3.44E+00 | 8.78E+00 | 6.25E-17 | 6.01E-16 |
| NRSN2 | Up | 1.34E+00 | 2.97E+00 | 8.76E+00 | 6.79E-17 | 6.50E-16 |
| S100A11 | Up | 1.30E+00 | 7.29E+00 | 8.76E+00 | 6.98E-17 | 6.67E-16 |
| CAPG | Up | 1.33E+00 | 4.38E+00 | 8.75E+00 | 7.32E-17 | 6.96E-16 |
| COL9A2 | Up | 1.04E+00 | 1.33E+00 | 8.74E+00 | 8.10E-17 | 7.66E-16 |
| INAVA | Up | 1.20E+00 | 1.46E+00 | 8.64E+00 | 1.64E-16 | 1.49E-15 |
| ITPKA | Up | 1.19E+00 | 2.96E+00 | 8.61E+00 | 2.16E-16 | 1.93E-15 |
| BLVRA | Up | 1.04E+00 | 4.58E+00 | 8.58E+00 | 2.53E-16 | 2.24E-15 |
| IFI27L2 | Up | 1.04E+00 | 3.16E+00 | 8.57E+00 | 2.75E-16 | 2.42E-15 |
| ABCC1 | Up | 1.01E+00 | 2.26E+00 | 8.54E+00 | 3.43E-16 | 2.96E-15 |
| PAQR5 | Up | 1.27E+00 | 1.87E+00 | 8.53E+00 | 3.63E-16 | 3.12E-15 |
| ENO2 | Up | 1.03E+00 | 1.56E+00 | 8.53E+00 | 3.74E-16 | 3.21E-15 |
| TMEM132A | Up | 1.12E+00 | 1.89E+00 | 8.52E+00 | 4.11E-16 | 3.50E-15 |
| UAP1L1 | Up | 1.04E+00 | 1.79E+00 | 8.48E+00 | 5.48E-16 | 4.56E-15 |
| CDCA7 | Up | 1.13E+00 | 1.29E+00 | 8.45E+00 | 6.79E-16 | 5.58E-15 |
| EGLN3 | Up | 1.07E+00 | 1.56E+00 | 8.44E+00 | 7.03E-16 | 5.78E-15 |
| TEAD2 | Up | 1.08E+00 | 3.82E+00 | 8.44E+00 | 7.33E-16 | 6.00E-15 |
| ITPR3 | Up | 1.12E+00 | 1.46E+00 | 8.38E+00 | 1.07E-15 | 8.56E-15 |
| FAM241B | Up | 1.04E+00 | 2.81E+00 | 8.38E+00 | 1.12E-15 | 8.99E-15 |
| LAPTM4B | Up | 1.24E+00 | 6.05E+00 | 8.36E+00 | 1.27E-15 | 1.00E-14 |
| GAL3ST1 | Up | 1.61E+00 | 2.66E+00 | 8.34E+00 | 1.50E-15 | 1.18E-14 |
| FOXJ1 | Up | 1.13E+00 | 9.27E-01 | 8.31E+00 | 1.88E-15 | 1.45E-14 |
| NDRG1 | Up | 1.10E+00 | 5.36E+00 | 8.30E+00 | 2.00E-15 | 1.54E-14 |
| MELTF | Up | 1.04E+00 | 1.54E+00 | 8.29E+00 | 2.05E-15 | 1.57E-14 |
| DBN1 | Up | 1.15E+00 | 3.30E+00 | 8.29E+00 | 2.08E-15 | 1.59E-14 |
| DDR1 | Up | 1.29E+00 | 2.82E+00 | 8.23E+00 | 3.11E-15 | 2.33E-14 |
| GCNT3 | Up | 1.08E+00 | 1.28E+00 | 8.20E+00 | 4.04E-15 | 2.98E-14 |
| MMP14 | Up | 1.06E+00 | 5.16E+00 | 8.09E+00 | 8.70E-15 | 6.15E-14 |
| VEGFB | Up | 1.27E+00 | 5.60E+00 | 8.07E+00 | 9.84E-15 | 6.90E-14 |
| PLEKHB1 | Up | 1.12E+00 | 1.11E+00 | 8.00E+00 | 1.62E-14 | 1.10E-13 |
| CTNND2 | Up | 1.28E+00 | 1.24E+00 | 7.85E+00 | 4.54E-14 | 2.88E-13 |
| SLC38A1 | Up | 1.18E+00 | 2.87E+00 | 7.84E+00 | 4.82E-14 | 3.03E-13 |
| PLBD1 | Up | 1.12E+00 | 2.38E+00 | 7.78E+00 | 7.27E-14 | 4.44E-13 |
| MMP9 | Up | 1.43E+00 | 3.17E+00 | 7.74E+00 | 9.40E-14 | 5.66E-13 |
| APOBEC3B | Up | 1.02E+00 | 2.15E+00 | 7.71E+00 | 1.20E-13 | 7.10E-13 |
| MDK | Up | 1.40E+00 | 6.68E+00 | 7.69E+00 | 1.33E-13 | 7.78E-13 |
| SPATC1L | Up | 1.01E+00 | 2.38E+00 | 7.67E+00 | 1.49E-13 | 8.68E-13 |
| GNAZ | Up | 1.11E+00 | 2.55E+00 | 7.66E+00 | 1.58E-13 | 9.16E-13 |
| NEURL3 | Up | 1.08E+00 | 2.04E+00 | 7.64E+00 | 1.82E-13 | 1.04E-12 |
| MMP12 | Up | 1.09E+00 | 1.02E+00 | 7.59E+00 | 2.56E-13 | 1.44E-12 |
| DUSP9 | Up | 1.50E+00 | 3.03E+00 | 7.52E+00 | 4.10E-13 | 2.23E-12 |
| SFN | Up | 1.75E+00 | 4.43E+00 | 7.37E+00 | 1.11E-12 | 5.65E-12 |
| SOX4 | Up | 1.16E+00 | 3.41E+00 | 7.31E+00 | 1.65E-12 | 8.19E-12 |
| ASNS | Up | 1.01E+00 | 2.20E+00 | 7.27E+00 | 2.10E-12 | 1.03E-11 |
| RENBP | Up | 1.07E+00 | 3.22E+00 | 7.17E+00 | 4.09E-12 | 1.92E-11 |
| PAPLN | Up | 1.04E+00 | 1.81E+00 | 7.10E+00 | 6.38E-12 | 2.91E-11 |
| TRNP1 | Up | 1.40E+00 | 3.62E+00 | 7.10E+00 | 6.49E-12 | 2.96E-11 |
| EPS8L3 | Up | 1.35E+00 | 2.84E+00 | 7.05E+00 | 8.56E-12 | 3.83E-11 |
| CLGN | Up | 1.22E+00 | 2.71E+00 | 6.99E+00 | 1.25E-11 | 5.46E-11 |
| LYPD1 | Up | 1.14E+00 | 1.98E+00 | 6.99E+00 | 1.28E-11 | 5.56E-11 |
| WNK2 | Up | 1.01E+00 | 1.10E+00 | 6.92E+00 | 2.01E-11 | 8.52E-11 |
| SEL1L3 | Up | 1.11E+00 | 2.93E+00 | 6.90E+00 | 2.26E-11 | 9.52E-11 |
| C6orf223 | Up | 1.06E+00 | 9.97E-01 | 6.81E+00 | 3.97E-11 | 1.61E-10 |
| S100A6 | Up | 1.19E+00 | 6.80E+00 | 6.80E+00 | 4.31E-11 | 1.74E-10 |
| PFKP | Up | 1.04E+00 | 2.56E+00 | 6.79E+00 | 4.35E-11 | 1.76E-10 |
| KIF12 | Up | 1.06E+00 | 3.88E+00 | 6.76E+00 | 5.36E-11 | 2.13E-10 |
| RGS2 | Up | 1.07E+00 | 3.73E+00 | 6.75E+00 | 5.72E-11 | 2.27E-10 |
| RGS1 | Up | 1.08E+00 | 2.92E+00 | 6.70E+00 | 7.68E-11 | 2.99E-10 |
| TCEAL9 | Up | 1.02E+00 | 5.23E+00 | 6.63E+00 | 1.17E-10 | 4.42E-10 |
| SPP1 | Up | 2.20E+00 | 6.76E+00 | 6.53E+00 | 2.18E-10 | 7.99E-10 |
| CTHRC1 | Up | 1.13E+00 | 2.59E+00 | 6.50E+00 | 2.65E-10 | 9.57E-10 |
| C15orf48 | Up | 1.18E+00 | 2.55E+00 | 6.49E+00 | 2.78E-10 | 1.00E-09 |
| AFP | Up | 2.15E+00 | 3.99E+00 | 6.37E+00 | 5.73E-10 | 1.98E-09 |
| ANXA13 | Up | 1.27E+00 | 3.06E+00 | 6.35E+00 | 6.23E-10 | 2.14E-09 |
| VIL1 | Up | 1.30E+00 | 3.50E+00 | 6.35E+00 | 6.25E-10 | 2.14E-09 |
| HKDC1 | Up | 1.15E+00 | 3.91E+00 | 6.32E+00 | 7.70E-10 | 2.61E-09 |
| SLC6A8 | Up | 1.12E+00 | 2.81E+00 | 6.31E+00 | 8.16E-10 | 2.76E-09 |
| MAPK13 | Up | 1.01E+00 | 2.14E+00 | 6.26E+00 | 1.07E-09 | 3.56E-09 |
| UBD | Up | 1.23E+00 | 6.66E+00 | 6.21E+00 | 1.46E-09 | 4.73E-09 |
| H2AFY2 | Up | 1.08E+00 | 3.67E+00 | 6.14E+00 | 2.14E-09 | 6.81E-09 |
| SPINT1 | Up | 1.39E+00 | 2.69E+00 | 6.10E+00 | 2.74E-09 | 8.55E-09 |
| SLC29A4 | Up | 1.12E+00 | 2.34E+00 | 6.06E+00 | 3.27E-09 | 1.01E-08 |
| SUSD4 | Up | 1.13E+00 | 2.37E+00 | 6.05E+00 | 3.49E-09 | 1.07E-08 |
| PHGDH | Up | 1.04E+00 | 4.34E+00 | 6.03E+00 | 3.87E-09 | 1.18E-08 |
| GTSF1 | Up | 1.13E+00 | 1.20E+00 | 6.01E+00 | 4.42E-09 | 1.34E-08 |
| PYCR1 | Up | 1.23E+00 | 3.18E+00 | 5.99E+00 | 5.02E-09 | 1.51E-08 |
| CD24 | Up | 1.51E+00 | 5.42E+00 | 5.88E+00 | 8.95E-09 | 2.60E-08 |
| HIST3H2A | Up | 1.04E+00 | 1.75E+00 | 5.87E+00 | 9.66E-09 | 2.79E-08 |
| CLDN4 | Up | 1.39E+00 | 2.75E+00 | 5.86E+00 | 1.02E-08 | 2.93E-08 |
| MISP | Up | 1.12E+00 | 1.30E+00 | 5.83E+00 | 1.18E-08 | 3.38E-08 |
| S100A14 | Up | 1.32E+00 | 4.10E+00 | 5.67E+00 | 2.80E-08 | 7.62E-08 |
| PRAME | Up | 1.10E+00 | 1.19E+00 | 5.67E+00 | 2.90E-08 | 7.87E-08 |
| PLPP2 | Up | 1.09E+00 | 2.00E+00 | 5.44E+00 | 9.52E-08 | 2.41E-07 |
| SSX1 | Up | 1.39E+00 | 1.90E+00 | 5.39E+00 | 1.23E-07 | 3.06E-07 |
| MAGEA6 | Up | 1.26E+00 | 1.44E+00 | 5.37E+00 | 1.42E-07 | 3.51E-07 |
| KRT19 | Up | 1.32E+00 | 2.24E+00 | 5.31E+00 | 1.93E-07 | 4.69E-07 |
| CSAG1 | Up | 1.15E+00 | 1.37E+00 | 5.23E+00 | 2.88E-07 | 6.85E-07 |
| MMP7 | Up | 1.18E+00 | 2.10E+00 | 5.22E+00 | 3.02E-07 | 7.18E-07 |
| ETV4 | Up | 1.02E+00 | 2.97E+00 | 5.18E+00 | 3.72E-07 | 8.73E-07 |
| UCHL1 | Up | 1.00E+00 | 1.58E+00 | 5.15E+00 | 4.21E-07 | 9.81E-07 |
| CTAG2 | Up | 1.18E+00 | 1.39E+00 | 5.11E+00 | 5.27E-07 | 1.21E-06 |
| SPINT2 | Up | 1.03E+00 | 2.61E+00 | 5.10E+00 | 5.43E-07 | 1.24E-06 |
| MAGEA3 | Up | 1.25E+00 | 1.61E+00 | 5.08E+00 | 5.90E-07 | 1.35E-06 |
| NPTX2 | Up | 1.02E+00 | 1.46E+00 | 5.06E+00 | 6.45E-07 | 1.46E-06 |
| AGR2 | Up | 1.14E+00 | 1.31E+00 | 5.06E+00 | 6.47E-07 | 1.47E-06 |
| BEX2 | Up | 1.13E+00 | 2.63E+00 | 5.06E+00 | 6.76E-07 | 1.53E-06 |
| MSC | Up | 1.02E+00 | 2.62E+00 | 4.94E+00 | 1.17E-06 | 2.58E-06 |
| COX7B2 | Up | 1.27E+00 | 2.00E+00 | 4.91E+00 | 1.38E-06 | 3.01E-06 |
| CXCL1 | Up | 1.08E+00 | 2.39E+00 | 4.77E+00 | 2.61E-06 | 5.51E-06 |
| SCGN | Up | 1.01E+00 | 3.10E+00 | 4.68E+00 | 4.08E-06 | 8.40E-06 |
| AP1M2 | Up | 1.11E+00 | 2.55E+00 | 4.59E+00 | 6.15E-06 | 1.24E-05 |
| SPINK1 | Up | 1.72E+00 | 6.30E+00 | 4.58E+00 | 6.43E-06 | 1.29E-05 |
| FDCSP | Up | 1.02E+00 | 1.30E+00 | 4.55E+00 | 7.39E-06 | 1.47E-05 |
| NTS | Up | 1.23E+00 | 1.56E+00 | 4.34E+00 | 1.81E-05 | 3.43E-05 |
| DKK1.00 | Up | 1.02E+00 | 1.78E+00 | 4.28E+00 | 2.34E-05 | 4.37E-05 |
| GPC3 | Up | 1.27E+00 | 7.56E+00 | 4.12E+00 | 4.65E-05 | 8.37E-05 |
| EPCAM | Up | 1.23E+00 | 2.74E+00 | 4.09E+00 | 5.37E-05 | 9.58E-05 |
| PEG10 | Up | 1.22E+00 | 3.25E+00 | 3.89E+00 | 1.18E-04 | 2.02E-04 |
| S100P | Up | 1.07E+00 | 3.61E+00 | 3.37E+00 | 8.30E-04 | 1.28E-03 |
| CFHR4 | Down | -1.85E+00 | 3.96E+00 | -9.94E+00 | 8.62E-21 | 1.47E-19 |
| SLC27A5 | Down | -1.73E+00 | 6.04E+00 | -9.56E+00 | 1.71E-19 | 2.39E-18 |
| GLYATL1 | Down | -1.52E+00 | 4.41E+00 | -9.08E+00 | 6.41E-18 | 7.07E-17 |
| DNASE1L3 | Down | -1.19E+00 | 3.03E+00 | -8.94E+00 | 1.86E-17 | 1.93E-16 |
| HPD | Down | -2.56E+00 | 8.55E+00 | -8.75E+00 | 7.60E-17 | 7.21E-16 |
| SLC10A1 | Down | -2.29E+00 | 5.72E+00 | -8.68E+00 | 1.23E-16 | 1.13E-15 |
| CDO1 | Down | -1.49E+00 | 6.97E+00 | -8.59E+00 | 2.52E-16 | 2.23E-15 |
| TAT | Down | -2.37E+00 | 6.73E+00 | -8.49E+00 | 4.96E-16 | 4.17E-15 |
| GYS2 | Down | -1.70E+00 | 3.90E+00 | -8.43E+00 | 7.61E-16 | 6.20E-15 |
| ADH1B | Down | -1.94E+00 | 7.85E+00 | -8.40E+00 | 9.83E-16 | 7.90E-15 |
| HPR | Down | -1.78E+00 | 7.82E+00 | -8.36E+00 | 1.23E-15 | 9.76E-15 |
| RBP4 | Down | -1.25E+00 | 1.25E+01 | -8.32E+00 | 1.71E-15 | 1.33E-14 |
| SLC13A5 | Down | -1.66E+00 | 5.20E+00 | -8.30E+00 | 1.91E-15 | 1.48E-14 |
| PFKFB1 | Down | -1.31E+00 | 3.66E+00 | -8.30E+00 | 2.01E-15 | 1.55E-14 |
| PCK1 | Down | -1.99E+00 | 6.50E+00 | -8.08E+00 | 9.05E-15 | 6.38E-14 |
| ASPDH | Down | -1.47E+00 | 5.01E+00 | -7.80E+00 | 6.20E-14 | 3.83E-13 |
| MASP2 | Down | -1.49E+00 | 5.98E+00 | -7.79E+00 | 6.79E-14 | 4.17E-13 |
| ADH4 | Down | -2.34E+00 | 7.32E+00 | -7.75E+00 | 8.70E-14 | 5.25E-13 |
| AQP9 | Down | -1.93E+00 | 6.95E+00 | -7.75E+00 | 8.96E-14 | 5.40E-13 |
| ACSM2A | Down | -1.36E+00 | 5.12E+00 | -7.73E+00 | 1.02E-13 | 6.08E-13 |
| HP | Down | -1.76E+00 | 1.06E+01 | -7.66E+00 | 1.61E-13 | 9.31E-13 |
| TTC36 | Down | -1.73E+00 | 3.25E+00 | -7.66E+00 | 1.64E-13 | 9.47E-13 |
| A1BG | Down | -1.29E+00 | 5.16E+00 | -7.53E+00 | 4.00E-13 | 2.19E-12 |
| CYP8B1 | Down | -2.14E+00 | 5.59E+00 | -7.50E+00 | 4.85E-13 | 2.60E-12 |
| F9 | Down | -1.77E+00 | 6.19E+00 | -7.48E+00 | 5.36E-13 | 2.87E-12 |
| SLC6A12 | Down | -1.13E+00 | 3.88E+00 | -7.47E+00 | 5.98E-13 | 3.18E-12 |
| SERPINC1 | Down | -1.74E+00 | 1.10E+01 | -7.40E+00 | 9.20E-13 | 4.76E-12 |
| GLYAT | Down | -1.80E+00 | 4.33E+00 | -7.37E+00 | 1.13E-12 | 5.78E-12 |
| ABCB4 | Down | -1.22E+00 | 4.66E+00 | -7.36E+00 | 1.22E-12 | 6.18E-12 |
| SLC25A47 | Down | -2.08E+00 | 4.97E+00 | -7.34E+00 | 1.34E-12 | 6.74E-12 |
| AOX1 | Down | -1.56E+00 | 7.26E+00 | -7.30E+00 | 1.80E-12 | 8.92E-12 |
| MAT1A | Down | -1.13E+00 | 8.57E+00 | -7.27E+00 | 2.16E-12 | 1.05E-11 |
| C8A | Down | -1.22E+00 | 7.10E+00 | -7.17E+00 | 4.17E-12 | 1.96E-11 |
| AMDHD1 | Down | -1.01E+00 | 5.34E+00 | -7.16E+00 | 4.31E-12 | 2.02E-11 |
| C6 | Down | -1.33E+00 | 6.14E+00 | -7.09E+00 | 7.02E-12 | 3.18E-11 |
| CYP4A22 | Down | -1.28E+00 | 4.81E+00 | -7.04E+00 | 9.62E-12 | 4.28E-11 |
| AR | Down | -1.06E+00 | 3.43E+00 | -7.01E+00 | 1.11E-11 | 4.90E-11 |
| HPX | Down | -1.33E+00 | 9.81E+00 | -6.98E+00 | 1.33E-11 | 5.77E-11 |
| APOC3 | Down | -1.53E+00 | 1.25E+01 | -6.97E+00 | 1.42E-11 | 6.13E-11 |
| FMO3 | Down | -1.40E+00 | 7.04E+00 | -6.97E+00 | 1.43E-11 | 6.17E-11 |
| G6PC | Down | -1.43E+00 | 7.57E+00 | -6.95E+00 | 1.63E-11 | 6.97E-11 |
| CYP4F2 | Down | -1.35E+00 | 5.36E+00 | -6.85E+00 | 3.05E-11 | 1.26E-10 |
| AGXT | Down | -1.38E+00 | 8.56E+00 | -6.76E+00 | 5.40E-11 | 2.15E-10 |
| CFHR3 | Down | -1.47E+00 | 4.70E+00 | -6.67E+00 | 9.49E-11 | 3.66E-10 |
| LRCOL1 | Down | -1.09E+00 | 1.95E+00 | -6.65E+00 | 1.03E-10 | 3.95E-10 |
| SPP2 | Down | -1.71E+00 | 5.86E+00 | -6.65E+00 | 1.04E-10 | 3.97E-10 |
| HJV | Down | -1.36E+00 | 6.99E+00 | -6.64E+00 | 1.09E-10 | 4.16E-10 |
| CES2 | Down | -1.01E+00 | 7.45E+00 | -6.63E+00 | 1.16E-10 | 4.40E-10 |
| APOA5 | Down | -1.38E+00 | 7.18E+00 | -6.63E+00 | 1.18E-10 | 4.45E-10 |
| F12 | Down | -1.21E+00 | 8.12E+00 | -6.60E+00 | 1.43E-10 | 5.35E-10 |
| HAO1 | Down | -1.17E+00 | 7.29E+00 | -6.59E+00 | 1.48E-10 | 5.54E-10 |
| PLG | Down | -1.26E+00 | 8.18E+00 | -6.58E+00 | 1.56E-10 | 5.82E-10 |
| UGT1A4 | Down | -1.70E+00 | 4.11E+00 | -6.58E+00 | 1.57E-10 | 5.83E-10 |
| CPN2 | Down | -1.13E+00 | 6.99E+00 | -6.57E+00 | 1.71E-10 | 6.34E-10 |
| FBP1 | Down | -1.13E+00 | 7.67E+00 | -6.53E+00 | 2.23E-10 | 8.14E-10 |
| INSIG1 | Down | -1.01E+00 | 7.78E+00 | -6.51E+00 | 2.50E-10 | 9.06E-10 |
| CYP4A11 | Down | -1.31E+00 | 6.64E+00 | -6.47E+00 | 3.06E-10 | 1.09E-09 |
| SEC14L2 | Down | -1.07E+00 | 5.15E+00 | -6.46E+00 | 3.37E-10 | 1.20E-09 |
| NR1I3 | Down | -1.16E+00 | 4.69E+00 | -6.43E+00 | 3.83E-10 | 1.35E-09 |
| CYP3A4 | Down | -2.44E+00 | 5.87E+00 | -6.39E+00 | 4.88E-10 | 1.70E-09 |
| HSD17B6 | Down | -1.34E+00 | 7.94E+00 | -6.38E+00 | 5.22E-10 | 1.81E-09 |
| CCL16 | Down | -1.42E+00 | 5.93E+00 | -6.38E+00 | 5.38E-10 | 1.86E-09 |
| SLC22A1 | Down | -1.85E+00 | 5.59E+00 | -6.37E+00 | 5.54E-10 | 1.92E-09 |
| ACSM2B | Down | -1.10E+00 | 5.64E+00 | -6.37E+00 | 5.70E-10 | 1.96E-09 |
| ELFN1 | Down | -1.07E+00 | 2.75E+00 | -6.35E+00 | 6.17E-10 | 2.12E-09 |
| LECT2 | Down | -1.53E+00 | 5.17E+00 | -6.34E+00 | 6.53E-10 | 2.23E-09 |
| SLC2A2 | Down | -1.19E+00 | 7.41E+00 | -6.34E+00 | 6.73E-10 | 2.30E-09 |
| C4BPA | Down | -1.27E+00 | 9.31E+00 | -6.33E+00 | 7.22E-10 | 2.45E-09 |
| APOF | Down | -1.42E+00 | 5.18E+00 | -6.30E+00 | 8.37E-10 | 2.82E-09 |
| ADH1C | Down | -1.80E+00 | 7.58E+00 | -6.27E+00 | 1.03E-09 | 3.41E-09 |
| RTP3 | Down | -1.38E+00 | 4.66E+00 | -6.26E+00 | 1.09E-09 | 3.59E-09 |
| ITIH3 | Down | -1.04E+00 | 8.91E+00 | -6.22E+00 | 1.34E-09 | 4.37E-09 |
| SAA4 | Down | -1.36E+00 | 7.20E+00 | -6.22E+00 | 1.35E-09 | 4.41E-09 |
| MOGAT2 | Down | -1.20E+00 | 2.94E+00 | -6.21E+00 | 1.44E-09 | 4.67E-09 |
| ANGPTL3 | Down | -1.05E+00 | 7.38E+00 | -6.18E+00 | 1.71E-09 | 5.49E-09 |
| CYP20.00 | Down | -2.21E+00 | 6.79E+00 | -6.17E+00 | 1.78E-09 | 5.70E-09 |
| CYP2A6 | Down | -2.24E+00 | 6.60E+00 | -6.16E+00 | 1.86E-09 | 5.95E-09 |
| DPYS | Down | -1.17E+00 | 6.46E+00 | -6.16E+00 | 1.94E-09 | 6.19E-09 |
| AZGP1 | Down | -1.06E+00 | 8.79E+00 | -6.15E+00 | 1.97E-09 | 6.28E-09 |
| ACSM5 | Down | -1.22E+00 | 4.78E+00 | -6.15E+00 | 2.00E-09 | 6.36E-09 |
| SERPINA11 | Down | -1.30E+00 | 6.88E+00 | -6.13E+00 | 2.28E-09 | 7.20E-09 |
| UROC1 | Down | -1.45E+00 | 3.34E+00 | -6.12E+00 | 2.32E-09 | 7.34E-09 |
| ABCB11 | Down | -1.15E+00 | 3.09E+00 | -6.12E+00 | 2.36E-09 | 7.46E-09 |
| ALDOB | Down | -1.47E+00 | 1.06E+01 | -5.99E+00 | 4.95E-09 | 1.49E-08 |
| AADAC | Down | -1.09E+00 | 7.94E+00 | -5.98E+00 | 5.29E-09 | 1.59E-08 |
| IGFBP1 | Down | -1.31E+00 | 8.83E+00 | -5.97E+00 | 5.58E-09 | 1.67E-08 |
| SLC1A2 | Down | -1.12E+00 | 2.67E+00 | -5.96E+00 | 5.88E-09 | 1.75E-08 |
| OTC | Down | -1.36E+00 | 6.26E+00 | -5.95E+00 | 6.17E-09 | 1.83E-08 |
| CYP2C8 | Down | -1.48E+00 | 6.69E+00 | -5.94E+00 | 6.38E-09 | 1.89E-08 |
| AKR7A3 | Down | -1.22E+00 | 5.56E+00 | -5.93E+00 | 7.02E-09 | 2.07E-08 |
| FABP4 | Down | -1.08E+00 | 3.11E+00 | -5.92E+00 | 7.42E-09 | 2.18E-08 |
| IGSF23 | Down | -1.06E+00 | 3.97E+00 | -5.89E+00 | 8.72E-09 | 2.54E-08 |
| HSD11B1 | Down | -1.77E+00 | 6.88E+00 | -5.88E+00 | 9.14E-09 | 2.65E-08 |
| PON1 | Down | -1.25E+00 | 7.11E+00 | -5.87E+00 | 9.86E-09 | 2.84E-08 |
| UPB1 | Down | -1.06E+00 | 5.34E+00 | -5.73E+00 | 2.03E-08 | 5.62E-08 |
| AFM | Down | -1.19E+00 | 6.40E+00 | -5.71E+00 | 2.29E-08 | 6.30E-08 |
| SULT2A1 | Down | -1.36E+00 | 8.23E+00 | -5.64E+00 | 3.45E-08 | 9.29E-08 |
| KNG1 | Down | -1.06E+00 | 1.00E+01 | -5.63E+00 | 3.47E-08 | 9.34E-08 |
| MT1X | Down | -1.27E+00 | 5.98E+00 | -5.59E+00 | 4.32E-08 | 1.14E-07 |
| CTH | Down | -1.03E+00 | 5.02E+00 | -5.59E+00 | 4.41E-08 | 1.17E-07 |
| CYP2C9 | Down | -1.31E+00 | 7.02E+00 | -5.59E+00 | 4.50E-08 | 1.19E-07 |
| RDH16 | Down | -1.26E+00 | 5.56E+00 | -5.59E+00 | 4.51E-08 | 1.19E-07 |
| CFHR5 | Down | -1.44E+00 | 5.63E+00 | -5.55E+00 | 5.40E-08 | 1.41E-07 |
| NAT2 | Down | -1.02E+00 | 2.65E+00 | -5.54E+00 | 5.77E-08 | 1.50E-07 |
| FTCD | Down | -1.07E+00 | 7.08E+00 | -5.44E+00 | 9.58E-08 | 2.42E-07 |
| MBL2 | Down | -1.10E+00 | 4.83E+00 | -5.44E+00 | 9.83E-08 | 2.48E-07 |
| ADH1A | Down | -1.18E+00 | 7.82E+00 | -5.43E+00 | 1.05E-07 | 2.63E-07 |
| CYP1A2 | Down | -1.56E+00 | 2.67E+00 | -5.37E+00 | 1.39E-07 | 3.46E-07 |
| CPS1 | Down | -1.50E+00 | 6.96E+00 | -5.31E+00 | 1.89E-07 | 4.60E-07 |
| GCGR | Down | -1.25E+00 | 3.16E+00 | -5.29E+00 | 2.05E-07 | 4.98E-07 |
| HRG | Down | -1.47E+00 | 8.61E+00 | -5.17E+00 | 3.83E-07 | 8.97E-07 |
| GNMT | Down | -1.21E+00 | 5.91E+00 | -5.13E+00 | 4.72E-07 | 1.09E-06 |
| PRAP1 | Down | -1.07E+00 | 8.93E+00 | -5.11E+00 | 5.16E-07 | 1.19E-06 |
| ALDH1L1 | Down | -1.09E+00 | 5.53E+00 | -5.07E+00 | 6.43E-07 | 1.46E-06 |
| ETNPPL | Down | -1.03E+00 | 4.68E+00 | -5.02E+00 | 8.12E-07 | 1.82E-06 |
| BHMT | Down | -1.28E+00 | 6.27E+00 | -4.98E+00 | 9.78E-07 | 2.17E-06 |
| C7 | Down | -1.16E+00 | 3.50E+00 | -4.85E+00 | 1.79E-06 | 3.84E-06 |
| ARG1 | Down | -1.09E+00 | 7.76E+00 | -4.76E+00 | 2.82E-06 | 5.91E-06 |
| SDS | Down | -1.49E+00 | 6.13E+00 | -4.58E+00 | 6.27E-06 | 1.26E-05 |
| APCS | Down | -1.01E+00 | 1.01E+01 | -4.54E+00 | 7.69E-06 | 1.53E-05 |
| HGFAC | Down | -1.28E+00 | 4.15E+00 | -4.53E+00 | 8.06E-06 | 1.60E-05 |
| THRSP | Down | -1.42E+00 | 4.07E+00 | -4.50E+00 | 8.94E-06 | 1.76E-05 |
| TTR | Down | -1.04E+00 | 1.01E+01 | -4.50E+00 | 8.95E-06 | 1.76E-05 |
| SAA1 | Down | -1.54E+00 | 8.37E+00 | -4.39E+00 | 1.49E-05 | 2.87E-05 |
| UGT2B10 | Down | -1.01E+00 | 6.78E+00 | -4.38E+00 | 1.53E-05 | 2.93E-05 |
| CFHR1 | Down | -1.05E+00 | 7.94E+00 | -4.34E+00 | 1.82E-05 | 3.44E-05 |
| APOA1 | Down | -1.09E+00 | 1.25E+01 | -4.33E+00 | 1.94E-05 | 3.66E-05 |
| CYP1A1 | Down | -1.14E+00 | 2.52E+00 | -4.32E+00 | 1.97E-05 | 3.72E-05 |
| HSD17B13 | Down | -1.39E+00 | 4.38E+00 | -4.30E+00 | 2.17E-05 | 4.07E-05 |
| CYP2A7 | Down | -1.21E+00 | 2.85E+00 | -4.26E+00 | 2.58E-05 | 4.80E-05 |
| APOA2 | Down | -1.04E+00 | 1.40E+01 | -4.26E+00 | 2.60E-05 | 4.83E-05 |
| SAA2-SAA4 | Down | -1.20E+00 | 3.99E+00 | -4.21E+00 | 3.16E-05 | 5.81E-05 |
| SAA2 | Down | -1.31E+00 | 5.21E+00 | -4.09E+00 | 5.34E-05 | 9.52E-05 |
| HAO2 | Down | -1.02E+00 | 4.21E+00 | -3.90E+00 | 1.14E-04 | 1.95E-04 |
| C9 | Down | -1.05E+00 | 5.38E+00 | -3.09E+00 | 2.13E-03 | 3.11E-03 |

**Supplementary Table 2.** DEGs of H2AFZ^high^ and H2AFZ^low^ group.

| **GO_BP (up)** | **Total** | **Expected** | **Hits** | **P.Value** | **FDR** |
| --- | --- | --- | --- | --- | --- |
| **Cell cycle phase** | 1070 | 20.7 | 134 | 1.19E-77 | 9.74E-75 |
| **Mitotic cell cycle** | 968 | 18.6 | 126 | 2.15E-74 | 8.8E-72 |
| **Cell cycle process** | 1420 | 27.2 | 143 | 2.91E-71 | 7.95E-69 |
| **Cell cycle** | 1860 | 35.8 | 158 | 1.34E-69 | 2.75E-67 |
| **M phase** | 671 | 12.9 | 95 | 2.4E-57 | 3.94E-55 |
| **M phase of mitotic cell cycle** | 447 | 8.6 | 80 | 9.69E-56 | 1.32E-53 |
| **Mitosis** | 420 | 8.08 | 77 | 2.38E-54 | 2.79E-52 |
| **Cell division** | 507 | 9.76 | 81 | 1.93E-52 | 1.97E-50 |
| **Regulation of cell cycle** | 886 | 17.1 | 79 | 3.51E-32 | 3.2E-30 |
| **Chromosome segregation** | 174 | 3.35 | 40 | 5.65E-32 | 4.63E-30 |
| **Interphase of mitotic cell cycle** | 435 | 8.37 | 56 | 1.3E-30 | 9.7E-29 |
| **Interphase** | 443 | 8.53 | 56 | 3.42E-30 | 2.34E-28 |
| **DNA metabolic process** | 1240 | 24 | 88 | 1.61E-28 | 1.01E-26 |
| **DNA replication** | 346 | 6.66 | 47 | 1.04E-26 | 6.08E-25 |
| **Cell cycle checkpoint** | 281 | 5.41 | 42 | 1.41E-25 | 7.7E-24 |
| **Microtubule_based process** | 516 | 9.93 | 54 | 5.24E-25 | 2.69E-23 |
| **Organelle organization** | 2820 | 54.2 | 128 | 3.21E-24 | 1.55E-22 |
| **Microtubule cytoskeleton organization** | 337 | 6.49 | 43 | 2.36E-23 | 1.08E-21 |
| **Cell cycle arrest** | 428 | 8.24 | 46 | 8.57E-22 | 3.7E-20 |
| **Chromosome organization** | 878 | 16.9 | 64 | 5.15E-21 | 2.11E-19 |
| **Sister chromatid segregation** | 57 | 1.1 | 20 | 1.56E-20 | 6.08E-19 |
| **DNA_dependent DNA replication** | 121 | 2.33 | 26 | 2.89E-20 | 1.08E-18 |
| **Mitotic sister chromatid segregation** | 54 | 1.04 | 19 | 1.36E-19 | 4.86E-18 |
| **Negative regulation of cell cycle** | 520 | 10 | 47 | 3.98E-19 | 1.36E-17 |
| **G1/S transition of mitotic cell cycle** | 209 | 4.02 | 31 | 5.76E-19 | 1.89E-17 |
| **Spindle organization** | 92 | 1.77 | 22 | 2.15E-18 | 6.79E-17 |
| **S phase** | 153 | 2.94 | 26 | 1.42E-17 | 4.32E-16 |
| **Regulation of mitotic cell cycle** | 351 | 6.76 | 36 | 1.58E-16 | 4.63E-15 |
| **DNA packaging** | 172 | 3.31 | 26 | 2.81E-16 | 7.94E-15 |
| **S phase of mitotic cell cycle** | 144 | 2.77 | 24 | 4.17E-16 | 1.14E-14 |
| **DNA replication initiation** | 30 | 0.577 | 13 | 3.35E-15 | 8.87E-14 |
| **Mitotic spindle organization** | 40 | 0.77 | 14 | 1.02E-14 | 2.62E-13 |
| **G2/M transition of mitotic cell cycle** | 150 | 2.89 | 22 | 1.14E-13 | 2.82E-12 |
| **Cytoskeleton organization** | 980 | 18.9 | 53 | 4.66E-12 | 1.12E-10 |
| **Regulation of mitosis** | 99 | 1.91 | 17 | 5.59E-12 | 1.31E-10 |
| **DNA repair** | 538 | 10.4 | 37 | 1.49E-11 | 3.39E-10 |
| **Response to DNA damage stimulus** | 862 | 16.6 | 48 | 2.06E-11 | 4.57E-10 |
| **Regulation of organelle organization** | 589 | 11.3 | 38 | 5.11E-11 | 1.1E-09 |
| **Cell proliferation** | 1900 | 36.6 | 76 | 1.77E-10 | 3.72E-09 |
| **DNA recombination** | 247 | 4.75 | 23 | 4.27E-10 | 8.75E-09 |
| **Chromosome condensation** | 34 | 0.654 | 10 | 5.17E-10 | 1.03E-08 |
| **Regulation of cyclin_dependent protein kinase activity** | 89 | 1.71 | 14 | 1.45E-09 | 2.82E-08 |
| **Protein_DNA complex assembly** | 143 | 2.75 | 17 | 2.17E-09 | 4.13E-08 |
| **Meiotic cell cycle** | 183 | 3.52 | 19 | 2.44E-09 | 4.54E-08 |
| **Chromatin assembly** | 128 | 2.46 | 16 | 3.11E-09 | 5.66E-08 |
| **Centrosome organization** | 70 | 1.35 | 12 | 8.02E-09 | 1.43E-07 |
| **Cytokinesis** | 120 | 2.31 | 15 | 9.76E-09 | 1.7E-07 |
| **Microtubule organizing center organization** | 74 | 1.42 | 12 | 1.54E-08 | 2.64E-07 |
| **Chromatin assembly or disassembly** | 152 | 2.93 | 16 | 3.77E-08 | 6.31E-07 |
| **Establishment of organelle localization** | 125 | 2.41 | 14 | 1.25E-07 | 2.05E-06 |
| **Mitotic cell cycle checkpoint** | 149 | 2.87 | 15 | 1.83E-07 | 2.94E-06 |
| **Chromatin remodeling** | 111 | 2.14 | 13 | 2.09E-07 | 3.3E-06 |
| **Centrosome cycle** | 47 | 0.905 | 9 | 2.28E-07 | 3.53E-06 |
| **Nucleosome assembly** | 112 | 2.16 | 13 | 2.33E-07 | 3.54E-06 |
| **DNA integrity checkpoint** | 152 | 2.93 | 15 | 2.38E-07 | 3.55E-06 |
| **Microtubule_based movement** | 179 | 3.45 | 16 | 3.68E-07 | 5.39E-06 |
| **Organelle localization** | 189 | 3.64 | 16 | 7.68E-07 | 1.1E-05 |
| **Cellular response to stress** | 1620 | 31.3 | 59 | 9.49E-07 | 1.34E-05 |
| **Phosphatidylinositol_mediated signaling** | 148 | 2.85 | 14 | 1.01E-06 | 1.41E-05 |
| **Maintenance of location in cell** | 112 | 2.16 | 12 | 1.62E-06 | 2.22E-05 |
| **Regulation of cytoskeleton organization** | 306 | 5.89 | 20 | 1.99E-06 | 2.68E-05 |
| **Maintenance of protein location** | 116 | 2.23 | 12 | 2.36E-06 | 3.12E-05 |
| **G1 phase of mitotic cell cycle** | 47 | 0.905 | 8 | 2.79E-06 | 3.63E-05 |
| **Maintenance of protein location in cell** | 101 | 1.94 | 11 | 3.77E-06 | 4.83E-05 |
| **G1 phase** | 49 | 0.943 | 8 | 3.87E-06 | 4.88E-05 |
| **Positive regulation of cell cycle** | 113 | 2.17 | 11 | 1.13E-05 | 0.00014 |
| **Double_strand break repair** | 139 | 2.68 | 12 | 1.55E-05 | 0.000189 |
| **Microtubule polymerization or depolymerization** | 59 | 1.14 | 8 | 1.61E-05 | 0.000194 |
| **DNA damage checkpoint** | 143 | 2.75 | 12 | 2.06E-05 | 0.000245 |
| **Base_excision repair** | 45 | 0.866 | 7 | 2.2E-05 | 0.000258 |
| **Negative regulation of cellular component organization** | 370 | 7.12 | 20 | 3.3E-05 | 0.000381 |
| **Macromolecular complex assembly** | 1120 | 21.6 | 41 | 5.01E-05 | 0.000571 |
| **Regulation of DNA metabolic process** | 235 | 4.52 | 15 | 5.1E-05 | 0.000573 |
| **Response to UV** | 112 | 2.16 | 10 | 5.96E-05 | 0.000661 |
| **Meiosis I** | 91 | 1.75 | 9 | 6.28E-05 | 0.000687 |
| **Maintenance of location** | 192 | 3.7 | 13 | 8.92E-05 | 0.000962 |
| **Cellular component disassembly** | 310 | 5.97 | 17 | 0.000109 | 0.00116 |
| **Regulation of cellular component organization** | 1520 | 29.3 | 49 | 0.000213 | 0.00224 |
| **Regulation of protein modification process** | 1250 | 24 | 42 | 0.000249 | 0.00259 |
| **Chromatin organization** | 637 | 12.3 | 26 | 0.000252 | 0.00259 |
| **Cellular protein complex assembly** | 343 | 6.6 | 17 | 0.000359 | 0.00363 |
| **Response to radiation** | 345 | 6.64 | 17 | 0.000384 | 0.00384 |
| **Protein complex assembly** | 861 | 16.6 | 31 | 0.000574 | 0.00568 |
| **Regulation of cell proliferation** | 1430 | 27.5 | 45 | 0.000619 | 0.00604 |
| **Negative regulation of cytoskeleton organization** | 80 | 1.54 | 7 | 0.000869 | 0.00838 |
| **Chromatin modification** | 512 | 9.85 | 21 | 0.000936 | 0.00893 |
| **Regulation of DNA replication** | 110 | 2.12 | 8 | 0.00128 | 0.0121 |
| **Protein polymerization** | 167 | 3.21 | 10 | 0.00148 | 0.0138 |
| **Response to light stimulus** | 229 | 4.41 | 12 | 0.00163 | 0.015 |
| **Reproduction** | 1860 | 35.7 | 53 | 0.00189 | 0.0172 |
| **Regulation of protein kinase activity** | 698 | 13.4 | 25 | 0.00212 | 0.0191 |
| **Regulation of kinase activity** | 743 | 14.3 | 26 | 0.00238 | 0.0212 |
| **Regulation of chromosome organization** | 122 | 2.35 | 8 | 0.00247 | 0.0218 |
| **Regulation of transferase activity** | 768 | 14.8 | 26 | 0.00371 | 0.0324 |
| **Aging** | 197 | 3.79 | 10 | 0.00489 | 0.0422 |
| **Response to steroid hormone stimulus** | 267 | 5.14 | 12 | 0.00567 | 0.0484 |
| **Response to abiotic stimulus** | 876 | 16.9 | 28 | 0.00579 | 0.0489 |
| **Response to ionizing radiation** | 112 | 2.16 | 7 | 0.0059 | 0.0494 |
| **GO_BP (down)** | **Total** | **Expected** | **Hits** | **P.Value** | **FDR** |
| **Xenobiotic metabolic process** | 165 | 0.97 | 15 | 3.39E-14 | 1.98E-11 |
| **Response to xenobiotic stimulus** | 169 | 0.994 | 15 | 4.84E-14 | 1.98E-11 |
| **Organic acid metabolic process** | 1430 | 8.4 | 29 | 9.45E-10 | 0.000000258 |
| **Carboxylic acid metabolic process** | 1270 | 7.49 | 27 | 1.76E-09 | 0.00000036 |
| **Response to drug** | 344 | 2.02 | 14 | 1.17E-08 | 0.00000192 |
| **Response to chemical stimulus** | 3830 | 22.5 | 47 | 1.51E-08 | 0.00000207 |
| **Steroid metabolic process** | 361 | 2.12 | 14 | 2.15E-08 | 0.00000252 |
| **Humoral immune response** | 157 | 0.923 | 10 | 2.65E-08 | 0.00000262 |
| **Acute inflammatory response** | 118 | 0.694 | 9 | 2.88E-08 | 0.00000262 |
| **Triglyceride metabolic process** | 126 | 0.741 | 9 | 0.000000051 | 0.00000418 |
| **Monocarboxylic acid metabolic process** | 567 | 3.33 | 16 | 0.00000016 | 0.0000115 |
| **Inflammatory response** | 569 | 3.35 | 16 | 0.000000168 | 0.0000115 |
| **Cellular lipid metabolic process** | 1200 | 7.07 | 22 | 0.00000109 | 0.0000689 |
| **Response to wounding** | 1310 | 7.72 | 23 | 0.00000123 | 0.000072 |
| **Lipid catabolic process** | 305 | 1.79 | 11 | 0.00000163 | 0.000089 |
| **Isoprenoid metabolic process** | 101 | 0.594 | 7 | 0.00000207 | 0.000106 |
| **Lipid homeostasis** | 103 | 0.606 | 7 | 0.00000237 | 0.000114 |
| **Alcohol metabolic process** | 388 | 2.28 | 12 | 0.00000263 | 0.00012 |
| **Lipid metabolic process** | 1730 | 10.2 | 26 | 0.00000364 | 0.000157 |
| **Cellular amino acid catabolic process** | 166 | 0.976 | 8 | 0.00000578 | 0.000237 |
| **Response to hormone stimulus** | 751 | 4.42 | 16 | 0.00000645 | 0.000252 |
| **Catabolic process** | 2560 | 15.1 | 32 | 0.0000101 | 0.000375 |
| **Defense response** | 1510 | 8.87 | 23 | 0.0000129 | 0.00046 |
| **Cellular catabolic process** | 2140 | 12.6 | 28 | 0.0000208 | 0.000709 |
| **Response to nutrient** | 147 | 0.864 | 7 | 0.0000247 | 0.000789 |
| **Carbohydrate biosynthetic process** | 203 | 1.19 | 8 | 0.000025 | 0.000789 |
| **Lipid transport** | 268 | 1.58 | 9 | 0.0000271 | 0.000823 |
| **Immune effector process** | 576 | 3.39 | 13 | 0.0000293 | 0.000857 |
| **Adaptive immune response based on somatic recombination of immune receptors built from immunoglobulin superfamily domains** | 218 | 1.28 | 8 | 0.0000416 | 0.00118 |
| **Glutamine family amino acid metabolic process** | 75 | 0.441 | 5 | 0.0000778 | 0.00213 |
| **Adaptive immune response** | 241 | 1.42 | 8 | 0.0000843 | 0.00223 |
| **Response to extracellular stimulus** | 320 | 1.88 | 9 | 0.000107 | 0.00274 |
| **Chemical homeostasis** | 1050 | 6.2 | 17 | 0.000113 | 0.00282 |
| **Cellular lipid catabolic process** | 188 | 1.11 | 7 | 0.000118 | 0.00284 |
| **Secondary metabolic process** | 44 | 0.259 | 4 | 0.000126 | 0.00288 |
| **Positive regulation of immune response** | 487 | 2.86 | 11 | 0.000126 | 0.00288 |
| **Cellular amino acid metabolic process** | 670 | 3.94 | 13 | 0.000136 | 0.00292 |
| **Cellular amino acid metabolic process** | 670 | 3.94 | 13 | 0.000136 | 0.00292 |
| **Cellular carbohydrate metabolic process** | 259 | 1.52 | 8 | 0.000139 | 0.00292 |
| **Energy reserve metabolic process** | 199 | 1.17 | 7 | 0.000167 | 0.00342 |
| **Response to steroid hormone stimulus** | 267 | 1.57 | 8 | 0.000171 | 0.00342 |
| **Cdc42 protein signal transduction** | 4 | 0.0235 | 2 | 0.000203 | 0.00397 |
| **Regulation of biological quality** | 3400 | 20 | 35 | 0.000212 | 0.00404 |
| **Glucose metabolic process** | 290 | 1.7 | 8 | 0.0003 | 0.00554 |
| **Regulation of immune response** | 727 | 4.27 | 13 | 0.000304 | 0.00554 |
| **Response to nutrient levels** | 295 | 1.73 | 8 | 0.000336 | 0.00599 |
| **Regulation of response to external stimulus** | 461 | 2.71 | 10 | 0.000361 | 0.00631 |
| **Activation of immune response** | 399 | 2.35 | 9 | 0.000547 | 0.00934 |
| **Organic acid transport** | 246 | 1.45 | 7 | 0.000604 | 0.00991 |
| **Carboxylic acid transport** | 246 | 1.45 | 7 | 0.000604 | 0.00991 |
| **Negative regulation of hydrolase activity** | 253 | 1.49 | 7 | 0.000713 | 0.0114 |
| **Cofactor metabolic process** | 331 | 1.95 | 8 | 0.00072 | 0.0114 |
| **Response to organic substance** | 2500 | 14.7 | 27 | 0.000777 | 0.012 |
| **Response to endogenous stimulus** | 1360 | 8.02 | 18 | 0.000818 | 0.0124 |
| **Response to external stimulus** | 1510 | 8.88 | 19 | 0.00102 | 0.0152 |
| **Glucan metabolic process** | 95 | 0.559 | 4 | 0.00236 | 0.0345 |
| **Sulfur compound metabolic process** | 313 | 1.84 | 7 | 0.00243 | 0.0346 |
| **Negative regulation of cell adhesion** | 96 | 0.564 | 4 | 0.00245 | 0.0346 |
| **Homeostatic process** | 1510 | 8.85 | 18 | 0.00252 | 0.035 |
| **Generation of precursor metabolites and energy** | 603 | 3.55 | 10 | 0.00276 | 0.0375 |
| **Negative regulation of multicellular organismal process** | 410 | 2.41 | 8 | 0.00279 | 0.0375 |
| **Cellular modified amino acid metabolic process** | 241 | 1.42 | 6 | 0.00294 | 0.0389 |
| **Regulation of lipid metabolic process** | 246 | 1.45 | 6 | 0.00325 | 0.0423 |
| **Secretion** | 947 | 5.57 | 13 | 0.0034 | 0.0436 |
| **Regulation of immune effector process** | 249 | 1.46 | 6 | 0.00345 | 0.0436 |
| **Positive regulation of immune system process** | 739 | 4.34 | 11 | 0.00389 | 0.0483 |
| **Energy derivation by oxidation of organic compounds** | 437 | 2.57 | 8 | 0.00411 | 0.0497 |
| **Innate immune response** | 638 | 3.75 | 10 | 0.00412 | 0.0497 |

**Supplementary Table 3.** Significant GO-BP annotations of DEGs.

| **KEGG (up)** | **Total** | **Expected** | **Hits** | **P.Value** | **FDR** |
| --- | --- | --- | --- | --- | --- |
| **Cell cycle** | 124 | 2.31 | 38 | 3.36E-37 | 1.07E-34 |
| **DNA replication** | 36 | 0.67 | 14 | 8.45E-16 | 1.34E-13 |
| **Oocyte meiosis** | 125 | 2.33 | 16 | 9.89E-10 | 1.05E-07 |
| **Progesterone-mediated oocyte maturation** | 99 | 1.84 | 14 | 3.11E-09 | 2.47E-07 |
| **Cellular senescence** | 160 | 2.98 | 17 | 5.33E-09 | 3.39E-07 |
| **p53 signaling pathway** | 72 | 1.34 | 11 | 7.24E-08 | 3.84E-06 |
| **Fanconi anemia pathway** | 54 | 1.01 | 9 | 5.44E-07 | 2.47E-05 |
| **HTLV-I infection** | 219 | 4.08 | 16 | 2.73E-06 | 0.000103 |
| **Mismatch repair** | 23 | 0.428 | 6 | 2.91E-06 | 0.000103 |
| **MicroRNAs in cancer** | 299 | 5.56 | 17 | 3.68E-05 | 0.00117 |
| **Biosynthesis of amino acids** | 75 | 1.4 | 8 | 6.98E-05 | 0.00202 |
| **Base excision repair** | 33 | 0.614 | 5 | 0.000325 | 0.00861 |
| **Homologous recombination** | 41 | 0.763 | 5 | 0.00091 | 0.0207 |
| **Bladder cancer** | 41 | 0.763 | 5 | 0.00091 | 0.0207 |
| **Viral carcinogenesis** | 201 | 3.74 | 11 | 0.00127 | 0.0269 |
| **Small cell lung cancer** | 93 | 1.73 | 7 | 0.00167 | 0.0331 |
| **Endocrine resistance** | 98 | 1.82 | 7 | 0.00226 | 0.0422 |
| **Pancreatic cancer** | 75 | 1.4 | 6 | 0.00263 | 0.0464 |
| **KEGG (down)** | **Total** | **Expected** | **Hits** | **P.Value** | **FDR** |
| **Retinol metabolism** | 67 | 0.58 | 14 | 2.18E-16 | 6.93E-14 |
| **Chemical carcinogenesis** | 82 | 0.71 | 13 | 1.27E-13 | 2.03E-11 |
| **Metabolism of xenobiotics by cytochrome P450** | 76 | 0.658 | 12 | 1.28E-12 | 1.36E-10 |
| **Complement and coagulation cascades** | 79 | 0.684 | 12 | 2.07E-12 | 1.65E-10 |
| **Metabolic pathways** | 1430 | 12.4 | 37 | 1.55E-11 | 9.26E-10 |
| **Drug metabolism - cytochrome P450** | 72 | 0.623 | 11 | 1.75E-11 | 9.26E-10 |
| **PPAR signaling pathway** | 74 | 0.641 | 10 | 5.5E-10 | 0.000000025 |
| **Tyrosine metabolism** | 36 | 0.312 | 6 | 0.000000533 | 0.0000212 |
| **Glycolysis / Gluconeogenesis** | 68 | 0.589 | 7 | 0.00000169 | 0.0000599 |
| **Caffeine metabolism** | 5 | 0.0433 | 3 | 0.00000613 | 0.000195 |
| **Glucagon signaling pathway** | 103 | 0.892 | 7 | 0.0000273 | 0.000788 |
| **Fatty acid degradation** | 44 | 0.381 | 5 | 0.000035 | 0.000928 |
| **Biosynthesis of amino acids** | 75 | 0.649 | 6 | 0.0000423 | 0.00104 |
| **Linoleic acid metabolism** | 29 | 0.251 | 4 | 0.000104 | 0.00235 |
| **Steroid hormone biosynthesis** | 60 | 0.52 | 5 | 0.000158 | 0.00335 |
| **Prion diseases** | 35 | 0.303 | 4 | 0.00022 | 0.00437 |
| **Glycine, serine and threonine metabolism** | 40 | 0.346 | 4 | 0.000371 | 0.00648 |
| **Bile secretion** | 72 | 0.623 | 5 | 0.000374 | 0.00648 |
| **Phenylalanine metabolism** | 17 | 0.147 | 3 | 0.000387 | 0.00648 |
| **Drug metabolism - other enzymes** | 79 | 0.684 | 5 | 0.000575 | 0.00915 |
| **Cysteine and methionine metabolism** | 47 | 0.407 | 4 | 0.000692 | 0.0105 |
| **Arginine biosynthesis** | 21 | 0.182 | 3 | 0.000738 | 0.0107 |
| **Arachidonic acid metabolism** | 63 | 0.546 | 4 | 0.00208 | 0.0288 |
| **Insulin resistance** | 108 | 0.935 | 5 | 0.00235 | 0.0311 |
| **Fructose and mannose metabolism** | 33 | 0.286 | 3 | 0.00281 | 0.0358 |
| **AMPK signaling pathway** | 120 | 1.04 | 5 | 0.00371 | 0.0444 |
| **Ubiquinone and other terpenoid-quinone biosynthesis** | 11 | 0.0953 | 2 | 0.00386 | 0.0444 |
| **African trypanosomiasis** | 37 | 0.32 | 3 | 0.00391 | 0.0444 |
| **Tryptophan metabolism** | 42 | 0.364 | 3 | 0.0056 | 0.0614 |

**Supplementary Table 4.** Significant KEGG pathway annotations of DEGs.

| **Transcriptional factor** | **Total** | **Expected** | **Hits** | **P.Value** | **FDR** |
| --- | --- | --- | --- | --- | --- |
| **V$E2F1_Q6** | 232 | 14.3 | 58 | 1.22E-20 | 1.02E-17 |
| **V$E2F_Q4** | 234 | 14.5 | 57 | 1.02E-19 | 4.25E-17 |
| **V$E2F_Q6** | 232 | 14.3 | 56 | 3.41E-19 | 9.49E-17 |
| **V$E2F4DP1_01** | 239 | 14.8 | 56 | 1.5E-18 | 3.13E-16 |
| **V$E2F_02** | 235 | 14.5 | 55 | 3.24E-18 | 3.38E-16 |
| **V$E2F1DP1_01** | 235 | 14.5 | 55 | 3.24E-18 | 3.38E-16 |
| **V$E2F1DP2_01** | 235 | 14.5 | 55 | 3.24E-18 | 3.38E-16 |
| **V$E2F4DP2_01** | 235 | 14.5 | 55 | 3.24E-18 | 3.38E-16 |
| **V$E2F_Q6_01** | 240 | 14.8 | 54 | 4.25E-17 | 3.95E-15 |
| **V$E2F_Q4_01** | 237 | 14.7 | 53 | 1.11E-16 | 9.27E-15 |
| **V$E2F1DP1RB_01** | 231 | 14.3 | 52 | 1.61E-16 | 1.23E-14 |
| **V$E2F1_Q4_01** | 228 | 14.1 | 49 | 8.61E-15 | 6E-13 |
| **V$E2F_Q3_01** | 235 | 14.5 | 49 | 2.99E-14 | 1.92E-12 |
| **V$E2F1_Q6_01** | 238 | 14.7 | 49 | 5E-14 | 2.99E-12 |
| **SGCGSSAAA_V$E2F1DP2_01** | 168 | 10.4 | 38 | 1.75E-12 | 9.73E-11 |
| **V$E2F1_Q3** | 244 | 15.1 | 47 | 2.11E-12 | 1.1E-10 |
| **V$E2F_03** | 245 | 15.2 | 47 | 2.47E-12 | 1.21E-10 |
| **V$E2F_Q3** | 227 | 14 | 44 | 8.68E-12 | 4.03E-10 |
| **GATTGGY_V$NFY_Q6_01** | 1160 | 71.7 | 125 | 3.01E-10 | 1.33E-08 |
| **V$E2F1_Q4** | 244 | 15.1 | 43 | 3.66E-10 | 1.53E-08 |
| **KTGGYRSGAA_UNKNOWN** | 76 | 4.7 | 20 | 2.15E-08 | 8.57E-07 |
| **V$E2F_01** | 67 | 4.14 | 18 | 7.71E-08 | 2.93E-06 |
| **GGGCGGR_V$SP1_Q6** | 2940 | 182 | 238 | 1.63E-06 | 5.91E-05 |
| **V$NFE2_01** | 272 | 16.8 | 37 | 5.02E-06 | 0.000175 |
| **V$NRF2_Q4** | 255 | 15.8 | 34 | 1.85E-05 | 0.000618 |
| **CATTGTYY_V$SOX9_B1** | 358 | 22.1 | 43 | 2.17E-05 | 0.000698 |
| **V$E2F1_Q3_01** | 247 | 15.3 | 33 | 2.34E-05 | 0.000725 |
| **GGGAGGRR_V$MAZ_Q6** | 2270 | 141 | 184 | 3.94E-05 | 0.00118 |
| **TGANNYRGCA_V$TCF11MAFG_01** | 301 | 18.6 | 37 | 4.9E-05 | 0.00141 |
| **TGANTCA_V$AP1_C** | 1120 | 69.3 | 101 | 6.48E-05 | 0.00181 |
| **V$AP1_01** | 267 | 16.5 | 33 | 0.000111 | 0.003 |
| **TGASTMAGC_V$NFE2_01** | 195 | 12.1 | 26 | 0.000173 | 0.00451 |
| **V$NFY_01** | 254 | 15.7 | 31 | 0.000223 | 0.00564 |
| **V$AP1_Q4** | 271 | 16.8 | 32 | 0.000324 | 0.00797 |
| **CCAWYNNGAAR_UNKNOWN** | 145 | 8.97 | 20 | 0.000604 | 0.0144 |
| **V$SP1_Q4_01** | 258 | 16 | 30 | 0.000636 | 0.0148 |
| **RRCCGTTA_UNKNOWN** | 87 | 5.38 | 14 | 0.000851 | 0.0192 |
| **YGTCCTTGR_UNKNOWN** | 98 | 6.06 | 15 | 0.000964 | 0.0212 |
| **V$SP1_Q6** | 256 | 15.8 | 29 | 0.00118 | 0.0252 |
| **GCGNNANTTCC_UNKNOWN** | 123 | 7.61 | 17 | 0.00147 | 0.0304 |
| **V$STAT1_03** | 248 | 15.3 | 28 | 0.00149 | 0.0304 |
| **V$AP1_Q4_01** | 261 | 16.1 | 29 | 0.00159 | 0.0316 |
| **ACTAYRNNNCCCR_UNKNOWN** | 449 | 27.8 | 44 | 0.00164 | 0.0319 |
| **V$MYB_Q3** | 250 | 15.5 | 28 | 0.00168 | 0.0319 |
| **V$MAZ_Q6** | 193 | 11.9 | 23 | 0.00189 | 0.0351 |
| **CRGAARNNNNCGA_UNKNOWN** | 47 | 2.91 | 9 | 0.00206 | 0.0374 |
| **V$AP1FJ_Q2** | 268 | 16.6 | 29 | 0.00236 | 0.0415 |
| **V$CREL_01** | 256 | 15.8 | 28 | 0.00238 | 0.0415 |
| **V$AP1_Q6** | 259 | 16 | 28 | 0.00282 | 0.0476 |
| **V$ZIC2_01** | 247 | 15.3 | 27 | 0.00285 | 0.0476 |
| **GGGTGGRR_V$PAX4_03** | 1290 | 80 | 104 | 0.00295 | 0.0483 |
| **GGGYGTGNY_UNKNOWN** | 664 | 41.1 | 59 | 0.00303 | 0.0487 |
| **ARGGGTTAA_UNKNOWN** | 121 | 7.48 | 16 | 0.00316 | 0.0497 |
| **V$NFKAPPAB65_01** | 237 | 14.7 | 26 | 0.00321 | 0.0497 |

**Supplementary Table 5.** Significant transcriptional factor (TF) annotations of DEGs.
