# Supplementary figures and images for: H2AFZ Is a Prognostic Biomarker Correlated to TP53 Mutation and Immune Infiltration in Hepatocellular Carcinoma
Source: Front Oncol. 2021 Oct 25;11:701736. doi: 10.3389/fonc.2021.701736 (PMC8573175; doi:10.3389/fonc.2021.701736)

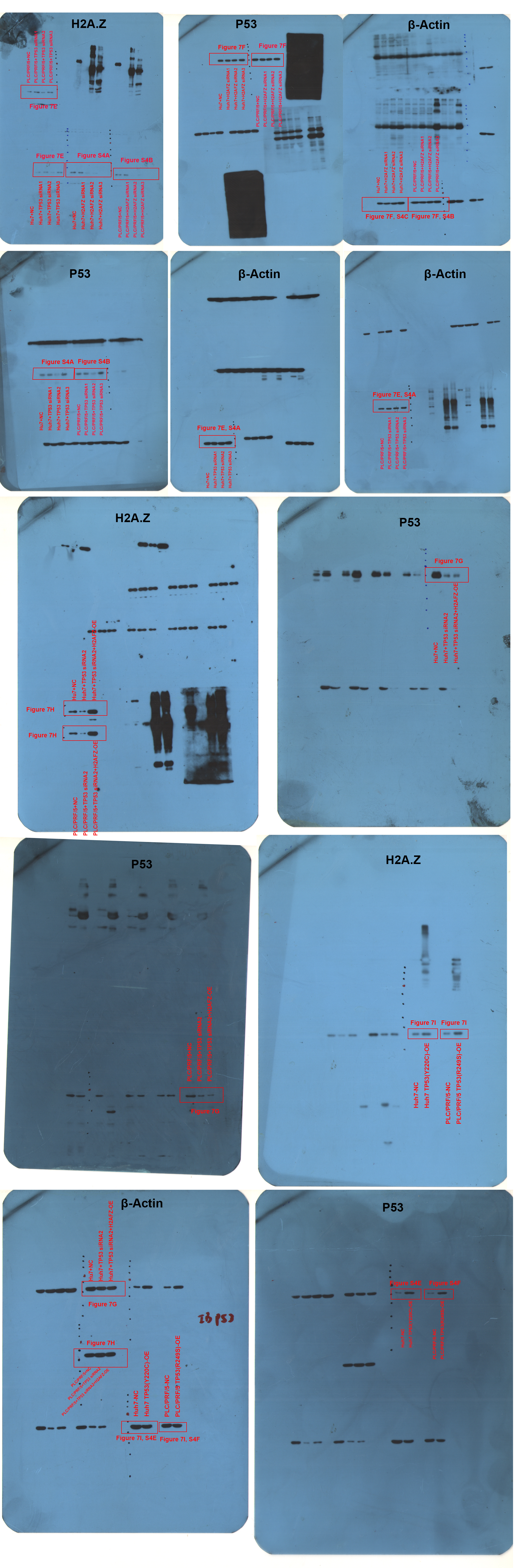

Supplement: Supplementary file 2 [file Image_1.jpeg]
